# Metabolomic Profiling of *Guadua* Species and Its Correlation with Antioxidant and Cytotoxic Activities

**DOI:** 10.1021/acsomega.3c09114

**Published:** 2024-08-21

**Authors:** Luis Carlos Chitiva, Paula Rezende-Teixeira, Tiago F. Leão, Hair Santiago Lozano-Puentes, Ximena Londoño, Lucía Ana Díaz-Ariza, Leticia V. Costa-Lotufo, Juliet A. Prieto-Rodríguez, Geison M. Costa, Ian Castro-Gamboa

**Affiliations:** † Núcleo de Bioensaios, Biossíntese e Ecofisiologia de Produtos Naturais (NuBBE), Institute of Chemistry, São Paulo State University (UNESP), Araraquara, São Paulo 14800-901, Brazil; ‡ Grupo de Investigación Fitoquímica Universidad Javeriana (GIFUJ), Department of Chemistry, Faculty of Sciences, Pontificia Universidad Javeriana, Bogotá 110231, Colombia; § Laboratório de Farmacologia de Produtos Naturais Marinhos, Institute of Biomedical Sciences, University of São Paulo, São Paulo, São Paulo 05508-000, Brazil; ∥ Laboratorio Asociaciones, Suelo, Planta, Microorganismo (LAMIC), Grupo de Investigación en Agricultura Biológica, Department of Biology, Faculty of Sciences, Pontificia Universidad Javeriana, Bogotá 110231, Colombia; ⊥ Faculty of Agricultural Sciences, Universidad Nacional de Colombia, Palmira 763533, Colombia

## Abstract

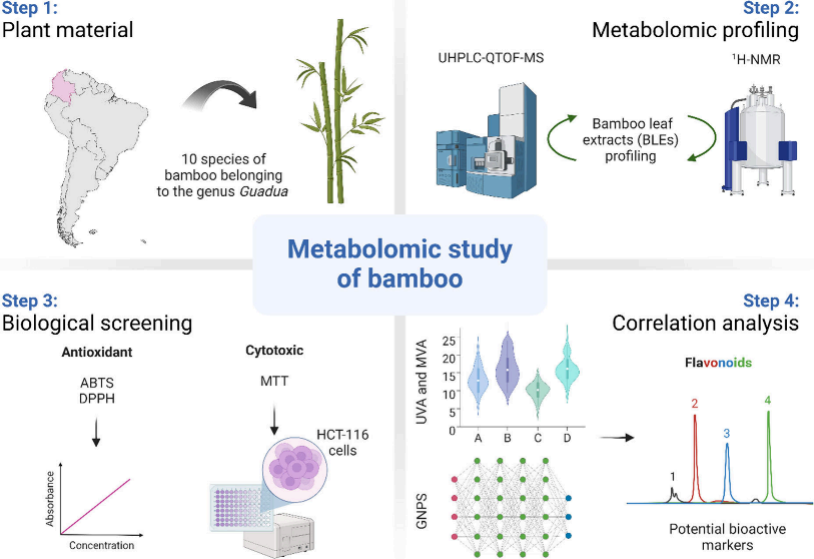

Bamboo plants are widely used in Asian traditional medicine for various health issues and exhibit therapeutic potential. *Guadua* species are renowned bamboos for their high phenolic compound content, including flavonoids and hydroxycinnamic acid derivatives, and possess noteworthy biological properties. Despite this, there is a notable scarcity of research on the chemical and biological aspects of Latin American bamboo leaf extracts (BLEs), especially concerning the *Guadua* genus. This study aimed to employ a metabolomics approach to integrate the phytochemical and activity profiles of BLEs to identify potential bioactive markers. We determined the metabolic fingerprints of 30 BLEs through HPTLC, HPLC-DAD, UHPLC-QTOF-MS, and ^1^H-NMR analyses and screened for antioxidant and cytotoxic activities using ABTS, DPPH, and MTT methods. Ultimately, correlation analyses were performed by using chemometric methods and molecular networking. Our findings present a comprehensive chemical characterization, encompassing 40 flavonoids and 9 cinnamic acid derivatives. Notably, most of these compounds have been reported for the first time within the genus, signifying novel discoveries. Additionally, certain compounds identified in other species of the subfamily Bambusoideae provide valuable comparative insights. These compounds demonstrated a significant correlation with antioxidant potential, with values exceeding 100 and 30 μmol of TE/g of extract for ABTS and DPPH, respectively, in the samples. Extracts from *G. incana* and *G. angustifolia* exhibited potent cytotoxic effects with IC_50_ values of 1.23 and 4.73 μg/mL against HCT-116 colon cancer cells, respectively. Notably, glycosylated flavones showed a strong correlation with cytotoxicity. These new findings significantly contribute to our understanding of the chemical composition and biological properties of these often overlooked bamboo species, providing them with important added value and alternative use.

## 1 Introduction

Bamboos, belonging to the subfamily Bambusoideae (Poaceae), comprise 1670 species distributed across 125 genera and divided into three tribes: herbaceous bamboos (Olyreae), tropical woody bamboos (Bambuseae), and temperate woody bamboos (Arundinarieae). They are distributed across various geographic regions, predominantly in Asia and Latin America.^1,2^ These plants, known as “a God’s gift” or “the plant of thousand uses”, have traditionally been used in Asia for their medicinal properties to address various health conditions including allergies, respiratory diseases, acute infections, dermatitis, diabetes, hair growth promotion, acne alleviation, hypertension management, fever reduction, detoxification assistance, and potential treatment for certain forms of cancer.^3−11^

Attractively, the Neotropical woody bamboos of the *Guadua* genus, often referred to as the “vegetable steel” or the “green gold of the 21st century”, lack a history of traditional medicinal use. They are regarded as the most significant American bamboos, comprising 33 species distributed across tropical regions in Central and South America.^2^ Despite their prominence in Latin America, comprehensive knowledge of their chemical composition and biological potential remains scarce. Preliminary phytochemical studies indicate the presence of flavonoids, phenolic acid derivatives, terpenes, saponins, and alkaloids.^12−15^ Subsequent investigations have focused on investigations into the influence of altitude on the chemical composition of bamboos have revealed intriguing patterns with higher altitude species exhibiting increased concentrations of flavonoids.^16−20^ These revealed phytochemicals have been reported to possess different biological activities, e.g., antimicrobial,^13^ antioxidant,^17,21^ anti-inflammatory,^22,23^ and anticancer effects.^8,24^ Despite these promising initial insights, a significant knowledge gap exists concerning the detailed relationship between the chemical compositions of *Guadua* species and their biological activities. Our study seeks to bridge this gap by conducting an in-depth investigation, utilizing a metabolomic approach to establish a correlation between chemical fingerprinting and biological activity in these bamboos. The primary objective is to identify chemical markers with antioxidant and cytotoxic potential, providing a more comprehensive and applicable understanding of these species in the realms of medicine and beyond. It is noteworthy that metabolomic research on *Guadua* species, particularly concerning compounds responsible for antioxidant and cytotoxic activities, is notably absent in the current scientific literature. Our study represents a significant step toward filling this void, offering valuable insights and laying the groundwork for future applications. This research endeavor not only contributes to the scientific community’s understanding of bamboos but also opens new perspectives for their utilization in various fields.

**Figure 1:**
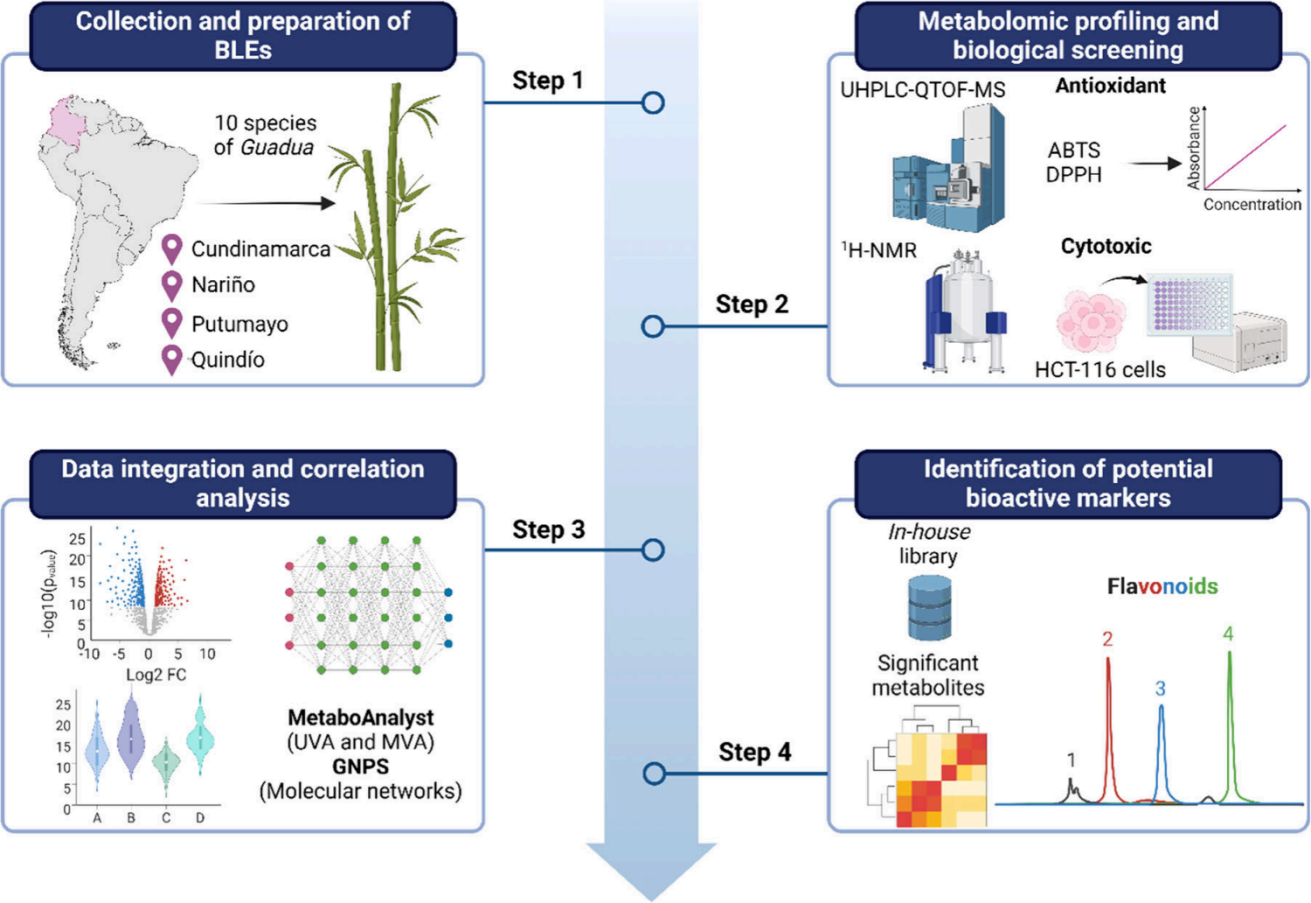
Workflow of the metabolomics strategy implemented for the identification of candidate bioactive compounds. Adapted from “T Cell Culture Workflow”, by BioRender.com. Retrieved from https://app.biorender.com/biorender-templates/figures/all/t-62ccc6c3da5217036d07073b-t-cell-culture-workflow.

Therefore, this study aimed to integrate the chemical and bioactivity profiles of 30 BLEs based on a metabolomics approach for the identification of potential bioactive markers. Figure 1 illustrates the workflow used in this research.

## 2 Results

### 2.1 HPTLC and HPLC-DAD Phytochemical Profiling Analysis

**Figure 2:**
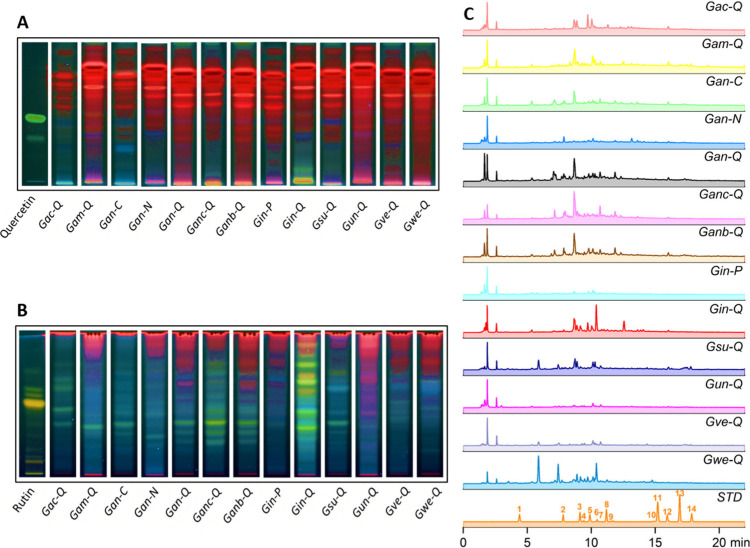
HPTLC chromatographic profiles for (A) flavonoid aglycones and (B) flavonoid glycosides. HPLC-PDA chromatograms of the BLEs were expanded from 0 to 23 min at (C) 274 nm. Chemical standards (STD): Gallic acid (**1**, *t*_*R*_ = 4.36 min), Chlorogenic acid (**2**, *t*_*R*_ = 7.72 min), Caffeic acid (**3**, *t*_*R*_ = 9.08 min), Isoorientin (**4**, *t*_*R*_ = 9.39 min), Ampelopsin (**5**, *t*_*R*_ = 9.88 min), Rutin (**6**, *t*_*R*_ = 10.42 min), Vitexin (**7**, *t*_*R*_ = 10.56 min), *p*-Coumaric acid (**8**, *t*_*R*_ = 11.15 min), Sinapinic acid (**9**, *t*_*R*_ = 11.55 min), Morin (**10**, *t*_*R*_ = 14.79 min), Coumarin (**11**, *t*_*R*_ = 15.19 min), Quercetin (**12**, *t*_*R*_ = 15.86 min), Cinnamic acid (**13**, *t*_*R*_ = 16.84 min), and Naringenin (**14**, *t*_*R*_ = 17.75 min). Column: Luna C18 column (150 mm × 4.6 mm, 5 μm, 100 Å); mobile phase: (A) 0.1% formic acid in water and (B) 0.1% formic acid in acetonitrile, 5–40% B (0–23 min); flow rate: 1 mL/min; detection wavelength: 274 nm. UV spectra were obtained via DAD detection. Chemical profiles were plotted by selecting one sample for each collection site and the different species (*n* = 13) according to the coding in Table 2.

A previous study conducted on bamboos belonging to the genus *Guadua*, using an untargeted metabolomics approach to determine the changes in chemical composition under the influence of altitudinal variation, showed that these bamboos have a high chemical composition, specifically of phenolic compounds, encompassing flavonoids in both their aglycone and glycosidic forms, accounting for 48% of their total chemical makeup.^16^ These findings enabled us to conduct targeted chemical profiling in this study, aiming to dereplication for known compounds using specific commercial chemical standards related to flavonoids and phenolic acid derivatives (**1**–**14**) via HPTLC and HPLC-DAD. Figure 2A and B show the chromatographic profiles obtained by HPTLC for the BLEs collected at 13 distinct locations, as detailed in Table 2.

Figure 2A presents the chemical profile of the flavonoid aglycones, while Figure 2B presents the flavonoid glycosides alongside the reference standards Quercetin (**12**) and Rutin (**6**), respectively. Moreover, the plates were eluted based on the polarity of these compounds, then sprayed with the natural products-polyethyleneglycol reagent (NP/PEG) and developed at a wavelength of UV-365 nm.^25^ From the results, it is evident that the extracts contain a limited number of flavonoid aglycones, while a higher quantity of flavonoid glycosides is emphasized, as confirmed by the presence of yellow and orange fluorescent bands. The retardation factor (*R*_*f*_) values for reference standards **12** and **6** were 0.58 and 0.62, respectively. *G. incana* Londoño (*Gin-Q*) is the species that exhibits a significant number of flavonoid glycosides compared to the other species, which are found in lower proportions. In this study, it was observed that flavonoid glycosides exhibit considerable variability among different extracts and are distinguished based on collection sites. This finding is consistent with earlier preliminary studies that revealed a higher accumulation of these compounds in these bamboos considered as giant grasses.^12^

Figure 2C shows the chromatographic profiles obtained by HPLC-DAD for the BLEs. We emphasize that between 5 and 12 min, a significant portion of the chemical composition of these extracts is observed, primarily comprising flavonoid glycosides and phenolic acid derivatives. The identification of these compounds was confirmed through ultraviolet spectra analysis (Supporting Information Figure S1). Most of the peaks exhibited two distinctive absorption bands characteristic of flavonoids, corresponding to band I (240–285 nm) and band II (300–560 nm).^26,27^ The presence of phenolic acid derivatives was also determined by the characteristic band with a shoulder (sh) between 290 and 310 nm.^28^ From the dereplication study of BLEs, chlorogenic acid (CGA) (**2**) was identified in the *G. weberbaueri* Pilg. species with a retention time (*t*_*R*_ = 7.72 min) and an ultraviolet spectrum with absorption at 326 nm and a shoulder peak at 292 nm, which was compared with the commercial chemical standard and reports in the literature.^29^ This compound is reported for the first time for this species. However, it has been previously reported in other bamboo species.^30,31^ The remaining reference compounds were not found in any of the other species. Nevertheless, it can be inferred that these species may contain compounds related to flavonoid glycoside and phenolic acid derivative scaffolds based on the absorption bands observed in the ultraviolet spectra (Supporting Information Figure S1).

### 2.2 UHPLC-QTOF-MS-Based Metabolic Fingerprinting Analysis

**Figure 3:**
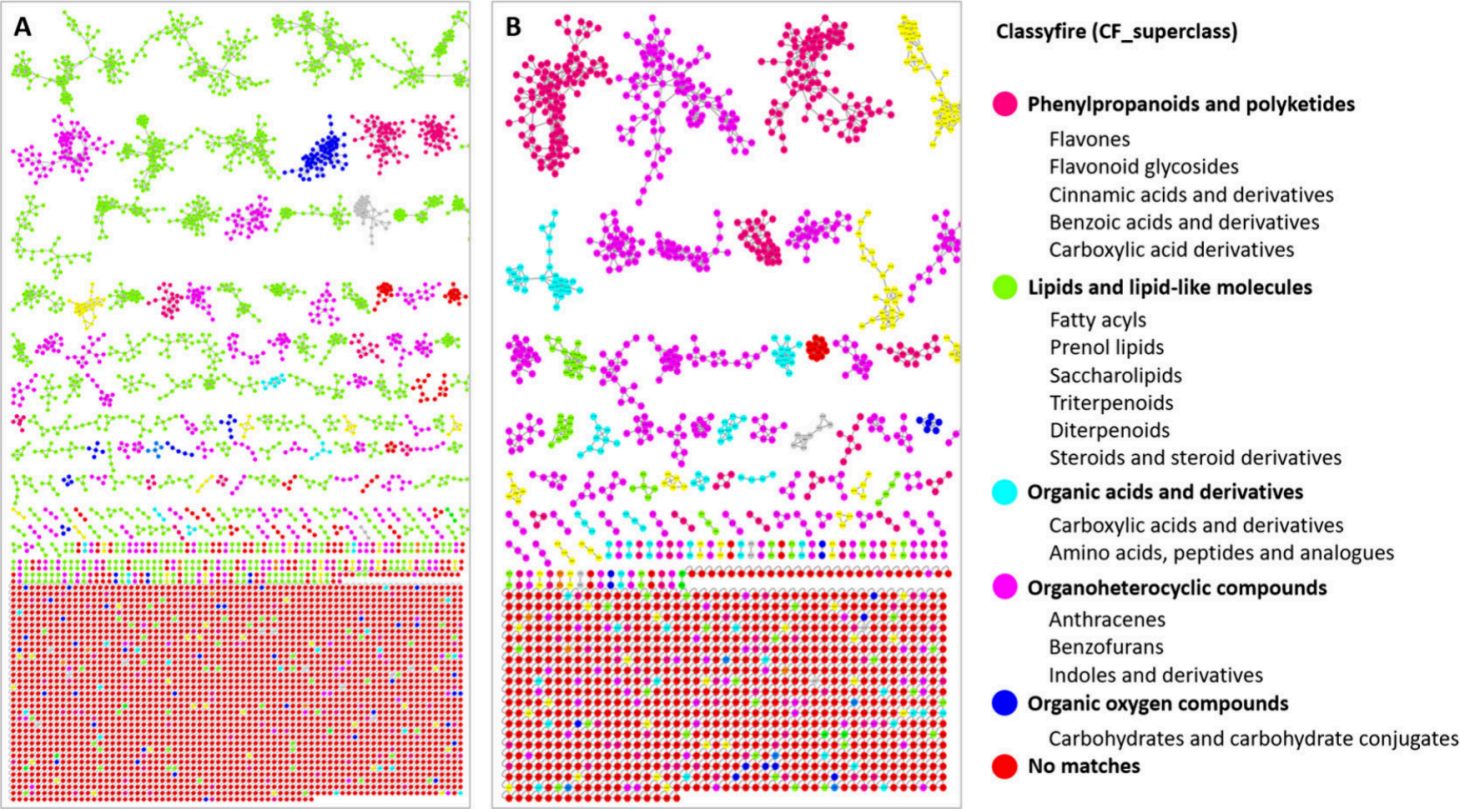
Representative MolNetEnhancer mass spectral molecular networking of consortium showing the chemical CF_superclasses in (A) negative and (B) positive electrospray ionization modes of BLEs based on UHPLC-QTOF-MS. This annotated GNPS library matches with network annotation (NAP) and DEREPLICATOR outputs.

A comprehensive metabolic fingerprinting was conducted on the BLEs using UHPLC-QTOF-MS in both negative and positive modes. Initially with the MS^2^ data, an analysis was conducted on the GNPS platform to visualize the chemical composition of the global metabolome of BLEs. The comprehensive analysis in Figure 3A and B highlight the presence of molecular families (MFs), mainly corresponding to phenylpropanoids and polyketides, lipids and lipid-like molecules, organic acids and derivatives, organoheterocyclic compounds, and organic oxygen compounds for both ionization modes. Notably, in positive ionization mode, the MFs of phenylpropanoids and polyketides, including flavonoids and derivatives of cinnamic, benzoic, and carboxylic acids, are most abundant. In contrast, in the negative ionization mode, the MFs of lipids and related molecules predominate to a greater extent. However, the prevalence of phenylpropanoid and polyketide MFs also stands out.

The structures of the compounds representing the major detected peaks are discussed below. The identification of the detected compounds was based on a search for the main molecular ions (MS) and on some of the informative fragmentations observed (MS^2^), along with the use of standards when available, retention data, UV absorption, comparison with literature data, the GNPS web platform, and public databases.

**Figure 4:**
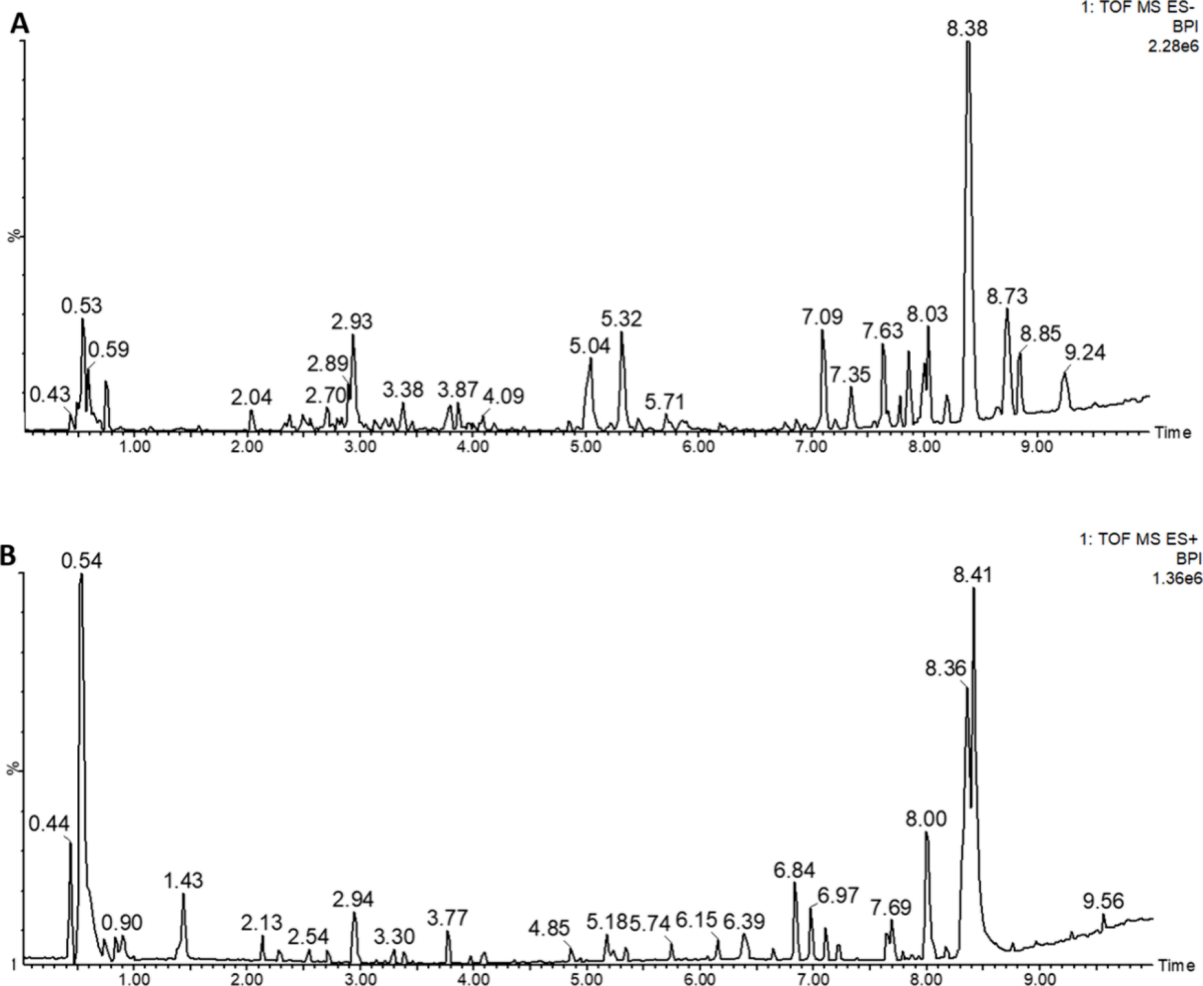
Representative UHPLC-QTOF-MS base peak intensity chromatograms of the QC samples of the BLEs in (A) negative and (B) positive ions. The retention times of the compounds that could be annotated in the BLEs are listed in Table S1.

In the present work, 49 phytochemical compounds (**2**, **3**, **8**, and **15**–**60**) have been tentatively characterized in the BLEs. Notably, there is the presence of 40 flavonoids and 9 cinnamic acid derivatives, listed in Supporting Information Table S1 and represented with the retention time in the base peak intensity (BPI) chromatograms of the quality control (QC) samples of the extracts, presented in Figure 4A and B for the negative and positive ionization modes, respectively. Importantly, many of these compounds are being reported for the first time within the genus, marking novel discoveries. Furthermore, certain compounds have been previously identified in other species of the subfamily Bambusoideae, as described in Supporting Information Table S1. The structures of the tentatively characterized compounds are presented in Supporting Information Figure S2, where the MS^2^ spectrum of several of them were determined based on the data obtained from UHPLC-QTOF-MS analysis of the studied extract, as illustrated in Supporting Information Figure S3. An extensive study of some of the compounds characterized according to their families is described below.

#### 2.2.1 Flavonoids

We report the identification of 40 flavonoids in several of the BLEs. Of the identified scaffolds, five groups were found corresponding to 12 with apigenin skeleton (**23**, **25**, **29**, **30**, **31**, **32**, **34**, **35**, **42**, **43**, **48**, and **54**), 5 with kaempferol skeleton (**36**, **37**, **38**, **57**, and **58**), 3 with luteolin skeleton (**21**, **44**, and **49**), 2 with quercetin skeleton (**39** and **40**), 2 with tricin skeleton (**51** and **60**), and 2 with naringenin skeleton (**52** and **53**). Most of them had *C*-glycosidized substituents at the C6 and C8 positions on the A-ring of the aglycone, while others had *O*-glycosidized substituents at the C7 and C3 positions of the corresponding aglycone. Flavonoids **33**, **41**, **45**, **46**, and **47** also presented *C*- and *O*-glycosidic substituents corresponding to other scaffolds, some of them with glycosidic substituents on the B-ring of the aglycone. The presence of three aglycones corresponding to one flavone **50** and 2 flavonols **55** and **59** was also identified. The presence of isoflavone **56** was also determined. Additionally, 5 compounds (**22**, **24**, **26**, **27**, and **28**) were found to be classified as unknown flavone glycosides based on their UV spectra and fragmentation patterns. These results were corroborated with the ultraviolet spectra obtained and compared with reports in literature where the range of flavones (λ_max_ ≈ 275 and 335 nm), flavonols (λ_max_ ≈ 275 and 355 nm), and flavanones (λ_max_ ≈ 275 and 340 nm) is highlighted.^32^

Compound **31** exhibited a precursor ion at *m*/*z* 563.1406 [M-H]^−^ and MS^2^ fragments at *m*/*z* 473 [M-H-90]^−^ derived from the possible loss of five water molecules, 443 [M-H-120]^−^, 383 [M-H-180]^−^, 353 [M-H-210]^−^, 325 [M-H-238]^−^, and 297 [M-H-266]^−^ derived from cross-ring cleavages of the respective sugars (hexoside and pentoside) present. Additionally, it displayed a precursor ion at *m*/*z* 565.1563 [M + H]^+^ with MS^2^ fragments at *m*/*z* 547 [M+H-18]^+^, 529 [M+H-36]^+^, 499 [M+H-66]^+^, 457 [M+H-108]^+^, 427 [M+H-138]^+^, and 379 [M+H-186]^+^. These results were consistent with those reported by Jiang et al.,^30^ and the compound was identified as isoschaftoside. This compound is a type of flavone *C*-glucoside previously reported in species such as *Phyllostachys pracecox*, *Phyllostachys edulis*, *Olyra glaberrina*, *Parodiolyra micrantha*, *Aulonemia aristulata*, *Filgueirasia arenicola*, *Filgueirasia cannavieira*, and *Merostachys pluriflora*.^32^

Compound **48** was identified as Apigenin 6-*C*-hexoside 7-*O*-hexoside with *m*/*z* = 593.1511 [M-H]^−^. The MS^2^ fragment at *m*/*z* 473 [M-H-120]^−^ indicates a typical loss of *C*-linked hexose, and the base peak at *m*/*z* 431 [M-H-162]^−^ indicates the loss of a hexose, suggesting *O*-glycosylation. These fragments confirm the presence of two hexose units, signifying an apigenin scaffold. Furthermore, fragments at *m*/*z* 353, 341, 311, and 283 resulting from cross-ring cleavages of the flavonoid glycosyl moiety’s hexoside part and the loss of water molecules, respectively, contribute additional structural insights. For bamboo species, this compound has been reported for the first time. This compound is a *C*,*O*-glycosidic flavone type reported in a study conducted for leaf extracts of *Passiflora incarnata*, *Passiflora caerulea*, and *Passiflora alata* species. In this study, they demonstrated the potent cytotoxic effect of *P. alata* and *P. incarnata* extracts against human acute lymphoblastic leukemia CCRF-CEM, mainly attributed to the content of phenolic compounds, specifically to the high content of flavones *C*-glycosides with scaffold related to apigenin and luteolin.^33^ According to the flavonoids identified and because the majority correspond to flavone *C*-glycosides, Supporting Information Table S1 details the corresponding MS^2^ fragment ions that for most of the molecules present the occurrence of the characteristic ions, e.g., [M-H-150]^−^, [M-H-120]^−^, [M-H-90]^−^, [M-H-66]^−^, [M-H-60]^−^, and [M-H-36]^−^, corresponding to the cross-ring cleavages of the hexoside and pentoside part of the flavonoid glycosyl moiety and characteristics of *C*-glycosides.

Compound **54** possesses a precursor ion *m*/*z* 269.0455 [M-H]^−^ with MS^2^ fragments *m*/*z* 151 [M-H-188]^−^, 117 [M-H-152]^−^, and 107 [M-H-151-CO_2_]^−^ corresponding to retro-Diels–Alder (RDA) cleavage accompanied by loss of CO_2_. These results were consistent with those reported by Troalen et al.,^34^ and the compound was identified as apigenin. The quercetin scaffold derived from flavone *C*-glucosides presented the product ion *m*/*z* 301 [M-H]^−^ mainly presents five MS^2^ fragment ions with *m*/*z* 273 [M-H-28]^−^, 179 [M-H-122]^−^, 151 [M-H-150]^−^, 121 [M-H-180]^−^, and 107 [M-H-194]^−^ being a product of RDA cleavage and CO and CO_2_ loss, respectively. Some quercetin derivatives have been reported for bamboo species such as *G. angustifolia*.^16,35^ Bamboo species are known to accumulate phenolic compounds, mainly glycosylated flavones, whose aglycones normally are apigenin, luteolin, and tricin. The most common flavonoids found in Asian bamboo species are the flavone *C*-glucosides.^3,6,36^

#### 2.2.2 Phenolic Acid Derivatives

Nine phenolic acids were identified in several of the BLEs. Two phenolic acids including chlorogenic acid (CGA) (**2**), caffeic acid (**3**), and *p*-coumaric acid (**8**) were identified by comparison with authentic standards. Additionally, based on literature reports, three other phenolic acid derivatives were also identified. Compounds **18**, **19**, and **20** were recognized as derivatives of caffeoylquinic acid (CQAs) and were named *O*-Feruloylquinic acid, Dicaffeoylquinic acid, and *O*-Coumaroylquinic acid, respectively. Compounds **16** and **17** were identified to the chemical class designated as not identified. Finally, compound **15** identified as quinic acid (QA) was present in all species. QA has been recently reported in *G. angustifolia* as being one of the metabolites that showed a significant increase at high altitudes in these species.^16^

Compound **2** presented the precursor ion M-H at *m*/*z* 353.0531 [M-H]^−^ and showed MS^2^ fragments at *m*/*z* 191 [M-H-162]^−^, formed by the cleavage of the ester bond between the quinic acid and caffeic acid residues, and the ion *m*/*z* 179 [M-H-174]^−^ which loses a portion of carbon dioxide to produce the ion *m*/*z* 135 [M-H-44]^−^. This report is consistent with the identification based on the MS^2^ spectrum (Supporting Information Figure S3) of the CGA pattern, together with that reported by Reis Luz et al.^37^ Although CGA is a new compound reported for several species in this study, it has been found in other bamboo species such as *Sasa argenteastriatus*, *Phyllostachys nigra* var. *henonis*, and *P. pubescens*.^38−40^

### 2.3 NMR-Based Metabolomic Fingerprinting Analysis

**Figure 5:**
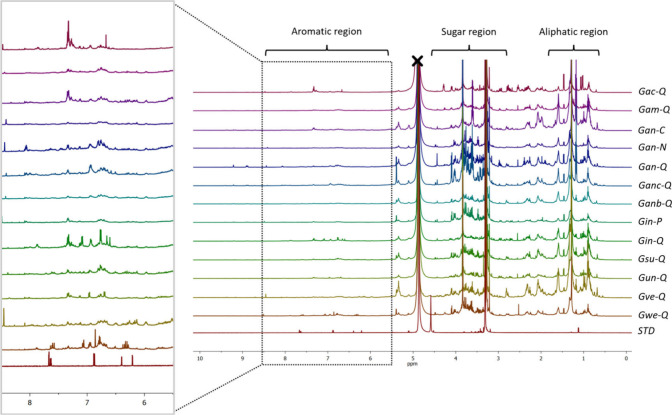
Stacked representative ^1^H-NMR spectra of the BLEs are presented. The panels display the partial assignment of aliphatic (δ_H_ 0.90–1.50 ppm), sugar (δ_H_ 3.00–5.50 ppm), and aromatic (δ_H_ 6.50–8.55 ppm) regions, respectively. An expanded spectral region from δ_H_ 5.50 to 8.50 ppm is shown for aromatic compounds. Assignments were established by extracting signals with reference standards (Quercetin and Rutin) and based on literature reports on the NMR spectra.

Representative ^1^H-NMR spectra obtained from BLEs are shown in Figure 5. The analysis of the ^1^H-NMR spectral data set led to the identification of representative metabolite signals in the samples. Fingerprinting analysis revealed that BLEs mainly contained compounds related to three specific regions, corresponding to aliphatic compounds, glycosidized compounds, and aromatic compounds. Signals in the region between δ_H_ 0.90 and 1.50 ppm were assigned to aliphatic compounds, specifically fatty acids and related molecules, determined by methyl (−CH_3_) and methylene (−CH_2_) proton signals, as confirmed by the allylic region of the aliphatic chains between δ_H_ 2.29 and 2.75 ppm.

The signals between δ_H_ 3.00 and 5.50 ppm assigned to glucoside compounds were primarily determined by the anomeric signals of the sugar compounds. Mainly, the anomeric protons of glucose in the β position with δ_H_ 4.58 (d, *J* = 7.6 Hz) and in the α position with δ_H_ 5.15 (d, *J* = 3.5 Hz) were extracted and assigned. According to our results, this sugar is present in most of the flavonoids previously found and described in Supporting Information Figure S2, confirming the respective structure annotations. It is important to note that, due to the nature of an extract, many of the signals overlap in the spectrum. However, some of the signals extracted in this study were confirmed and compared with available reference compounds. The ^1^H-NMR spectra in the aromatic region between δ_H_ 6.85 and 8.05 ppm revealed the presence of phenolic compounds, primarily related to flavonoids and phenolic acids. The presence of aromatic protons at δ_H_ 6.26 (d, *J* = 2.20 Hz, 1H), 6.44 (d, *J* = 2.22 Hz, 1H), 6.96 (d, *J* = 8.90 Hz, 2H), and 7.84 (d, *J* = 8.90 Hz, 2H), distinctive signals corresponding to a flavonoid-type scaffold derived from apigenin with substitutions at the C6 and C8 positions, particularly stands out. Representative ^1^H-NMR signals were assigned as described in Table 1.

### 2.4 Antioxidant Capacity of BLEs

**Figure 6:**
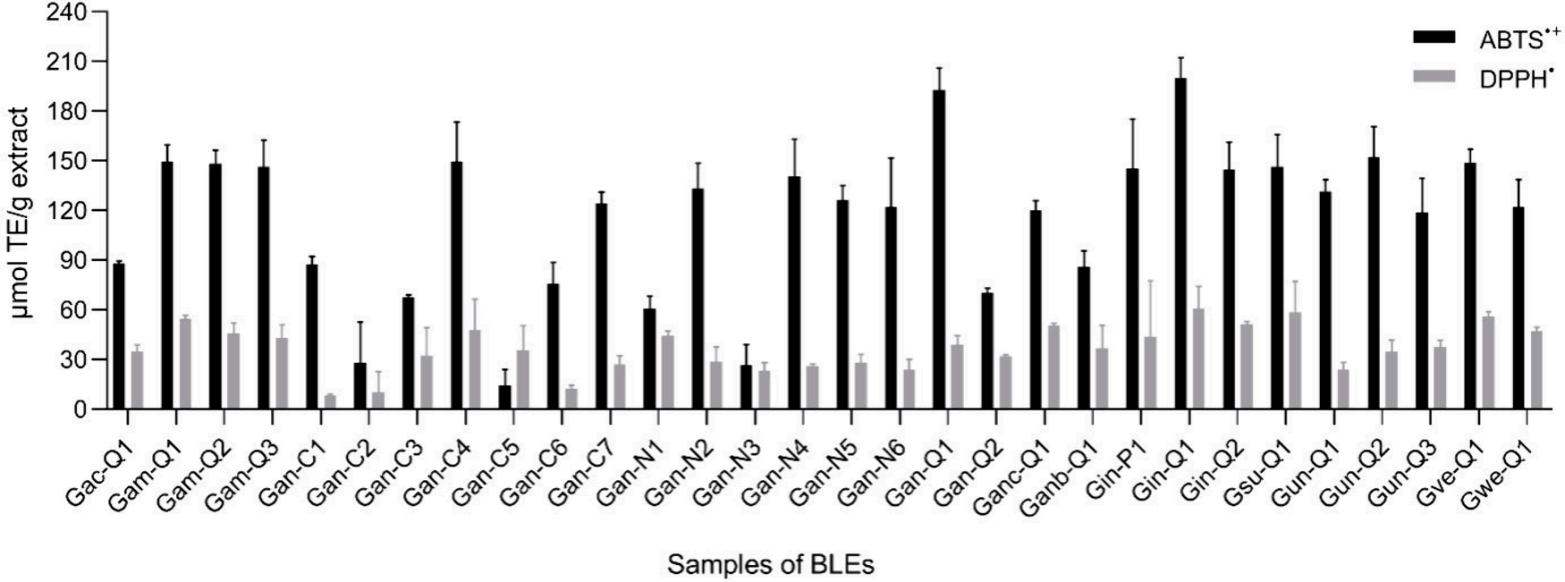
Antioxidant activity is determined for BLEs. The ABTS and DPPH of the BLEs results were expressed as μmol Trolox equivalents per gram of extract (μmol TE/g extract).

The results of the ABTS and DPPH assays are presented in Figure 6. Among the 30 BLEs tested, most exhibited significant antioxidant potential at the tested concentration of 1 mg/mL. It is evident that these extracts displayed robust antioxidant activity in both assays, which can be attributed to their high phenolic compound content. The activity was expressed as μmol of TE/g extract. Phenolic compounds constitute one of the main groups of compounds known to act as important antioxidants because they usually possess an aromatic ring that allows the stabilization and relocation of the unpaired electrons of their structure, thus facilitating the donation of hydrogen atoms and electrons from their hydroxyl groups.^41,42^ In our study, two methods were used to quantify the antioxidant capacity of 30 BLEs. When considering the antioxidant activity determined by ABTS, the species with the highest values were *G. amplexifolia* (*Gam-Q1*, *Gam-Q2*, and *Gam-Q3*), *G. angustifolia* (*Gan-C4*, *Gan-C7*, *Gan-N2*, *Gan-N4*, *Gan-N5*, *Gan-N6*, and *Gan-Q1*), *G. incana* (*Gin-P1*, *Gin-Q1*, and *Gin-Q2*), *G. uncinata* (*Gun-Q2*), and *G. venezuelae* (*Gve-Q1*), with 149.7, 148.5, 146.6, 149.7, 124.0, 133.5, 140.4, 126.4, 122.0, 192.8, 145.2, 200.2, 144.9, 152.6, and 149.1 μmol TE/g extract, respectively. Regarding the antioxidant activity determined by DPPH in each of the BLEs, the extracts with the highest antioxidant values were *G. amplexifolia* (*Gam-Q1*), *G. angustifolia* (*Gan-C4* and *Gan-N1*), *G. angustifolia* (*Ganc-Q1*), *G. incana* (*Gin-Q1*), and *G. superba* (*Gsu-Q1*), with 54.5, 48.1, 44.4, 50.7, 61.1, and 58.5 μmol TE/g extract, respectively.

### 2.5 Cytotoxic Activity of BLEs

**Figure 7:**
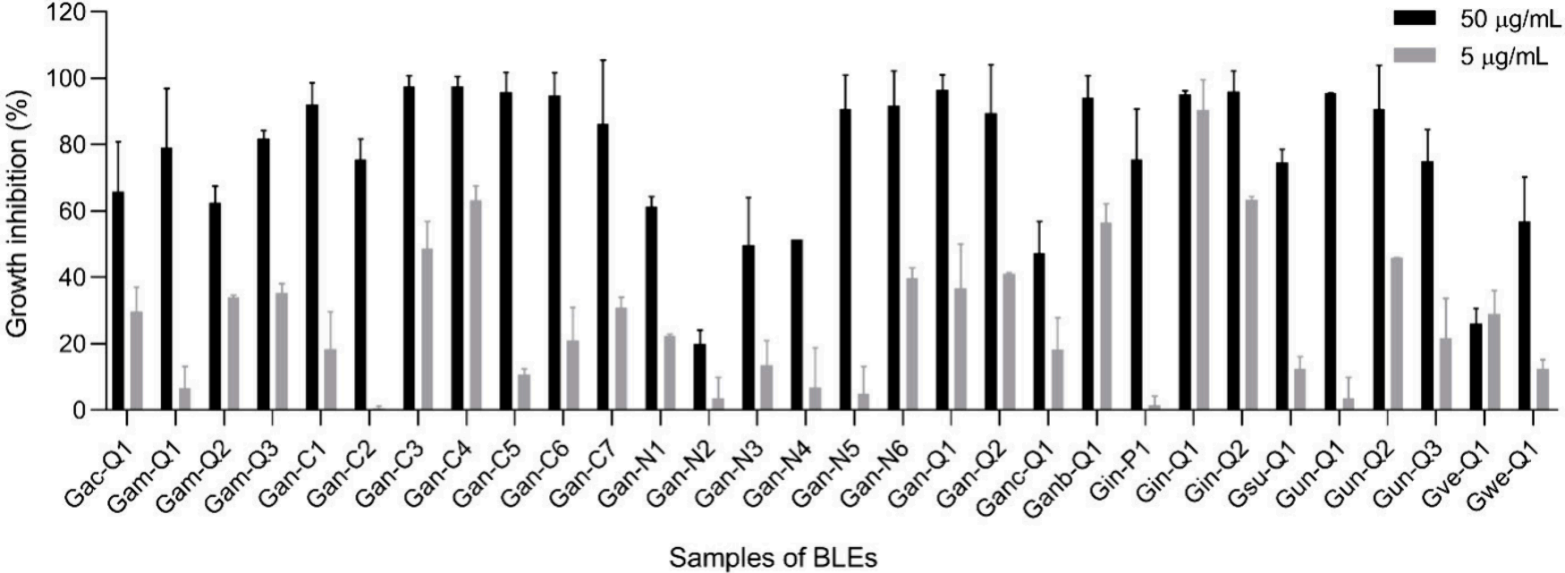
Percentage growth inhibition of BLEs evaluated at 50 and 5 μg/mL against HCT-116 (colorectal carcinoma) using MTT assay after 72 h of exposure. The percentage inhibition was calculated based on the growth of untreated cells. Data presented as mean ± SEM (standard error of the mean) from three independent duplicate experiments. All experiments utilized 0.05% DMSO (vehicle) and doxorubicin (DOXO) as negative and positive controls, respectively. Samples are considered active with an inhibition percentage higher than 85%. An extract was deemed active if it exhibited an inhibition of tumor cell lines >85% at 50 μg/mL.

To determine the cytotoxic activity, two concentrations of 5 and 50 μg/mL of the extracts were tested against HCT-116 colon cancer cells. Figure 7 shows the percentage inhibition of the exposure of each of the extracts to the cell line. Out of the 30 extracts evaluated against the cell line, it is notable that 15 of them exhibited an inhibition percentage greater than 85%, meeting the criteria for activity established according to the National Cancer Institute (NCI) guidelines at the maximum evaluated concentration (50 μg/mL).^43,44^ It should be noted that *G. angustifolia* (*Gan-C4*) and *G. incana* (*Gin-Q1*) demonstrated the highest inhibition percentages in cell growth, with values of 97.7% and 96.1%, respectively.

**Figure 8:**
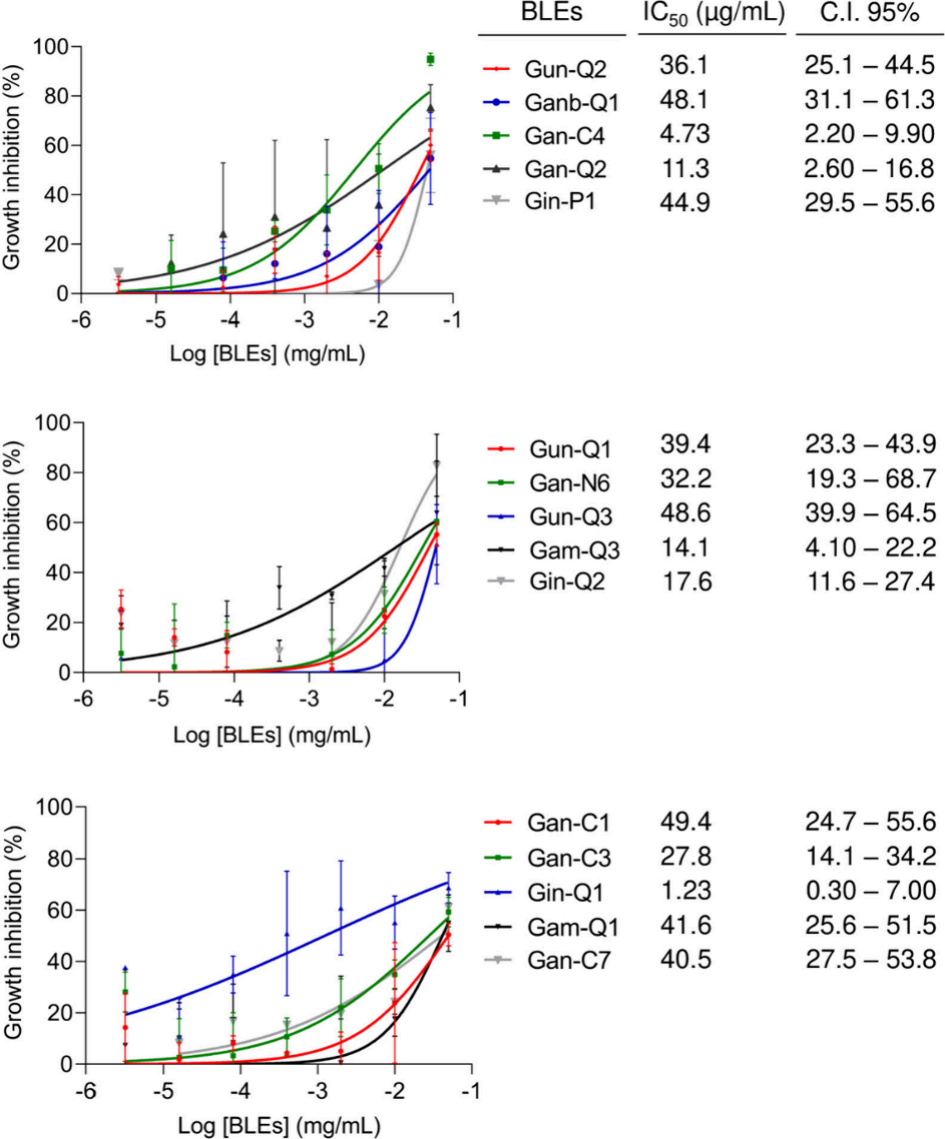
Dose–response curves of the cytotoxicity of HCT-116 cells after 72 h of treatment with the BLEs using concentrations of 3.2 × 10^–6^ to 5.0 × 10^–2^ mg/mL. The data represent the mean of three independent biological replicates with at least technical duplicates ± SEM (standard error of the mean). All experiments employed 0.05% DMSO (vehicle) and doxorubicin (DOXO) ranging from 0.00064 to 10 μM with IC_50_ = 0.2 μM (C.I 95% 0.14–0.25) as negative and positive controls, respectively.

Figure 8 displays the dose–response curves featuring the IC_50_ values of the extracts. Overall, it is evident that *G. amplexifolia*, *G. angustifolia*, and *G. angustifolia* var. *bicolor*, *G. incana*, and *G. uncinata* species exhibited a significant cytotoxic effect on HCT-116 colon cancer cell lines, with IC_50_ values below 50 μg/mL. Conversely, *G. aculeata*, *G. angustifolia* biotype *San Calixto*, *G. superba*, *G. venezuelae*, and *G. weberbaueri* did not demonstrate such an effect, displaying IC_50_ values above 50 μg/mL. According to the criteria established by the NCI, plant extracts are categorized into four groups: highly active (IC_50_ ≤ 20 μg/mL), moderately active (IC_50_ > 21–49 μg/mL), and weakly active (IC_50_ > 50 μg/mL). Cytotoxic analysis revealed that five extracts, corresponding to *G. amplexifolia* (*Gam-Q3*), *G. angustifolia* (*Gan-C4* and *Gan-Q2*), and *G. incana* (*Gin-Q1* and *Gin-Q2*), demonstrated a high cytotoxic effect with IC_50_ values of 14.1, 4.73, 11.34, 1.23, and 17.6 μg/mL, respectively. Additionally, ten extracts showed a moderate effect, including *G. amplexifolia* (*Gam-Q1*), *G. angustifolia* (*Gan-C1*, *Gan-C3*, *Gan-C7*, and *Gan-N6*), and *G. angustifolia* var. *bicolor* (*Ganb-Q1*), *G. incana* (*Gin-P1*), and *G. uncinata* (*Gun-Q1*, *Gun-Q2*, and *Gun-Q3*), with IC_50_ values of 41.6, 49.4, 27.8, 40.5, 32.2, 48.05, 44.9, 39.4, 36.1, and 48.6 μg/mL, respectively. The remaining 15 extracts exhibited a weak cytotoxic effect. This is the first report describing the cytotoxic activity of the BLEs.

### 2.6 Correlation-Based Untargeted Metabolomics Analysis

**Figure 9:**
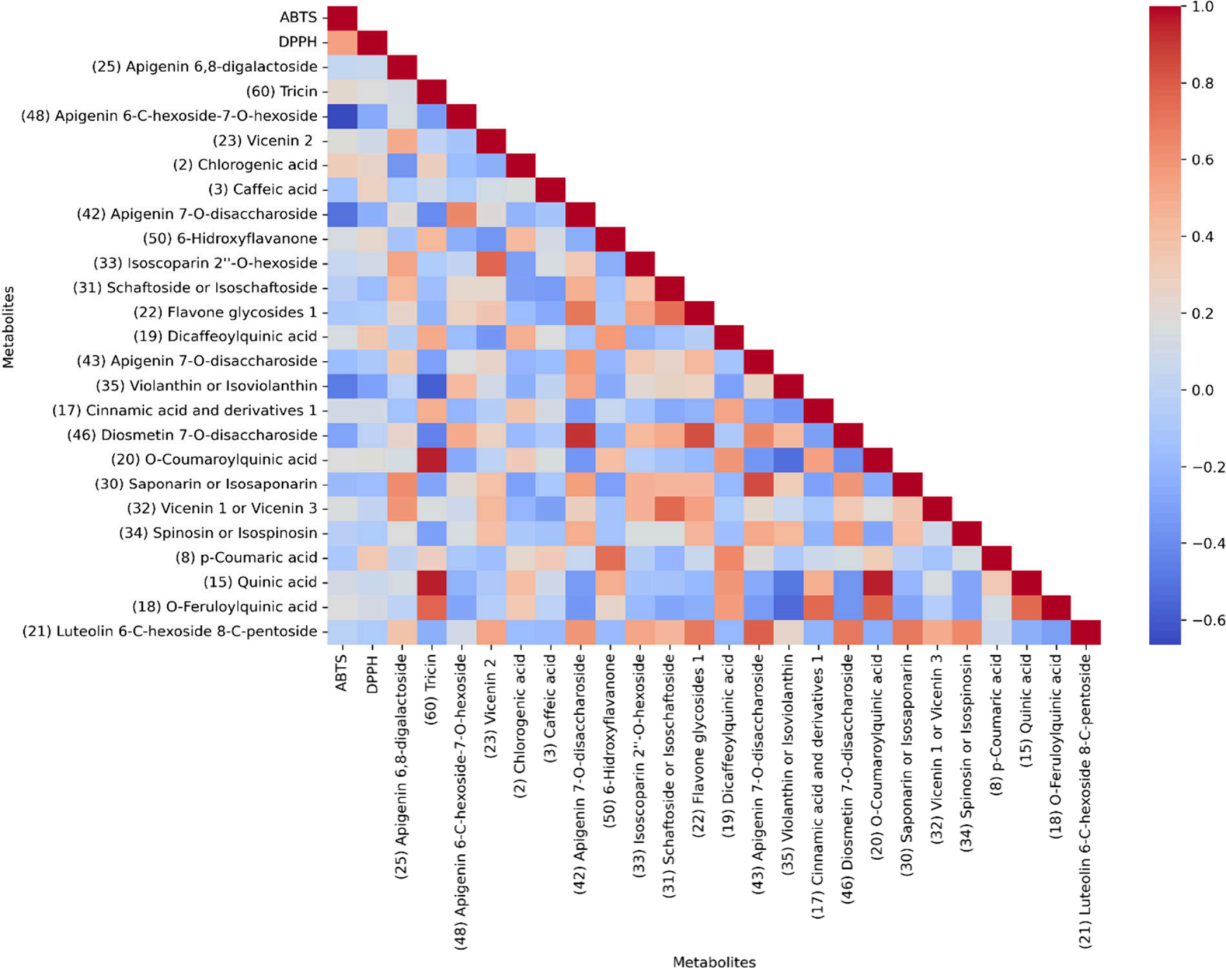
Pearson’s simple correlation coefficients for the top 24 statistically differential metabolites and antioxidant activities monocation of ABTS and DPPH of BLEs. The interpretation of the *r* values corresponds to very weak or no correlation (−0.65 to 0.00 or 0.00 to 0.25); weak correlation (0.25 to 0.50); moderate correlation (0.50 to 0.75); and strong or perfect correlation (0.75 to 1.00).

The correlation study based on the chemical composition of the extracts and antioxidant activity allowed us to determine that most of the BLEs showed a high antioxidant capacity, which was related to the high content of phenolic compounds present in these bamboos. Notably, the extracts that had a high content of flavonoids, as determined by HPTLC and HPLC-DAD analyses, were the ones that exhibited the highest antioxidant capacity, with *G. incana* and *G. angustifolia* species standing out. Figure 9 shows the relationship between antioxidant activity values and the chemical composition of the BLEs through Pearson’s simple correlation coefficients. We can observe a positive statistical relationship between the two activity variables concerning metabolites, with a correlation coefficient greater than 0.50 for most of them. We noticed a significant increase in both variables about the activity, which is closely related to the composition.

**Figure 10:**
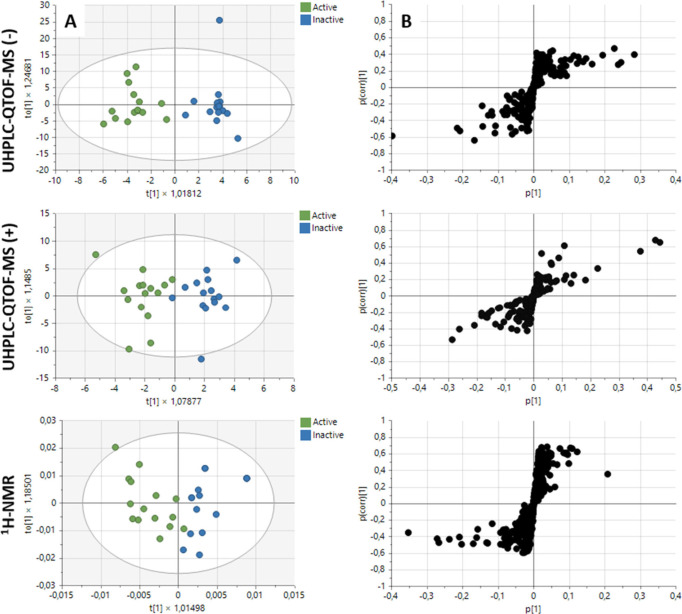
(A) OPLS-DA score plot demonstrating a distinct separation between active (green) and inactive (blue) extracts against the colon cancer cell line (HCT-116). (B) S-loading plot depicting metabolites highly correlated with HCT-116 cytotoxicity. The prediction and quality values of the OPLS-DA model for each platform used were as follows: UHPLC-QTOF-MS (−) = R^2^X(cum): 0.382; R^2^Y(cum): 0.895; Q^2^(cum): 0.226; UHPLC-QTOF-MS (+) = R^2^X(cum): 0.216; R^2^Y(cum): 0.795; Q^2^(cum): 0.578, and ^1^H-NMR = R^2^X(cum): 0.815; R^2^Y(cum): 0.680; Q^2^(cum): 0.393. The corresponding loading plot displays metabolites on the right that contributed the most to activity and on the left the metabolites that contributed the least to activity.

To identify the differential metabolites associated with cytotoxic potential, a multivariate analysis was conducted to correlate the bioactivity data with the metabolic fingerprinting obtained from UHPLC-QTOF-MS and ^1^H-NMR (Figure 10A-B). Initially, an unsupervised principal component analysis (PCA) was employed to assess the quality and stability of the instrumental platform. Supporting Information Figure S4 illustrates the clustering of the QC samples, demonstrating the reproducibility of the analysis. For a detailed investigation of the correlation between bioactivity and metabolites, a supervised orthogonal partial squares discriminant analysis (OPLS-DA) was applied, allowing for discrimination of activity variability among samples. Figure 10A represents the OPLS-DA plots for the UHPLC-QTOF-MS data in both negative and positive modes, as well as the ^1^H-NMR data. Notably, these plots highlight the discrimination between active and inactive sample groups within the specified activity range (>85% at 50 μg/mL with IC_50_ < 50 μg/mL). The established supervised models were validated through a permutation test, which assesses the quality of fit and predictive capability. In the case of cytotoxic activity against the colon cancer cell line (HCT-116), the R^2^X_(cum)_, R^2^Y_(cum)_, and Q^2^_(cum)_ values obtained from OPLS-DA analysis were deemed acceptable, demonstrating excellent reproducibility and predictability on the analytical platforms used.

The variable importance in projection (VIP) plot was generated to calculate the influence intensity and interpretability of each metabolite’s expression mode in distinguishing and classifying sample groups. In this case, this analysis facilitated the prediction of metabolites responsible for the activity based on statistically significant changes between active and inactive sample groups. The s-plot is presented in Figure 10B, highlighting variables with a VIP score >1.5, which serves as the prediction threshold. Additionally, we can observe regions where samples share a group of metabolites that contributed to cytotoxic activity to a lesser extent, and another region with metabolites contributing more significantly to cytotoxic activity. According to the above and from the OPLS-DA analysis, 27 differential metabolites were found between the active vs inactive BLEs, as summarized in Table 1. These findings are linked to the MFs identified in the analysis conducted by MolNetEnhancer. Of the metabolites that correlated with the cytotoxic potential, it was found that flavone glycosides were the ones that statistically contributed to the bioactivity in the platforms used. In the case of UHPLC-QTOF-MS, it was evident that the noted flavonoids were those that correlated positively with cytotoxicity, while for ^1^H-NMR, the signals present in the aromatic and sugar regions (δ_H_ 6.50–8.50 ppm and δ_H_ 3.00–5.50 ppm, respectively in the identified buckets) showed a positive correlation.

**Table 1:** Significantly Differential Major Metabolites Correlating with Cytotoxic Activity Determined by the OPLS-DA Model for UHPLC-QTOF-MS and ^1^H-NMR Analysis^a^

Compound number	RT (min)	δ_H_ (ppm)	Multiplicity, *J* in Hz	Type of hydrogen	CV^b^ for QC (%)	FC^c^	VIP^d^	*p*-value^e^
**2**^**st**^	2.46	7.05	d, *J* = 2.0 Hz	Quinic acid derivatives aromatic, Ar–H	7.25	0.35 **↓**	1.86	3.67 × 10^–02^
		6.82	d, *J* = 7.6 Hz					
**3**^**st**^	2.74	7.10	d, *J* = 2.0 Hz	Phenolic acid aromatic, Ar–H	1.97	0.52 **↓**	1.67	3.67 × 10^–02^
		6.75	d, *J* = 7.6 Hz					
**15**	0.57	4.80	s	Quinic acid derivatives α to oxygen, −CH–OH	10.31	0.30 **↓**	1.82	1.14 × 10^–02^
**16**	1.20				3.60	0.44 **↓**	1.82	2.09 × 10^–02^
**17**	1.94				9.34	0.53 **↓**	1.65	4.53 × 10^–02^
**18**	1.98	5.05	s	Quinic acid derivatives α to oxygen, −CH–OH	17.46	0.35 **↓**	1.60	1.61 × 10^–02^
**19**	2.04	7.12	d, *J* = 1.4 Hz	Quinic acid derivatives aromatic, Ar–H	3.40	0.35 **↓**	2.00	4.53 × 10^–02^
		6.84	dd, *J* = 7.5, 2.0 Hz					
**20**	2.33	5.05	s	Quinic acid derivatives α to oxygen, −CH–OH	17.79	0.42 **↓**	1.75	2.64 × 10^–02^
**21**	2.36	7.47	d, *J* = 2.0 Hz	Luteolin derivatives aromatic Ar–H b-ring	4.81	2.40 **↑**	1.72	1.13 × 10^–02^
		6.92	d, *J* = 7.5 Hz					
		6.60	s	Proton c-ring				
**22**	2.51				1.26	4.98 **↑**	1.73	1.28 × 10^–02^
**23**	2.68	7.84	m	Apigenin derivatives aromatic Ar–H b-ring	4.50	2.44 **↑**	1.41	1.45 × 10^–02^
		6.96	m					
		6.68	s	Proton c-ring				
**25**	2.71	7.83	m	Apigenin derivatives aromatic Ar–H b-ring	2.99	3.14 **↑**	1.47	1.45 × 10^–02^
		6.95	m					
		6.68	s	Proton c-ring				
**29**	2.87	7.83	m	Apigenin derivatives aromatic Ar–H b-ring	4.37	5.13 **↑**	1.50	4.08 × 10^–02^
		6.95	m					
		6.68	s	Proton c-ring				
		6.28	s	Proton a-ring				
30*	2.88	7.80	m	Apigenin derivatives aromatic Ar–H b-ring	6.32	3.38 **↑**	1.79	1.61 × 10^–02^
		6.90	m					
		6.50	s	Proton c-ring				
31*	2.91	7.83	m	Apigenin derivatives aromatic Ar–H b-ring	12.60	4.70 **↑**	2.26	4.27 × 10^–03^
		6.95	m					
		6.68	s	Proton c-ring				
32*	3.13				8.89	6.73 **↑**	1.55	2.64 × 10^–02^
**33**	3.16				13.65	3.52 **↑**	1.41	6.18 × 10^–03^
34*	3.18	7.87	m	Apigenin derivatives aromatic Ar–H b-ring	2.07	2.02 **↑**	1.03	2.95 × 10^–02^
		6.98	m					
		6.75	s	Proton a-ring				
		6.65	s	Proton c-ring				
**8**^**st**^	3.20	7.49	m	Phenolic acid aromatic, Ar–H *trans* vinylic protons	16.21	0.02 **↓**	1.71	9.43 × 10^–03^
		6.81	m					
		6.39	d, *J* = 15.2 Hz					
35*	3.23	7.85	m	Apigenin derivatives aromatic Ar–H b-ring	8.99	1.72 **↑**	1.25	1.13 × 10^–02^
		6.92	m					
		6.55	s	Proton c-ring				
**36**	3.24	6.96	m	Kaempferol derivatives aromatic Ar–H b-ring and a-ring	13.97	2.61 **↑**	1.34	2.48 × 10^–02^
		6.73	d, *J* = 1.6 Hz					
		6.39	d, *J* = 1.6 Hz					
**38**	3.28				9.34	2.51 **↑**	1.00	4.53 × 10^–02^
**42**	3.54				10.33	2.14 **↑**	1.34	1.86 × 10^–02^
**43**	3.56				4.94	1.96 **↑**	1.08	3.67 × 10^–02^
**46**	3.61				6.39	1.36 **↑**	1.68	8.64 × 10^–03^
**48**	3.73	7.85	m	Apigenin derivatives aromatic Ar–H b-ring	15.01	1.35 **↑**	1.78	2.64 × 10^–02^
		6.95	m					
		6.70	s	Proton a-ring				
		6.60	s	Proton c-ring				
**50**	4.57				1.07	0.28 **↓**	1.78	2.09 × 10^–02^

a Direction of comparison: Active/Inactive. Statistical parameters: CV threshold <20% for QC; FC threshold >1 (increased activity **↑**) and FC threshold <1 (decreased activity **↓**); VIP threshold >1 and *p*-value threshold <0.05.

b CV: coefficient of variation in the metabolites in the QC samples.

c FC: fold change in the comparison (average in active samples/inactive samples).

d VIP: variable importance in projection.

e *p*-Value corresponding to the *p*-values calculated by the Benjamini-Hochberg (<0.05); ^st^ The identification of compounds has been verified by using authentic standards; * Isomers. The assignments of the ^1^H-NMR signals were established by extracting signals with reference standards (Quercetin and Rutin) and based on literature reports.

**Figure 11:**
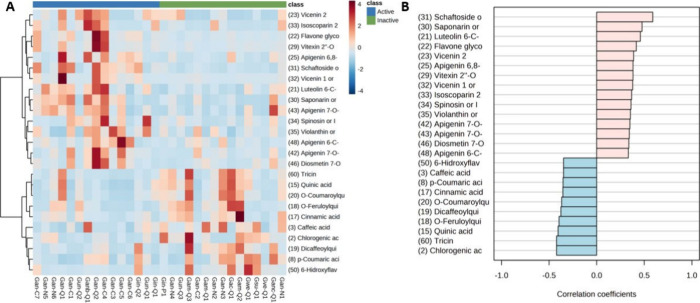
(A) Hierarchical clustering analysis (HCA) with heatmap illustrating the difference in the compounds annotated abundance between active and inactive samples. The *x*-axis shows the clustering of all the samples, and the *y*-axis shows the clustering of the 25 compounds significantly statistically. (B) Pattern search for correlation analysis of metabolites correlated with cytotoxic activity. Compounds annotated significantly statistically by UHPLC-QTOF-MS from BLEs comparing Active/Inactive groups (*p*-value threshold <0.05 and FC threshold >2).

The heatmap shows the abundance of the main statistically significant metabolites correlated with cytotoxic activity (Figure 11A). Hierarchical clustering analysis clearly reveals two clusters showing the differential chemical classes of flavonoid glycosides and caffeoylquinic acid (CQAs) derivatives with respect to activity groups. In terms of abundance, it is observed that the flavonoid glycosides are present in the BLEs that presented a higher cytotoxic effect against the colon cancer cell line HCT-116, i.e., *G. incana* and *G angustifolia* species, respectively. Confirming these findings, we constructed a graph to search for patterns of metabolites correlated with cytotoxic activity (Figure 11B). It is observed that the group of flavonoid glycosides presented positive correlation coefficients, while the CQAs did not. Taken together, these data demonstrate that BLEs are good sources of new antioxidant and cytotoxic compounds.

### 2.7 Bioactivity Score-Based Molecular Networking

**Figure 12:**
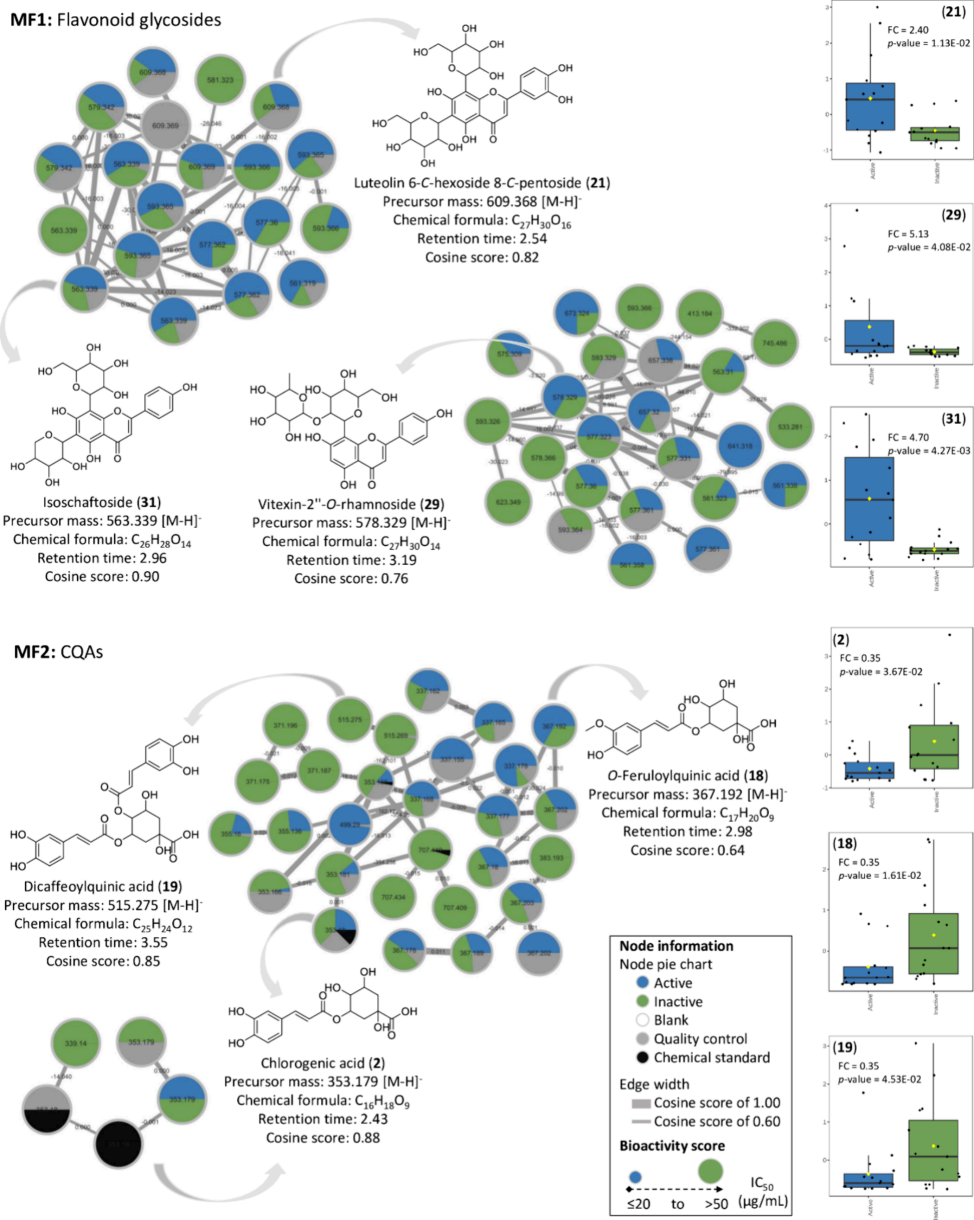
Bioactive molecular networking of the BLEs displays compounds that contribute most significantly to the activity. Particularly, flavonoid glycosides are highlighted with a high bioactivity score, while CQAs present a low bioactivity score against the colon cancer cell line HCT-116. The nodes were colored based on the classification of active and inactive groups, considering the IC_50_ value as a reference for the bioactivity score. Compounds **2**, **18**, **19**, **21**, **29**, and **31**, represented by the nodes, are the most representative according to the Pearson’s simple correlation coefficients determined by statistical analysis.

To complement the correlation analysis, the effectiveness of the molecular networking (MN) was evaluated together with the bioactivity variable of the extracts by using the GNPS web platform. The MN containing the classification component of activity is represented in Figure 12. The constructed molecular networks show the two main molecular families (MFs) present in the BLEs. On one hand, the first molecular family (MF1) corresponds to flavonoid glycosides where three metabolites corresponding to Luteolin 6-*C*-hexoside 8-*C*-pentoside (**21**, FC = 2.40; *p*-value = 1.13 × 10^–02^), Vitexin-2″-*O*-rhamnoside (**29**, FC = 5.13; *p*-value = 4.08 × 10^–02^), and Schaftoside or Isoschaftoside (**31**, FC = 4.70; *p*-value = 4.27 × 10^–03^) are represented correlating positively with cytotoxicity, and the second molecular family (MF2) corresponds to CQAs including CGA (**2**, FC = 0.35; *p*-value = 3.67 × 10^–02^), *O*-Feruloylquinic acid (**18**, FC = 0.35; *p*-value = 1.61 × 10^–02^), and Dicaffeoylquinic acid (**19**, FC = 0.35; *p*-value = 4.53 × 10^–02^), which in this case did not exhibit a correlation with cytotoxic activity against the colon cancer cell line HCT-116.

## 3 Discussion

In recent years, bamboo has found diverse applications, primarily in construction, agriculture, food production, and the manufacturing of paper and furniture.^45−47^ Despite its versatile utility, there is a notable paucity of comprehensive chemical and biological information about *Guadua* species. Specifically, the lack of studies employing metabolomic approaches for the identification of potential bioactive markers, correlating chemical composition and biological activity, hinders a deep understanding of the potential of these plants. Preliminary phytochemical investigations have provided insights into the chemical profile of *Guadua* species, revealing primarily the presence of flavonoid compounds and phenolic acid derivatives, etc.^12,48^ The studies reported to date provide a basis for comparing and confirming our findings regarding chemical composition and biological activity. Previously, several flavonoid compounds were identified in polar extracts of *G. angustifolia* leaves, including Quercetin, Kaempferol, Violanthin, and Kaempferol-7-*O*-neohesperidoside.^35^ Additional studies explored vinegar obtained from *G. angustifolia*, emphasizing its antioxidant and antimicrobial activity against *Pseudomonas aeruginosa* and *Staphylococcus aureus*, attributed to the high content of phenolic compounds.^13,49^ Another study focused on the essential oil from *G. angustifolia* and *G. chacoensis* (Rojas Acosta) Londoño and P.M. Peterson. The researchers analyzed the chemical composition and assessed antimicrobial activity against *Aspergillus brasiliensis*, *Candida albicans*, *Escherichia coli*, *P. aeruginosa*, and *S. aureus*. The majority of the essential oil composition in both essential oils primarily consisted of terpenes and compounds related to terpenes. In the essential oil of *G. angustifolia*, the main compounds were hexahydrofarnesyl acetone (23.1%) and (Z)-phytol (21.3%), while for *G. chacoensis*, the main compounds included (E)-β-ionone (8.8%), hexadecanoic acid (6.8%), hexadecenoic acid (6.5%), (Z)-phytol (5.3%), and (E)-α-ionone (5.0%). Some of these essential oils exhibited significant activity, with a MIC < 100 μg/mL, suggesting their potential as candidates for the development of new antimicrobial agents.^50^ Others studies, involving *G. angustifolia* var. *bicolor*, evaluated the antioxidant and antityrosinase potential of leaf and culm extracts. The *n*-butanol fraction exhibited the highest antioxidative activity, while the dichloromethane fraction showed tyrosinase inhibition, with inhibition percentages of 86.39% and 40.66%, respectively. Finally, the metabolites responsible for the evaluated activities were tentatively identified as *p*-coumaric acid, catechin, epicatechin, quercetagetin 7-*O*-glucoside, and genistein.^51,52^ In accordance with the above, several compounds identified in this research through chemical profiling had already been reported in BLEs, demonstrating antioxidant,^48^ antimicrobial,^50^ and antityrosinase^51,52^ potential. This knowledge of the activity of these species confirms our findings. Furthermore, these results are consistent with a recent study where untargeted metabolomics was applied to determine changes in chemical composition under the influence of altitude in 111 samples of bamboo plants collected in different regions of Colombia, confirming the presence of some of the MFs reported in this study. Additionally, the study highlights the prevalence of phenylpropanoid and polyketide MFs, indicating the presence of flavonoids and cinnamic acid derivatives, mainly.^16^ These compounds, widely distributed as secondary metabolites in most plants, are important due to their ability to act as antioxidants. Many phenolic compounds have been reported to possess potent antioxidant activity, which may also be associated with anticancer, antibacterial, antiviral, or anti-inflammatory properties to varying degrees.^53−55^

The findings of this work demonstrate that BLEs have antioxidant potential, which aligns with data collected for certain species of bamboo leaves such as *G*. *angustifolia*.^14^ In their study, different extraction methods were employed with solvents of varying polarity, including ethyl acetate, diethyl ether, and 50% ethanol, using three separate extraction techniques: reflux, Soxhlet, and ultrasound. Additionally, they evaluated the antioxidant capacity using the DPPH methodology in their extracts at a concentration of 50 mg/mL. It was highlighted that the extracts of medium and high polarity, corresponding to ethyl acetate and 50% ethanol, presented the highest antioxidant activity. This observation could indicate the presence of polyphenolic compounds, such as flavonoids, to which antioxidant activity has been attributed and that have been found in bamboo leaves, specifically *Phyllostachys nigra* var. *henonis*.^56,57^ It is important to highlight that in our study we used a concentration of 1 mg/mL to evaluate antioxidant activity through the ABTS and DPPH assays. Therefore, when compared with the previous study, our results for antioxidant activity indicate that most of the BLEs presented a substantial antioxidant potential, with values greater than 100 and 30 μmol of TE/g of extract for ABTS and DPPH, respectively. Another study assessed the antioxidant potential of bamboo vinegar obtained through the pyrolysis process of *G. angustifolia*. The antioxidant activity in vinegar was determined by both methods. In this study, the concentration of syringol was quantified in the different formulations obtained from the vinegar, revealing that this compound, along with others such as guaiacol, exhibited antioxidant capacity. Furthermore, typical compounds of phenolic acids with antioxidant properties were also identified. Free radical scavenging activity of 30.9 μmol/L TEAC was observed at a concentration of 0.23%. Notably, at a vinegar concentration of 10%, the most effective capture of the DPPH radical was achieved, with a value of 940.2 μmol/L TEAC.^58^

The highest antioxidant capacity of BLEs using the DPPH method was 61.1 μmol of Trolox/g of extract. These values were lower than those obtained by Mosquera et al.^48^ in ethanolic extracts evaluated at a concentration of 1 mg/mL in *G. angustifolia*, which presented an antioxidant activity of 80 μmol Trolox/g of extract. However, for the ABTS method, the highest antioxidant capacity of the extracts was 200.2 μmol Trolox/g of extract, which was higher compared to the results obtained by Mosquera et al.,^48^ who reported an antioxidant capacity of 180 μmol Trolox/g extract by ABTS. Studies conducted by Lozano-Puentes et al.^19^ reported the antioxidant activity of several ethanol extracts from optimized leaves of *G. angustifolia* obtained through different levels of sonication (low, medium, and high) evaluated at three times (10, 20, and 30 min) and three temperatures (20 °C, 35 °C, and 50 °C), where the antioxidant capacity was determined. It was found that extraction at 50 °C for 20 min favored the extraction of phenolic compounds and total flavonoids, presenting the highest antioxidant capacity with values of 144.76 and 209.23 μmol Trolox/g of extract for the ABTS and DPPH methods, respectively. This aligns with what was found in this study, given that many of the extracts were obtained under room temperature conditions and by the percolation method and an abundant presence of flavonoids was detected, as seen in the species *G. incana* with antioxidant activity values of 200.2 and 61.1 μmol Trolox/g of extract and in *G. angustifolia* with values of 140.4 and 26.1 μmol Trolox/g for ABTS and DPPH, respectively.

Considering that only previous reports have been found for the species of the genus *Guadua*, and to strengthen our discussion, a search was carried out at the Bambusoideae subfamily level for related studies to compare our results of chemical composition and biological activity. In this sense, it was found, particularly, that the dried leaves of *Sasa veitchii* bamboo, a species traditionally used in Asian folk medicine, reveal its use as a source of antioxidant food supplements in Japan. Additionally, the Chinese Food Additives Standardization Committee approved an extract of *Phyllostachys nigra* var. *henonis* known as antioxidant of bamboo leaves (AOB) as a novel kind of natural antioxidant due to its potent antioxidant capacity attributed to phenolic compounds.^3,59^ Products derived from bamboo extracts, coupled with the increased production of reactive oxygen species (ROS), are believed to play a significant role in preventing disease development and reducing the impact of oxidative stress when disease occurs.^39,60^

We highlight that according to our results, to date, the BLEs included in this research do not present any report of cytotoxic activity, this being the first report. Despite the absence of reports on cytotoxicity in these bamboos, it is striking that, in general, other bamboo species such as *Phyllostachys heterocycla* and *Dendrocalamus asper* have shown anticancer effects against various cell lines, including HepG2, MCF-7, HeLa, A549, and THP-1, which are directly associated with the high content of polyphenols.^31,61^ For example, in a previous study, the polar fraction of *Phyllostachys heterocycla* var. *pubescens* (Pradelle) Ohwi was shown to exhibit selectivity by inhibiting the epidermal growth factor receptor (EGFR) in HepG2 cells. In this study, the isolation of 7-hydroxy-5-methoxy-methylcinnamate showed IC_50_ values of 7.43 and 10.65 μM against HepG2 and MCF-7 cell lines, respectively. Moreover, it induced apoptotic cell death in HepG2 cells, with a total apoptotic rate of 58.6% compared to 4.71% in the control, achieved by arresting cell cycle progression in the G1 phase.^9^ Previous studies in other bamboo species have reported cytotoxic activity, e.g., in the crude methanol extract obtained from the skin of the shoots of bamboo *Phyllostachys heterocycla*. The crude extract was evaluated against five cell lines-four of them tumorigenic (HepG2, HeLa, A549, and MCF-7) and one nontumorigenic cell line. The results showed that the crude extract presented a moderate cytotoxic effect against HepG2 and MCF-7, with IC_50_ values of 48.4 and 38.9 μg/mL, respectively, while against HeLa it showed a weak effect with an IC_50_ value of 50 μg/mL.^11^ In a more recent study of *P. heterocycla* var. *pubescens*, the cytotoxic effect was evaluated against the crude methanol extract and the fractions obtained from *n*-hexane, ethyl acetate, and *n*-butanol, targeting five cell lines: HepG2, HeLa, A549, MCF-7, and THP-1. It was reported that the *n*-butanol fraction exhibited inhibition percentages exceeding 60% against all cell lines at the maximum concentration of 100 μg/mL. Notably, the *n*-butanol fraction demonstrated the highest inhibition at 94.31% against the HeLa cell line.^9^ In the broader context of our research on the chemical and biological properties of *Guadua* species, our study significantly expands the existing knowledge base, particularly concerning cytotoxic activity. Although previous reports have been scarce, our results of the activity of BLEs against HCT-116 cells represent a novel contribution to the understanding of the anticancer potential of these bamboos. However, there is still much to explore about the mechanisms of action that exert their effect on cells. The absence of previous reports on cytotoxicity in these plants, as highlighted in our study, underscores the limited exploration of their therapeutic effects. However, previous studies support our findings, confirming that these extracts may be promising candidates for bioactive compounds, specifically as anticancer agents.

Our interest in HCT-116 cells and the need for further research on the antioxidant and cytotoxic potential of *Guadua* species led us to establish a correlation between the chemical composition and the antioxidant and cytotoxic activities of BLEs to identify the bioactive compounds responsible for these effects. This holistic approach not only improves our understanding of BLEs but also lays the foundation for the development of new therapeutic agents with potential applications in cancer research and treatment. Antioxidant activity is intricately linked to fundamental cellular processes such as apoptosis, proliferation, lipid metabolism, cell differentiation, and immune response. In the course of cellular metabolism, the generation of reactive oxygen species (ROS) can induce cellular damage and contribute to diseases primarily through superoxide anions, nitric oxide (NO^•^) radicals, hydroxyl radicals, and hydrogen peroxide. The oxidation of oxygen free radicals has been identified as a pivotal factor in the onset and progression of various diseases, including cardiovascular diseases, neurodegenerative disorders, atherosclerosis, tumors, etc.^62^ Based on this context and taking into account that it is the first approach in bamboos, it is highlighted that the compounds corresponding to flavonoids and derivatives of phenolic acids correlated positively with the antioxidant potential, previously highlighted in the studies described. Particularly, in a study conducted by Ni et al. on the species *Sasa argenteastriatus*, it was found that they correlated the chemical composition with antioxidant activity, determining that the content of total flavonoids (TF) and total phenols (TP) obtained from the leaves of this ornamental bamboo exhibited a high correlation with antioxidant activity. Additionally, eight characteristic compounds of this bamboo were identified, including orientin, isoorientin, vitexin, homovitexin, *p*-coumaric acid, chlorogenic acid, caffeic acid, and ferulic acid. In general, it is highlighted that seasonal variation and the content of TF and TP influence the DPPH radical scavenging capacity. The correlation coefficient between TF, TP, and DPPH radical scavenging is TF > TP, indicating that TF in bamboo leaves plays a primary role in the DPPH radical scavenging capacity. The correlation coefficient between TF, TP, and antioxidant capacity obtained by the FRAP assay is TP > TF.^38^ According to the above, our correlation results of the chemical composition and antioxidant activity align with those of the previous study. Although the total flavonoid and total phenolic contents were not determined, it was established that about 50% of the chemical composition of the BLEs corresponds to flavonoids and derivatives of phenolic acids. On the other hand, compounds reported in this work, such as vicenin 2, chlorogenic acid, caffeic acid, schaftoside or isoschaftoside, saponarin or isosaponarin, etc., presented a higher correlation coefficient >0.5, demonstrating powerful antioxidant activity, in agreement with the findings reported and compared by Ni et al. Similarly, a recent study by Ko et al. on the effect of seasonal variations on phenolic compounds and the antioxidant and anti-inflammatory activity of *S. quelpaertensis* Nakai through a correlation study demonstrated that phenolic compounds (*p*-coumaric acid, tricine, TP, and TF content) in ethanol extracts of leaves (ESL) showed an excellent correlation with antioxidant activities, indicating that ESL contained the highest levels of antioxidant and anti-inflammatory substances.^63^ However, in our study, the flavone tricine showed correlation coefficients <0.5, indicating a negative correlation with antioxidant activity, unlike the findings reported by Ko et al. This allows us to determine that the effect is possibly attributed to the synergistic effect^64,65^ of all the compounds present in the BLEs or to other specific phenolic compounds such as *p*-coumaric acid and Luteolin 6-*C*-hexoside 8-*C*-pentoside, presenting a coefficient of correlation >0.7, indicating a potent antioxidant effect for both methods.

On the other hand, for cytotoxic activity, it was found that flavonoid glycosides presented a positive correlation with this effect. These results are consistent with those reported for a wide variety of flavonoid glycosides that exhibit cytotoxic activity against various cell lines, including MCF-7, HCT-116, HepG2, HeLa, etc. Some flavones, including apigenin, baicalein, chrysin, luteolin, nobiletin, tangeretin, and wogonin, have shown cytotoxic potential against various colon cancer cell lines, such as Caco-2, COL-2, COLO201, COLO205, COLO320, HCT-115, HCT-116, HT-29, etc., with IC_50_ values <100 μM.^66^ We found that glycosated flavones particularly showed a positive relationship with increased cytotoxic activity, whereas caffeoylquinic acids did not. Although there are no studies on the effect of BLEs against HCT-116 colon cancer cell lines, the effect of extracts of bamboo shoots (*Dendrocalamus asper*, *Phyllostachys heterocycla* var. *Pubescens*, and *S. quelpaertensis*) against MCF-7 breast cancer cells and HCT-116 colon cancer cells lines has been reported, showing cytotoxic, antiproliferative, proapoptotic, and proin-flammatory effects.^7,67^ A recent study investigated the role of NO^•^ and inhibitors of apoptosis (IAPs) during the process of apoptosis induced by *S. quelpaertensis* leaf extracts (SQE) in p53 wild-type (WT) and p53 null HCT-116 colon carcinoma cells. SQE was shown to induce apoptosis independent of p53 status and was associated with modulation of endogenous NO^•^ in HCT-116 cells.^67^

Sak reported that flavonoids, such as quercetin and baicalein, are recognized as beneficial agents for preventing and treating colon cancer. However, in contrast to their glycosides, rutin and baicalin, respectively, they did not exhibit growth inhibitory effects on colon cancer cells. This observation highlights the substantial impact of the sugar fraction on the bioactivity of flavonoids.^66^ Interestingly, our correlation study contradicts this finding, especially concerning glycosylated flavones identified as vicenin 1 or vicenin 3 (**32**, FC = 6.73; *p*-value = 2.64 × 10^–02^), Vitexin 2″-*O*-rhamnoside (**29**, FC = 5.13; *p*-value = 4.08 × 10^–02^), Flavone glycosides (**22**, FC = 4.98; p-value = 1.28 × 10^–02^), Schaftoside or Isoschaftoside (**31**, FC = 4.70; *p*-value = 4.27 × 10^–03^), and Saponarin or Isosaponarin (**30**) (FC = 3.38; *p*-value = 1.61 × 10^–02^), which demonstrated significant changes and are associated with cytotoxic potential in the most active BLEs. To validate these results, isolating potential bioactive markers from the most active extract and confirming their cytotoxic activity is essential. However, a recent study by Vukovic et al. evaluated the cytotoxicity of 11 flavonoids isolated from propolis against colon cancer cell lines (HCT-116) and breast cancer cells (MDA-MB-231). Six flavonoids induced cytotoxic effects in both cell lines. Luteolin exhibited cytotoxicity, especially in HCT-116 cells, with an IC_50_ value after 72 h of exposure of 66.86 μM. Myricetin demonstrated cytotoxicity with an IC_50_ value of 114.75 μM, inducing apoptosis in MDA-MB-231 cells.^68^ The preceding data, related to the correlation study and bioactivity of glycosylated flavones against the colon cancer cell line HCT-116, align consistently with our results. Thus, a straightforward identification of potential biomarkers was achieved prior to any fractionation or isolation efforts. However, studies focused on the isolation of cytotoxic compounds are required to confirm our hypothesis.

Molecular networking analysis has enabled the identification of bioactive molecules within BLEs, whose active constituents were previously unknown against colon cancer cells HCT-116. In the study, it was emphasized that compounds **21**, **29**, and **31** exhibited a positive correlation, affirming the earlier observations. The present study demonstrated that the application of a metabolomics approach, based on correlation of chemical composition and biological activity, together with the construction of integrated molecular networks with bioactivity scores, can accelerate the search for bioactive molecules and rationalize the isolation procedure in bioassay-based phytoconstituent discovery.^69^ This innovative tool offers the possibility to narrow the pool of candidate compounds for further isolation, thus minimizing the time required in this process.

Our study faced several limitations. First, the isolation of bioactive compounds is necessary to validate the correlation study. Second, due to the limited availability of individuals of certain species during the plant collection phase, we recommend incorporating a larger number of individuals in future studies. This approach will strengthen statistical analysis and ensure a more comprehensive exploration of the metabolome in bamboos. Third, given the limited biological information available for these species, it is crucial to evaluate additional biological activities that support our findings. Finally, to elucidate the mechanisms of action on HCT-116 cells and validate their therapeutic potential, more specific biological activity assays are needed.

## 4 Conclusion

The present study offers a comprehensive view of *Guadua* species, emphasizing their chemical and biological potential. Despite incomplete information on these plants, 49 compounds, including flavonoids and phenolic acid derivatives, have been identified, showing a strong correlation with antioxidant and cytotoxic activities. Remarkably, this study is the first to report the cytotoxic activity of BLEs against the colon cancer cell line HCT-116. Our findings underscore a direct link between the abundance of phenolic compounds, such as Vicenin 2, Chlorogenic acid, and Caffeic acid, and antioxidant activity. Additionally, glycosylated flavones are positively correlated with cytotoxicity, contrasting with the negligible impact of CQAs. Furthermore, the application of a metabolomic approach and molecular network analysis has enabled the identification of bioactive molecules within BLEs, streamlining the pursuit of compounds for future isolation studies. Nevertheless, further investigations are warranted to isolate and validate these bioactive compounds as well as to better understand the mechanisms of action associated with the antioxidant and cytotoxic activities observed in these species. A detailed understanding of the bioactive compounds present in bamboo and their potential impact on human health could lead to the development of innovative therapies for various diseases as well as the formulation of cosmetic and nutraceutical products. In addition, future research could explore the role of bamboo as a natural source of antioxidants and cytotoxic agents in the prevention and treatment of cancer diseases as well as its application in the food and health industry.

## 5 Materials and Methods

### 5.1 Reagents

1,1-Diphenyl-2-picrylhydrazyl (DPPH), 2,2-azinobis(3-ethylbenzothiazoline-6-sulfonic acid) (ABTS), and 6-hydroxy-2,5,7,8-tetramethylchroman-2-carboxylic acid (Trolox, Hoffman-La Roche) were purchased from Sigma-Aldrich Co., (St. Louis, MO, USA). Methanol, acetonitrile, and formic acid HPLC grade were acquired from Merck (Darmstadt, Germany). Ultrapure water was used throughout and was produced by a Milli-Q Ultrapure water system with a resistivity of 18.2 MΩ·cm at 25 °C (Millipore, Bedford, USA). For the NMR experiments, methanol-*d*_4_ (>99.8 atom % D) and deuterium oxide (>99.8 atom % D) were purchased from Sigma-Aldrich USA (St. Louis, MO, USA). Dimethyl sulfoxide (DMSO) was purchased from Panreac AppliChem. Gallic acid (purity: > 98%, CAS No. 149–91–7), Chlorogenic acid (purity: > 98%, CAS No. 202650–88–2), Caffeic acid (purity: > 98%, CAS No. 501–16–6), Isoorientin (purity: > 98%, CAS No. 4261–42–1), Ampelopsin (purity: > 98%, CAS No. 27200–12–0), Rutin (purity: > 98%, CAS No. 153–18–4), Vitexin (purity: > 98%, CAS No. 3681–93–4), *p*-Coumaric acid (purity: > 98%, CAS No. 501–98–4), Sinapic acid (purity: > 98%, CAS No. 7362–37–0), Morin (purity: > 98%, CAS No. 480–16–0), Coumarin (purity: > 98%, CAS No. 91–64–5), Quercetin (purity: > 98%, CAS No. 117–39–5), Cinnamic acid (purity: > 98%, CAS No. 140–10–3), and Naringenin (purity: > 98%, CAS No. 480–41–1) were obtained from Sigma-Aldrich (St. Louis, MO, USA). All these compounds were used as reference standards. RPMI-1640 medium, fetal bovine serum (FBS), and penicillin–streptomycin were obtained from Gibco BRL (Grand Island, NY, USA).

### 5.2 Plant Material and Preparation of BLEs

Mature leaves obtained from the apical branches of 10 bamboo species belonging to the genus *Guadua* were collected from different locations in Colombia as described in Table 2. The voucher species were deposited in the Herbarium of Pontificia Universidad Javeriana (HPUJ) and determined by Néstor García and Ximena Londoño. The plant materials were dried for 96 h at 35 °C in an oven with forced air circulation, followed by being ground to a fine powder. The material obtained was subjected to a percolation process with ethanol (plant:solvent, 1:10 *w*/*v*) at room temperature for 24 h with solvent changes repeating four times. Subsequently, the extracted solutions obtained were concentrated on a rotary evaporator under reduced pressure at an average temperature of 35 °C, providing the corresponding BLEs and stored at room temperature until used.

**Table 2:** Information of *Guadua* Species

Species	Collection place^a^	Samples code	Voucher number (HPUJ)	Number of samples^b^
*Guadua aculeata* E. Fourn.	Quindío	*Gac-Q* (1)	HPUJ-30736	1
*Guadua amplexifolia* J. Pressl	Quindío	*Gam-Q* (1 to 3)	HPUJ-30733	3
*Guadua angustifolia* Kunth	Cundinamarca	*Gan-C* (1 to 7)	HPUJ-30740	7
	Nariño	*Gan-N* (1 to 6)	HPUJ-30722	6
	Quindío	*Gan-Q* (1 to 2)	HPUJ-30731	2
*Guadua angustifolia* Kunth biotype San Calixto	Quindío	*Ganc-Q* (1)	HPUJ-30737	1
*Guadua angustifolia* var. *bicolor* Londoño	Quindío	*Ganb-Q* (1)	HPUJ-30735	1
*Guadua incana* Londoño	Putumayo	*Gin-P* (1)	HPUJ-30723	1
	Quindío	*Gin-Q* (1 to 2)	HPUJ-30723	2
*Guadua superba* Huber	Quindío	*Gsu-Q* (1)	HPUJ-30734	1
*Guadua uncinata* Londoño and L. G. Clark	Quindío	*Gun-Q* (1 to 3)	HPUJ-30730	3
*Guadua venezuelae* Munro	Quindío	*Gve-Q* (1)	HPUJ-30729	1
*Guadua weberbaueri* Pilg.	Quindío	*Gwe-Q* (1)	HPUJ-30738	1

a Information regarding collection sites is provided in Supporting Information Table S2.

b A total of 30 BLEs were prepared.

### 5.3 *Cleanup* of the BLEs

The BLEs were solubilized in methanol:water 95:5 (*v*/*v*) and a *cleanup* step was performed for biological test and chromatographic analyses. The extracts underwent a *cleanup* step in a solid phase extraction cartridge (Strata X, C18). In this procedure, first the cartridge was packed with 6 volumes (∼5 mL) of HPLC grade methanol. Then the cartridge was equilibrated with 6 volumes of methanol:water solution at 95% and the extract obtained in the previous step was applied. The elution was carried out with 3 mL of 95% methanol:water. The extract obtained was dried by rotary evaporation and stored at room temperature until used for analysis.

### 5.4 Preparation of Chemical Standards and Quality Control (QC) Samples

Chemical standards solutions were prepared as 1 mg/mL solutions in methanol. Multiple QC samples were prepared by pooling and mixing equal volumes of each extracted sample to check the system performance and reproducibility in sample analysis. To assess the robustness of the instrument, pooled QC samples were injected prior to the sample analysis until system equilibration was achieved and after every ten randomized sample injections.

### 5.5 HTPLC Data Acquisition

The standard solutions were prepared by dissolving 1 mg of Quercetin and Rutin in 1 mL of methanol (1000 μg/mL), and the extracts were prepared by dissolving 10 mg in 1 mL of ethanol (10000 μg/mL) to seed 10 μL in each band of the plate and obtain a final amount of 10 and 100 μg, respectively. Each extract was filtered through a 0.22 mm polypropylene membrane filter for subsequent HPTLC analysis. Next, all samples were examined on HPTLC equipment (Camag, Muttenz, Switzerland), which included an autosampler (ATS 4), a developer (ADC), a derivatizer (DV), a development chamber, a visualization chamber (Visualizer 2, CAMAG), and VisionCATS software version 3.1.21109.3. HPTLC was performed on the Silica gel 60 F_254_ precoated TLC plates of 10 × 10 cm^2^ (Merck, Darmstadt, Germany). Ten microliter sampless of the extracts were applied to the plates in the form of 7 mm bands with the Camag automatic applicator equipped with a 10 mL syringe and operated with the following settings: band length 7 mm, application rate 10 s/mL, distance between 6 mm, distance from the lateral edge of the plate 2 cm, and distance from the bottom of the plate 2 cm. For each analysis, the factors that were kept constant were developing distance: 75 mm, developing time: 10 min, detection reagent: natural product reagent (1 g of 2-aminoethyl diphenyl borate in 100 mL of ethanol) (NP/PEG), postchromatographic derivatization time: 5 min. Plates were eluted with *n*-hexane/ethyl acetate/formic acid 10/6/1 (*v*/*v*/*v*) for flavonoid aglycones and ethyl acetate/formic acid/acetic acid/water 100/11/11/27 (**v**/**v**/**v**) for flavonoid glycosides in a chamber (20 cm × 10 cm, Camag) followed by air drying. For derivatization, the following reagents were applied by piezoelectric spraying. After development and derivatization of the bands, the plates were scanned at a wavelength of 365 nm by using the visualization chamber. The VisionCATS software controlled all of the modules of the equipment. The bands of the reference standards (Quercetin and Rutin) had their *R*_*f*_ measured for comparison with the bands of the extracts.

### 5.6 HPLC-DAD Data Acquisition

Samples were prepared by weighing 5 mg of each extract and solubilizing them with 1 mL of methanol. The chromatograph used was a Shimadzu UFLC system (Shimadzu, Duisburg, Germany) coupled to a diode array detector (DAD, VIS-Shimadzu 1520 PC-Diode Array), a pump model 6-AD Shimadzu SCL-10VP control system, an in-line degasser, and an autosampler. Chromatographic separation was performed on a Luna C18 column (150 × 4.6 mm^2^, 5 μm, 100 Å) was used to carry on the chromatographic separation at 30 °C. The mobile phase used was eluent A (Water 0.1% formic acid, *v*/*v*) and eluent B (Acetonitrile 0.1% formic acid, *v*/*v*) with flow of 1 mL/min. Gradient elution was performed as follows: 0 to 40 min, 5% to 100% B; 40 to 50 min, 100% B; 50 to 53 min, 100% to 5% B; and 53 to 63 min, 5% B. The wavelengths used were 274 and 365 nm, with spectra acquired in a range of 200 to 400 nm. Dereplication studies were performed to compare retention times and UV spectrum.

### 5.7 UHPLC-QTOF-MS Data Acquisition

For UHPLC-QTOF-MS analysis, the BLEs were dissolved in methanol at a concentration of 1 mg/mL. Samples were further diluted with methanol to obtain a final concentration of 500 ppm. Analyses were performed on an ACQUITY UPLC System (Waters, Milford, MA, USA) coupled with a Waters Xevo G2-XS QToF Mass Spectrometer. An ACQUITY UPLC HSS T3 C18 column (2.1 × 100 mm^2^, 1.8 μm) from Waters (Waters Corporation, Wexford, Ireland) was used to carry on the chromatographic separation at 30 °C. The mobile phases consisted of eluent A (Water 0.1% formic acid, **v**/**v**) and eluent B (Acetonitrile 0.1% formic acid, *v*/*v*) with a flow rate of 0.5 mL/min. The gradient was programmed as follows: 10–100% B from 0 to 10 min, 100% B from 10 to 14 min, 100–5% B from 14 to 15 min, 5% B from 15 to 20 min. Mass spectrometry was performed on a Xevo G2-XS QToF instrument using electrospray ionization (ESI). Data mass spectra were acquired in ionization negative (ESI−) and positive (ESI+) modes, in a mass range of *m*/*z* 100–1500 Da in data dependent acquisition (DDA) mode at a rate set to 0.1 s scans, followed by an MS^2^ scan of the most intense ions. For MS^2^ fragmentation, a low collision energy ramp ranging from 10 to 40 eV and a high collision energy ramp ranging from 50 to 80 eV were applied to acquire the data in centroid format. The QTOF instrument was operated at a frequency of 6.0 GHz and 76.0 μs utilizing high resolution calibrated with the reference mass correction for Leucine-enkephalin (C_28_H_37_N_5_O_7_) and was used as a lock mass compound in positive ion mode ([M + H]^+^ = 556.2771) and negative ion mode ([M-H]^−^ = 554.2615), respectively. The spectrometer parameters were as follows: capillary voltage was set to 2.2 kV (ESI−) or 2.6 kV (ESI+), cone voltage was set to 40 V, source temperature was maintained at 120 °C, cone gas was set to 50 L/h, and desolvation gas was set to 800 L/h at 300 °C. Retention time data compared between HPLC-DAD and UHPLC-QTOF-MS analyses of the reference standards are detailed in Supporting Information Table S3.

### 5.8 ^1^H-NMR Data Acquisition

To the BLEs were added 500 μL of methanol-*d*_4_ (CD_3_OD) and 200 μL of phosphate buffer (KH_2_PO_4_, 99% anhydrous) in deuterium oxide (D_2_O) (pH 6.0) from Sigma-Aldrich USA Isotope with 99.8% purity; the solution was sonicated (frequency of 50 kHz/20 min) and centrifuged (10.000 rpm/5 min), and 700 μL of the supernatant was transferred to 5 mm NMR tubes for further 1D NMR analyses. ^1^H-NMR spectra were recorded at 25 °C on a Bruker Avance III HD 600 MHz spectrometer (Bruker, Karlsruhe, Germany) operating at a proton NMR frequency of 600.13 MHz. Each ^1^H-NMR spectrum consisted of 128 scans using the following parameters: 0.16 Hz/point, pulse width (PW) = 30 (11.3 μs), and relaxation delay (RD) = 1.5 s. Free induction decays (FIDs) were Fourier transformed with line broadening (LB) = 0.3 Hz. Deuterated methanol was used as the internal lock. Chemical shifts were expressed in δ (ppm), and coupling constants were reported in hertz. The ^1^H-NMR spectra of some reference standards were included in Supporting Information Figure S5 and Figure S6.

### 5.9 GNPS Classical Molecular MS^2^ Network

The MS^2^ data files in mzML format were submitted to the GNPS online platform (http://gnps.ucsd.edu). The molecular networking of BLEs was obtained using parameters provided in the Supporting Information, as shown in Table S4 and described by Aron et al.^70^ Classical molecular networks for both negative and positive ionization modes are accessible on the GNPS Web site at the following links:

Negative mode: https://gnps.ucsd.edu/ProteoSAFe/status.jsp?task=d4d5f88562114bf0b1a6a8083ecfb166

Positive mode: https://gnps.ucsd.edu/ProteoSAFe/status.jsp?task=f6f3a908473a462eb8822d718a087d8b

Visualization of comprehensive chemical space was incorporated in the MolNetEnhancer tool. Chemical class annotations were performed using ClassyFire chemical ontology. Both negative and positive ionization modes are accessible on the GNPS Web site at the following links:

Negative mode: https://gnps.ucsd.edu/ProteoSAFe/status.jsp?task=a4dd538e6fc845858df14c6308ff317a

Positive mode: https://gnps.ucsd.edu/ProteoSAFe/status.jsp?task=2a2f566db5994ec8a05fa024c751f1ef

The attribute table of the generated nodes was visualized in Cytoscape version 3.7.2 software to analyze the molecular network. The data used for the analysis of molecular networks were deposited in the MassIVE Public GNPS database (http://massive.ucsd.edu) with the accession number MSV000093471 for negative mode and MSV000093474 for positive mode.

### 5.10 Antioxidant Activity

The assays were carried out in triplicate for all the samples using a single extract concentration of 1 mg/mL^21,48^ diluted in ethanol and determined by the azinobis(ethylbenzothiazoline-6-sulfonic acid) (ABTS^•+^) radical scavenging method and scavenging of 1,1-diphenyl-2-picryl-hydrazyl (DPPH^•^) radical assay.

### 5.11 ABTS^•+^ Free Radical Scavenging Ability Assay

The ABTS assay was conducted following the method proposed by Lozano-Puentes et al., with some modifications.^19,71^ Briefly, the radical was generated by oxidizing ABTS at 7 mM with potassium persulfate at 2.45 mM and incubated for 12 h in darkness until the reaction was completed and the absorbance was stable. To perform the test, 30 μL of the BLEs was taken at 1 mg/mL and mixed with 970 μL of the ABTS^•+^ solution previously prepared and adjusted to an approximate absorbance value of 0.70 ± 0.02 with ethanol. This mixture was allowed to react for 30 min at room temperature in the dark. The reduction of ABTS^•+^ was measured by using a Varioskan LUX Multimode Microplate Reader (Thermo Scientific, Vantaa, Finland) at 734 nm and compared against a standard curve of Trolox. As a negative control, the solvent in which the extracts were solubilized (ethanol) was used. A Trolox calibration curve (0–100 mg/L, R^2^ = 0.9705) was constructed to express the antioxidant capacity in μmol trolox equivalents per gram of extract (μmol TE/g extract) in Supporting Information Figure S7. Three independent experiments were performed in triplicate.

### 5.12 DPPH^•^ Free Radical Scavenging Ability Assay

Antioxidant activity was measured using the DPPH^•^ radical method described by Lozano-Puentes et al., with some modifications.^19,72^ The electron-capturing capacity was determined by reacting 280 μL of a DPPH^•^ solution at 240 mg/L with 20 μL of BLEs at 1 mg/mL for 30 min under conditions of darkness and room temperature. Radical reduction was measured using a Varioskan LUX Multimode Microplate Reader (Thermo Scientific, Vantaa, Finland) at 517 nm and compared against a standard curve of Trolox. As a negative control, the solvent in which the extracts were solubilized (ethanol) was used. A calibration curve of trolox (0–100 mg/L, R^2^ = 0.9709) was conducted to express the antioxidant capacity in μmol trolox equivalents per gram of extract (μmol TE/g extract) in Supporting Information Figure S7. Three independent experiments were performed in triplicate.

### 5.13 Cytotoxic Activity

The cell cultures of the human colorectal carcinoma cell line HCT-116 were acquired from the American Type Culture Collection (ATCC, Rockville, MD, USA) and maintained under standard cell culture conditions (37 °C, 95% with 5% CO_2_ atmosphere) in RPMI-1640 medium supplemented with 10% FBS, 2 g/L NaHCO_3_, and 1% antibiotics (penicillin and streptomycin).

### 5.14 MTT Cell Viability Assay

Colorimetric 3-(4,5-dimethylthiazol-2-yl)-2,5-diphenyltetrazolium bromide (MTT) assay was used to evaluate metabolic activity.^73^ Approximately 1 × 10^4^ cells per well were seeded in 96-well plates. Twenty-four hours after seeding, the cells were then treated with DMSO 0.05% (vehicle), doxorubicin 10 μM (control), and BLEs at two concentrations of 5 and 50 μg/mL (treatments) diluted in DMSO and incubated under standard cell culture conditions for 72 h. Subsequently, MTT solution (150 μL at 10%) was added to each control and treatment and incubated again for 3 h. Then the medium was removed and the formazan crystal was solubilized with 200 μL of DMSO. The Bio-Tek Synergy HTX multimode reader (BioTeck Instruments) was applied to read the absorbance at 570 nm. Percent growth inhibition was calculated with eq 1:
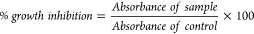
1

To determine the dose–response curves (percentage cell survival versus concentration) and to obtain the IC_50_ value (the minimum concentration of each ethanol extract that provides 50% survival of HCT-116 cells), extracts that exceeded 85% inhibition of cell growth were prepared over a range of concentrations from 3.2 × 10^–6^ to 5.0 × 10^–2^ mg/mL, following the same procedure explained above. Data were determined in three independent biological replicates, each with technical quadruplicates. IC_50_ curves and extract values were calculated using GraphPad Prism version 8.0.2 software (San Diego, CA, USA) using sigmoidal dose–response curve fitting (variable slope, four parameters).

### 5.15 Data Processing and Biochemometric Analysis

The raw UHPLC-QTOF-MS data files were converted into mzML format using the MSConvert tool from ProteoWizard software version 3.0 (Palo Alto, CA, USA) to separate negative and positive ionization modes. The files were processed in MZmine version 2.53 (http://mzmine.github.io/download.html), including feature detection, chromatogram builder, deconvolution, deisotopes, alignment, filtering, and gap filling whose parameters summarized in Supporting Information Table S5. The list of data information, including *t*_*R*_-*m*/*z* (characterize the detected ions) and normalized peak area were exported as .csv files and manually inspected to eliminate noise, and presence filter was applied. The features that were present in 100% of the samples and had a coefficient of variation in the QC of less than 20% were used for statistical analysis.

The raw ^1^H-NMR spectra were imported into NMRProcFlow version 1.4.20 (https://www.nmrprocflow.org) for ppm calibration, baseline correction, alignment, spectra bucketing, and data normalization whose parameters summarized in Supporting Information Table S6. Regions at δ_H_ 4.85–4.95 and δ_H_ 3.25–3.35 ppm were removed from the analysis due to residual solvents signals of D_2_O and CD_3_OD, respectively. Spectra bucketing utilized the intelligent bucketing method and variable size bucketing, encompassing the full range of δ_H_ 0.40–10.0 ppm.

Statistical analysis was performed with SIMCA-P+ software, version 18.0 (Umetrics, Umeå, Sweden). The multivariate analysis allowed the generation of PCA and OPLS-DA models. The OPLS-DA model was validated by cross-validation less than 0.05, and quality was evaluated based on parameters R^2^X, R^2^Y, and Q^2^. The groups were compared to determine the significantly differential metabolites by calculating the variable importance in the projection (VIP) combined with FC > 2.0 or FC < 0.5. Annotated metabolites were organized in a table (.csv) containing the peak area were uploaded at MetaboAnalyst 5.0 software–statistical, functional, and integrative analysis of metabolomics data (https://www.metaboanalyst.ca/) was used visualization for heatmap clustering,^74^ normalized by Pareto scaling. Univariate analysis was performed to determine the *p*-value features.

### 5.16 Metabolite Identification

Differential metabolite identification involved mass accuracy (with a maximum error 10 ppm), isotopic pattern distribution (to generate of molecular formulas), and adduct formation. After that, we utilized the CEU Mass Mediator tool (https://ceumass.eps.uspceu.es/) to search for potential metabolites candidates through different public online databases such as METLIN (http://metlin.scripps.edu), KEGG (https://www.genome.jp/kegg/), HMDB (https://hmdb.ca/), PubChem (https://pubchem.ncbi.nlm.nih.gov/) and ChEBI (https://www.ebi.ac.uk/chebi/). We confirmed the identity of the metabolites through MS^2^ analysis, employing tools such as MS-DIAL 4.80 (http://prime.psc.riken.jp/compms/msdial/main.html), MS-FINDER 3.52 (http://prime.psc.riken.jp/compms/msfinder/main.html), SIRIUS 5.5.7 (https://bio.informatik.uni-jena.de/software/sirius/), CFM-ID 4.0 (https://cfmid.wishartlab.com/) for in silico mass spectral fragmentation. This process also included the use of the GNPS web platform (https://gnps.ucsd.edu/ProteoSAFe/static/gnps-splash.jsp) and manual interpretation with the MassLynx software version 4.01 (Waters Company, Milford, MA, USA). Furthermore, an *in-house* library was developed using metabolites previously isolated from the bamboo species subfamily (Subfamily: Bambusoideae) within the research group, facilitating the confirmation of metabolite identities. Finally, identification level were assigned for each metabolite accordance with Metabolomics Standards Initiative (MSI).^75^

## Supporting Information Available

UV spectra of the major peaks found in the BLEs obtained by HPLC-DAD; Phytochemicals tentatively identified in BLEs using UHPLC-QTOF-MS; Chemical structures of the metabolites were found in BLEs; MS^2^ spectrum match feature of GNPS showing the similarity of fragments patterns of the experimental and library data; PCA score plot including Quality Controls (QCs) and all samples from UHPLC-QTOF-MS analysis; List and information on the environmental variables of collection of the *Guadua* species used for the study; Comparison of retention times of reference standards between HPLC-DAD and UHPLC-QTOF-MS analyses; ^1^H-NMR spectra obtained for the reference standards (Quercetin and Rutin); Calibration curves obtained for the determination of antioxidant capacity by ABTS and DPPH; Parameters used in the GNPS Classical Molecular Networking platform, MZmine version 2.53, and NMRProcFlow software for processing the data obtained by UHPLC-QTOF-MS, and ^1^H-NMR (PDF)

## Abbreviations

**HPTLC**: High-Performance Thin-Layer Chromatography
**HPLC-DAD**: High-Performance Liquid Chromatography coupled with Diode Array Detector
**UHPLC-QTOF-MS**: Ultra-High Performance Liquid Chromatography coupled with Quadrupole Time-of-Flight Mass Spectrometry
^**1**^**H-NMR**: Proton Nuclear Magnetic Resonance
**MIC**: Minimal Inhibitory Concentration
