# Supplementary material for: Metabolomic Profiling of Guadua Species and Its Correlation with Antioxidant and Cytotoxic Activities
Source: ACS Omega. 2024 Aug 21;9(35):36939–60. doi: 10.1021/acsomega.3c09114 (PMC11375805; doi:10.1021/acsomega.3c09114)
Supplement: Supplementary file 1 — ao3c09114_si_001.pdf [file ao3c09114_si_001.pdf]

Supplementary Material

for

Metabolomic Profiling of *Guadua* Species and its Correlation with Antioxidant and Cytotoxic Activities

Luis Carlos Chitiva[a,b], Paula Rezende-Teixeira[c], Tiago F. Leão[a], Hair Santiago Lozano-Puentes[d], Ximena Londoño[e], Lucía Ana Díaz-Ariza[d], Leticia V. Costa-Lotufo[c], Juliet A. Prieto-Rodríguez[b], Geison M. Costa[b], and Ian Castro-Gamboa[a,*]

[a]*Núcleo de Bioensaios, Biossíntese e Ecofisiologia de Produtos Naturais (NuBBE), Institute of Chemistry, São Paulo State University (UNESP), SP, Araraquara, SP 14800-901, Brazil*

[b]*Grupo de Investigación Fitoquímica Universidad Javeriana (GIFUJ), Department of Chemistry, Faculty of Sciences, Pontificia Universidad Javeriana, Bogotá 110231, Colombia*

[c]*Laboratório de Farmacologia de Produtos Naturais Marinhos, Institute of Biomedical Sciences, University of São Paulo, São Paulo, SP 05508-000, Brazil*

[d]*Laboratorio Asociaciones, Suelo, Planta, Microorganismo (LAMIC), Grupo de Investigación en Agricultura Biológica, Department of Biology, Faculty of Sciences, Pontificia Universidad Javeriana, Bogotá 110231, Colombia*

[e]*Faculty of Agricultural Sciences, Universidad Nacional de Colombia, Palmira 763533, Colombia*

*Corresponding author.

E-mail address: ian.castro@unesp.br (Ian Castro-Gamboa)

Table of Contents

1. **Figure S1.** UV spectra of the major peaks found in the BLEs obtained by HPLC-DAD.

2. **Table S1.** Phytochemicals tentatively identified in BLEs using UHPLC-QTOF-MS.

3. **Figure S2.** Chemical structures of the metabolites were found in BLEs.

4. **Figure S3.** MS^2 spectrum match feature of GNPS showing the similarity of fragments patterns of the experimental and library data.

5. **Figure S4.** PCA score plot including Quality Controls (QCs) and all samples from UHPLC-QTOF-MS analysis.

6. **Table S2.** List and information on the environmental variables of collection of the *Guadua* species used for the study.

7. **Table S3.** Comparison of retention times of reference standards between HPLC-DAD and UHPLC-QTOF-MS analyses.

8. **Figure S5.** ^{1}H-NMR spectra obtained for quercetin.

9. **Figure S6.** ^{1}H-NMR spectra obtained for rutin.

10. **Table S4.** Parameters used in the GNPS Classical Molecular Networking.

11. **Table S5.** Parameters used in the MZmine 2.53 software for processing the data obtained by UHPLC-QTOF-MS.

12. **Table S6.** Parameters used in the NMRProcFlow software for processing the data obtained by ^{1}H-NMR.

13. **Figure S7.** Calibration curves obtained for the determination of antioxidant capacity by ABTS and DPPH.

14. References

Brief description of contents: (i) UV spectra of the major peaks found in the BLEs obtained by HPLC-DAD; (ii) Phytochemicals tentatively identified in BLEs using UHPLC-QTOF-MS; (iii) Chemical structures of the metabolites were found in BLEs; (iv) MS^2 spectrum match feature of GNPS showing the similarity of fragments patterns of the experimental and library data; (v) PCA score plot including Quality Controls (QCs) and all samples from UHPLC-QTOF-MS analysis; (vi) List and information on the environmental variables of collection of the *Guadua* species used for the study; (vii) Comparison of retention times of reference standards between HPLC-DAD and UHPLC-QTOF-MS analyses; (viii) ^{1}H-NMR spectra obtained for the reference standards (Quercetin and Rutin); (ix) Calibration curves obtained for the determination of antioxidant capacity by ABTS and DPPH (x) Parameters used in the GNPS Classical Molecular Networking platform, MZmine version 2.53, and NMRProcFlow software for processing the data obtained by UHPLC-QTOF-MS, and ^{1}H-NMR.

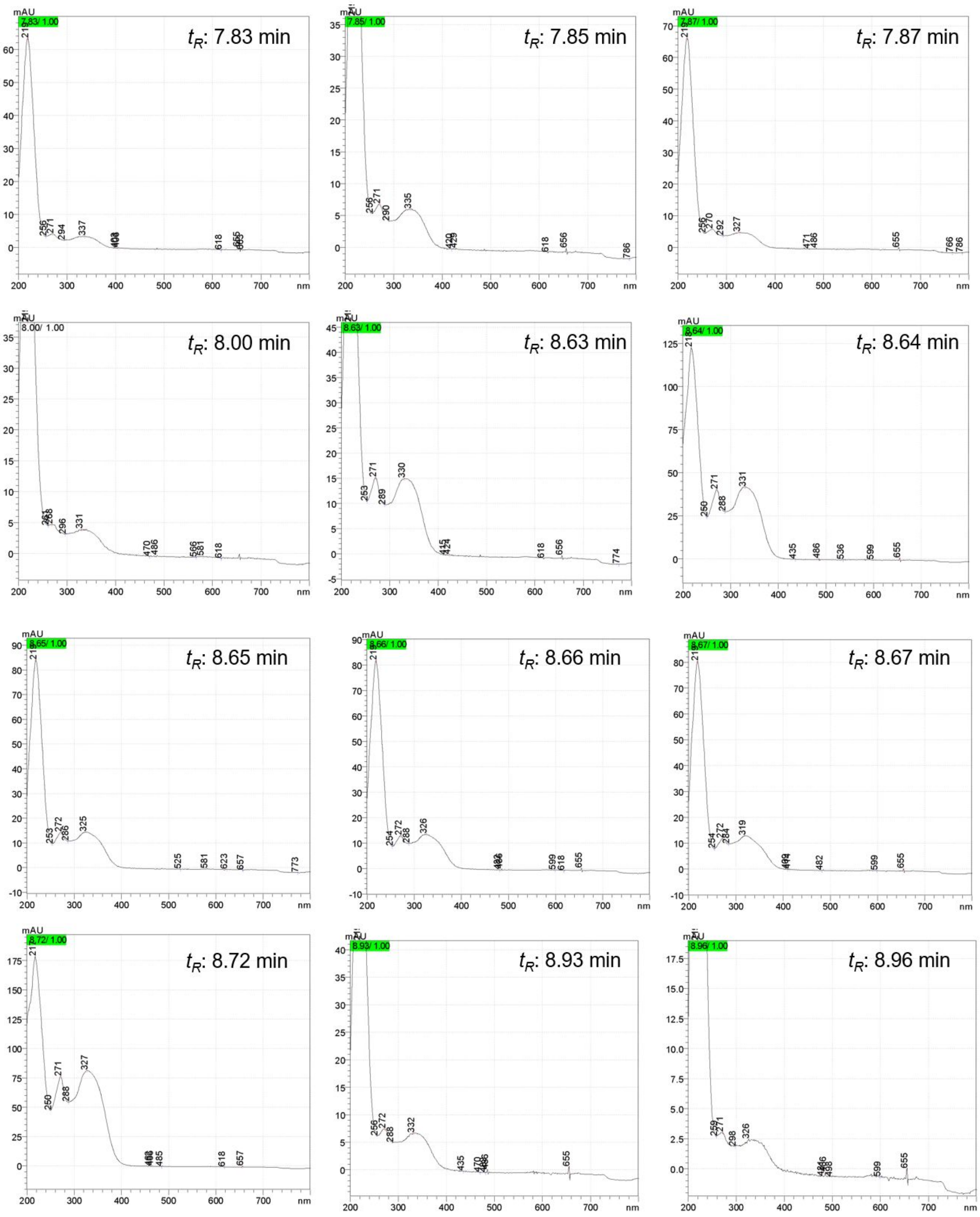

t_R: 7.83 min
t_R: 7.85 min
t_R: 7.87 min
t_R: 8.00 min
t_R: 8.63 min
t_R: 8.64 min
t_R: 8.65 min
t_R: 8.66 min
t_R: 8.67 min
t_R: 8.72 min
t_R: 8.93 min
t_R: 8.96 min
mAU
nm

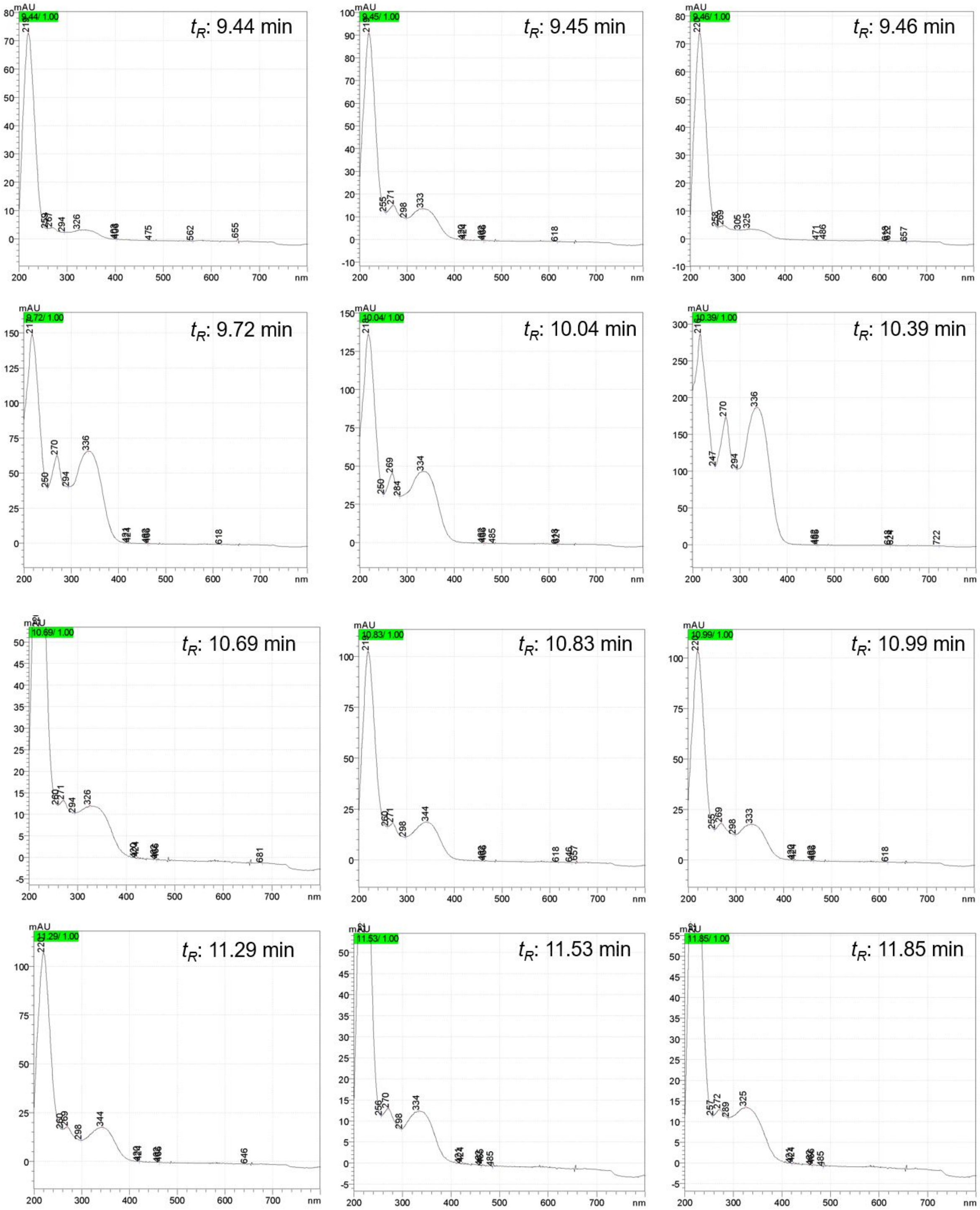
t_R: 9.44 min
t_R: 9.45 min
t_R: 9.46 min
t_R: 9.72 min
t_R: 10.04 min
t_R: 10.39 min
t_R: 10.69 min
t_R: 10.83 min
t_R: 10.99 min
t_R: 11.29 min
t_R: 11.53 min
t_R: 11.85 min

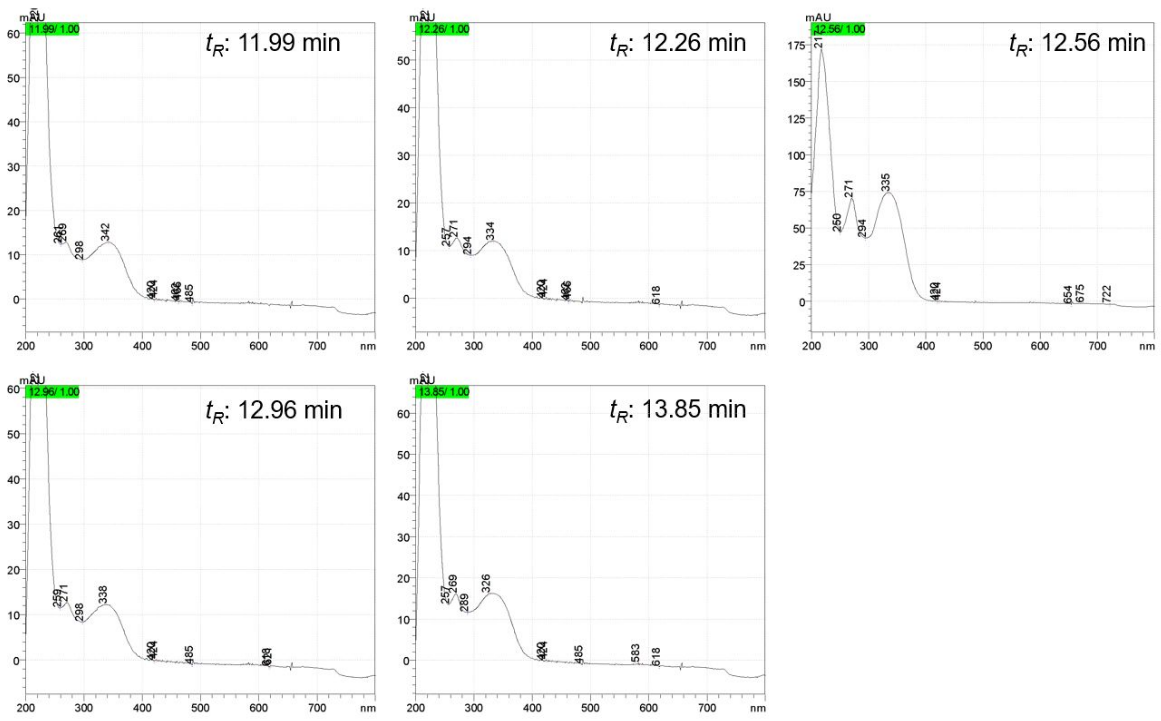

Figure S1. UV spectra of the major peaks found in the BLEs obtained by HPLC-DAD. The retention time of each peak and its corresponding spectrum are described in highlighted green (t_R/1.00).

Table S1. Phytochemicals tentatively identified in BLEs using UHPLC-QTOF-MS.

Peak	t_R (min)	UV λ_{max} (nm)	Molecular formula	Adduct ion	Calculated mass (*m/z*)	Experimental mass (*m/z*)	Mass error (ppm)	Main fragment ions (MS/MS)	Identification confidence level	Tentative identification	Chemical class	Plant species	Reference or Database[a]
**15**	0.57	-	$C_7H_{12}O_6$	$[M-H]^-$	191.0551	191.0543	-4.2	175, 147, 129, 111	2	Quinic acid	Organic acid	*G. aculeata*<br>*G. amplexifolia*<br>*G. angustifolia*<br>*G. angustifolia* biotype *San calixto*<br>*G. angustifolia* var. *bicolor*<br>*G. incana*<br>*G. superba*<br>*G. uncinata*<br>*G. venezuelae*<br>*G. weberbaueri*	[1–3]
**16**	1.20	Low intensity	$C_{16}H_{18}O_9$	$[M-H]^-$	353.0878	353.0861	-4.8	191, 179, 155, 135	3	Not identified	Cinnamic acid and derivatives	*G. superba*<br>*G. weberbaueri*	
**17**	1.94	Low intensity	$C_{15}H_{16}O_{10}$	$[M-H]^-$	355.0670	355.0669	-0.3	209, 191, 163, 147, 129	3	Not identified	Cinnamic acid and derivatives	*G. aculeata*<br>*G. amplexifolia*<br>*G. angustifolia*<br>*G. incana*	
**18**	1.98	Low intensity	$C_{17}H_{20}O_9$	$[M-H]^-$	367.1034	367.1024	-2.7	207, 193, 134	2	*O*-Feruloylquinic acid	Cinnamic acid and derivatives	*G. angustifolia*<br>*G. superba*<br>*G. uncinata*	[3–7] and GNPS
**19**	2.04	290^{sh}, 310	$C_{25}H_{24}O_{12}$	$[M-H]^-$	515.1195	515.1193	-0.4	353, 191, 179, 137	2	Dicaffeoylquinic acid	Cinnamic acid and derivatives	*G. aculeata*<br>*G. superba*<br>*G. venezuelae*<br>*G. weberbaueri*	[3] and GNPS

**20**	2.33	Low intensity	$C_{16}H_{18}O_8$	$[M-H]^-$	337.0928	337.0945	5.0	191, 173, 164, 119	2	*O*-Coumaroylquinic acid	Cinnamic acid and derivatives	*G. aculeata*<br>*G. superba*<br>*G. venezuelae*<br>*G. weberbaueri*	[1,3,8,9] and GNPS
**21**	2.36	270, 327	$C_{27}H_{30}O_{16}$	$[M-H]^-$	609.1461	609.1466	0.8	547, 519, 489, 429, 399	3	Luteolin 6-*C*-hexoside 8-*C*-pentoside	Flavone glycosides	*G. amplexifolia*<br>*G. angustifolia*<br>*G. angustifolia* biotype *San calixto*<br>*G. angustifolia* var. *bicolor*<br>*G. incana*<br>*G. uncinata*	
**2**	2.46	292[sh], 326	$C_{16}H_{18}O_9$	$[M-H]^-$	353.0878	353.0864	-4.0	191, 179, 135	1	Chlorogenic acid[st]	Cinnamic acid and derivatives	*G. aculeata*<br>*G. superba*<br>*G. venezuelae*<br>*G. weberbaueri*	[10–13] and GNPS
**22**	2.51	274, 336	$C_{21}H_{20}O_{10}$	$[M-H]^-$	431.0983	431.0981	-0.5	387, 341, 311, 179	3	Not identified	Flavone glycosides	*G. aculeata*<br>*G. amplexifolia*<br>*G. angustifolia*<br>*G. angustifolia* biotype *San calixto*<br>*G. angustifolia* var. *bicolor*<br>*G. incana*<br>*G. superba*<br>*G. uncinata*	

**23**	2.68	272, 325	$C_{27}H_{30}O_{15}$	$[M-H]^-$	593.1511	593.1513	0.3	503, 473, 383, 353, 297	2	Vicenin 2	Flavone glycosides	*G. amplexifolia*<br>*G. angustifolia*<br>*G. angustifolia* biotype *San calixto*<br>*G. incana*<br>*G. superba*<br>*G. uncinata*<br>*G. venezuelae*	[1,14] and GNPS
**24**	2.70	269, 325	$C_{21}H_{20}O_{11}$	$[M-H]^-$	447.0932	447.0931	-0.2	327, 299, 285, 133, 109	3	Not identified	Flavone glycosides	*G. amplexifolia*<br>*G. angustifolia*<br>*G. angustifolia* var. *bicolor*<br>*G. incana*<br>*G. uncinata*	
**25**	2.71	268, 337	$C_{27}H_{30}O_{15}$	$[M-H]^-$	593.1511	593.1517	1.0	457, 383, 353, 297	2	Apigenin 6,8-digalactoside	Flavone glycosides	*G. amplexifolia*<br>*G. angustifolia*<br>*G. angustifolia* biotype *San calixto*<br>*G. incana*<br>*G. superba*<br>*G. uncinata*<br>*G. venezuelae*	[8] and GNPS
				$[M+H]^+$	595.1668	595.1660	-1.3	541, 481, 457, 379, 325, 295					
**26**	2.71	267, 326	$C_{26}H_{28}O_{14}$	$[M-H]^-$	563.1406	563.1411	0.9	473, 443, 383, 353, 325, 2.97	3	Not identified	Flavone glycosides	*G. amplexifolia*<br>*G. angustifolia*<br>*G. angustifolia* var. *bicolor*<br>*G. incana*<br>*G. superba*<br>*G. uncinata*<br>*G. venezuelae*<br>*G. weberbaueri*	

**3**	2.74	311	$C_9H_8O_4$	$[M-H]^-$	179.0349	179.0352	1.7	135, 119	1	Caffeic acid[st]	Cinnamic acid and derivatives	*G. aculeata*<br>*G. amplexifolia*<br>*G. angustifolia*<br>*G. angustifolia* biotype *San calixto*<br>*G. incana*<br>*G. superba*<br>*G. uncinata*<br>*G. venezuelae*	[10,11,15]
**27**	2.77	271, 333	$C_{26}H_{28}O_{14}$	$[M-H]^-$	563.1406	563.1410	0.7	445, 355, 325, 297	3	Not identified	Flavone glycosides	*G. angustifolia*<br>*G. angustifolia* biotype *San calixto*<br>*G. angustifolia* var. *bicolor*<br>*G. incana*<br>*G. superba*<br>*G. uncinata*	
**28**	2.80	269, 334	$C_{26}H_{28}O_{14}$	$[M-H]^-$	563.1416	563.1415	-0.2	473, 443, 401, 383, 353, 311	3	Not identified	Flavone glycosides	*G. aculeata*<br>*G. amplexifolia*<br>*G. angustifolia*<br>*G. angustifolia* biotype *San calixto*<br>*G. incana*<br>*G. superba*<br>*G. uncinata*<br>*G. venezuelae*	
**29**	2.87	265, 341	$C_{27}H_{30}O_{14}$	$[M-H]^-$	577.1562	577.1565	0.5	413, 341, 293, 175	2	Vitexin 2''-*O*-rhamnoside	Flavone glycosides	*G. angustifolia*<br>*G. angustifolia* biotype *San calixto*<br>*G. angustifolia* var. *bicolor*	[1] and GNPS
				$[M+H]^+$	579.1719	579.171	-1.6	433, 415, 367, 313, 283					

												G. incana<br>*G. superba*<br>*G. uncinata*	
**30**	2.88	269, 342	$C_{27}H_{30}O_{15}$	$[M-H]^-$	593.1511	593.1519	1.3	473, 431, 353, 311, 297, 282	2	Saponarin or Isosaponarin	Flavone glycosides	*G. amplexifolia*<br>*G. angustifolia*<br>*G. angustifolia* var. *bicolor*<br>*G. incana*<br>*G. superba*<br>*G. uncinata*<br>*G. venezuelae*<br>*G. weberbaueri*	[1,8] and GNPS
**31**	2.91	271, 335	$C_{26}H_{28}O_{14}$	$[M-H]^-$	563.1406	563.1401	-0.9	473, 443, 383, 353, 325, 297	2	Schaftoside or Isoschaftoside	Flavone glycosides	*G. amplexifolia*<br>*G. angustifolia*<br>*G. angustifolia* biotype *San calixto*<br>*G. angustifolia* var. *bicolor*<br>*G. incana*<br>*G. uncinata*<br>*G. venezuelae*<br>*G. weberbaueri*	[1,3,4,8,9,16] and GNPS
				$[M+H]^+$	565.1562	565.1563	0.2	547, 529, 499, 457, 427, 379					
**32**	3.13	273, 339	$C_{26}H_{28}O_{14}$	$[M+H]^+$	565.1562	565.1564	0.4	525, 481, 405, 337, 295	3	Vicenin 1 or Vicenin 3	Flavone glycosides	*G. amplexifolia*<br>*G. angustifolia*<br>*G. angustifolia* var. *bicolor*<br>*G. incana*<br>*G. superba*<br>*G. uncinata*<br>*G. venezuelae*<br>*G. weberbaueri*	

**33**	3.16	270, 344	$C_{28}H_{32}O_{16}$	$[M-H]^-$	623.1617	623.1617	0.0	504, 443, 353, 323	3	Isoscoparin 2''-*O*-hexoside	Flavone glycosides	*G. amplexifolia*<br>*G. angustifolia*<br>*G. angustifolia* biotype *San calixto*<br>*G. angustifolia* var. *bicolor*<br>*G. incana*<br>*G. uncinata*	
**34**	3.18	271, 342	$C_{28}H_{32}O_{15}$	$[M+H]^+$	609.1824	609.1824	0.0	430, 393, 327, 297, 267	3	Spinosin or Isospinosin	Flavone glycosides	*G. amplexifolia*<br>*G. angustifolia*<br>*G. angustifolia* var. *bicolor*<br>*G. incana*<br>*G. superba*<br>*G. uncinata*<br>*G. venezuelae*<br>*G. weberbaueri*	
**8**	3.20	Low intensity	$C_9H_8O_3$	$[M-H]^-$	163.0400	163.0400	0.0	119	1	*p*-Coumaric acidst	Cinnamic acid and derivatives	*G. aculeata*<br>*G. amplexifolia*<br>*G. angustifolia*<br>*G. angustifolia* biotype *San calixto*<br>*G. incana*<br>*G. superba*<br>*G. uncinata*<br>*G. venezuelae*	[17] and GNPS

**35**	3.23	270, 341	$C_{27}H_{30}O_{14}$	$[M-H]^-$	577.1562	577.1569	1.2	533, 472, 413, 353, 293	2	Violanthin or Isoviolanthin	Flavone glycosides	*G. amplexifolia*<br>*G. angustifolia*<br>*G. angustifolia* var. *bicolor*<br>*G. incana*<br>*G. superba*<br>*G. uncinata*<br>*G. venezuelae*<br>*G. weberbaueri*	[18]
**36**	3.24	269, 332	$C_{27}H_{30}O_{15}$	$[M-H]^-$	593.1511	593.1512	0.2	285, 218, 151	2	Kaempferol 7-*O*-neohesperidoside	Flavonol glycosides	*G. amplexifolia*<br>*G. angustifolia*<br>*G. angustifolia* biotype *San calixto*<br>*G. angustifolia* var. *bicolor*<br>*G. incana*<br>*G. uncinata*	[18] and GNPS
**37**	3.28	270, 325	$C_{33}H_{40}O_{19}$	$[M-H]^-$	739.2091	739.209	-0.1	593, 431, 281	3	Kaempferol 3-*O*-disaccharoside-7-*O*-pentoside	Flavonol glycosides	*G. amplexifolia*<br>*G. angustifolia*<br>*G. angustifolia* biotype *San calixto*<br>*G. angustifolia* var. *bicolor*<br>*G. incana*<br>*G. uncinata*	

**38**	3.28	270, 326	$C_{27}H_{30}O_{15}$	$[M+H]^+$	595.1668	595.1667	-0.2	449, 287	3	Kaempferol 3-*O*-disaccharoside	Flavonol glycosides	*G. amplexifolia*<br>*G. angustifolia*<br>*G. angustifolia* biotype *San calixto*<br>*G. angustifolia* var. *bicolor*<br>*G. incana*<br>*G. uncinata*	
**39**	3.35	272, 326	$C_{21}H_{18}O_{13}$	$[M-H]^-$	477.0674	477.0675	0.2	431, 301, 179	3	Quercetin 3-*O*-hexoside	Flavonol glycosides	*G. amplexifolia*<br>*G. angustifolia*<br>*G. angustifolia* var. *bicolor*<br>*G. incana*<br>*G. superba*<br>*G. uncinata*<br>*G. venezuelae*<br>*G. weberbaueri*	
**40**	3.37	270, 325	$C_{21}H_{20}O_{12}$	$[M-H]^-$	463.0882	463.0880	-0.4	372, 300, 271, 255, 243, 151	2	Hyperoside	Flavonol glycosides	*G. aculeata*<br>*G. angustifolia*<br>*G. angustifolia* biotype *San calixto*<br>*G. angustifolia* var. *bicolor*<br>*G. incana*<br>*G. superba*<br>*G. uncinata*<br>*G. venezuelae*	[17]

**41**	3.41	271, 329	$C_{28}H_{32}O_{17}$	$[M-H]^-$	639.1566	639.1570	0.6	403, 328, 313, 285, 270, 242	3	Isorhamnetin hexoside	3,4'-	Flavonol glycosides	*G. amplexifolia*<br>*G. angustifolia*<br>*G. angustifolia* var. *bicolor*<br>*G. incana*<br>*G. superba*<br>*G. uncinata*<br>*G. venezuelae*<br>*G. weberbaueri*
**42**	3.54	271, 344	$C_{27}H_{30}O_{14}$	$[M-H]^-$	577.1562	577.1561	-0.2	269, 117	3	Apigenin disaccharoside	7-*O*-	Flavone glycosides	*G. amplexifolia*<br>*G. angustifolia*<br>*G. angustifolia* biotype *San calixto*<br>*G. angustifolia* var. *bicolor*<br>*G. incana*<br>*G. uncinata*<br>*G. venezuelae*
**43**	3.56	269, 342	$C_{27}H_{30}O_{14}$	$[M+H]^+$	579.1719	579.1718	-0.2	433, 363, 271, 153	3	Apigenin disaccharoside	7-*O*-	Flavone glycosides	*G. amplexifolia*<br>*G. angustifolia*<br>*G. angustifolia* biotype *San calixto*<br>*G. angustifolia* var. *bicolor*<br>*G. incana*<br>*G. superba*<br>*G. uncinata*<br>*G. venezuelae*<br>*G. weberbaueri*

**44**	3.58	268, 331	$C_{22}H_{22}O_{11}$	$[M-H]^-$	461.1089	461.1087	-0.4	415, 341, 313, 298	3	Not identified	Flavone glycosides	*G. amplexifolia*<br>*G. angustifolia*<br>*G. angustifolia* biotype *San calixto*<br>*G. angustifolia* var. *bicolor*<br>*G. incana*<br>*G. uncinata*<br>*G. venezuelae*
**45**	3.59	270, 325	$C_{28}H_{34}O_{15}$	$[M-H]^-$	609.1824	609.1843	3.1	325, 301, 286, 151	3	Hesperidin	Flavanone glycoside	*G. amplexifolia*<br>*G. angustifolia*<br>*G. angustifolia* biotype *San calixto*<br>*G. angustifolia* var. *bicolor*<br>*G. incana*<br>*G. uncinata*
**46**	3.61	271, 344	$C_{28}H_{32}O_{15}$	$[M+H]^+$	609.1826	609.1810	-2.6	463, 301, 286, 258	3	Diosmetin 7-*O*-disaccharoside	Flavone glycosides	*G. amplexifolia*<br>*G. angustifolia*<br>*G. angustifolia* biotype *San calixto*<br>*G. angustifolia* var. *bicolor*<br>*G. incana*<br>*G. superba*<br>*G. uncinata*<br>*G. venezuelae*

**47**	3.64	269, 326	$C_{28}H_{32}O_{16}$	$[M-H]^-$	623.1617	623.1620	0.5	329, 314, 299, 271, 243, 187	3	Isorhamnetin 3-*O*-hexoside-6''-disaccharoside	Flavonol glycosides	*G. aculeata*<br>*G. angustifolia*<br>*G. angustifolia* biotype *San calixto*<br>*G. angustifolia* var. *bicolor*<br>*G. incana*<br>*G. superba*<br>*G. uncinata*<br>*G. venezuelae*	
**48**	3.73	265, 341	$C_{27}H_{30}O_{15}$	$[M-H]^-$	593.1511	593.1512	0.2	473, 431, 353, 341, 311, 283	3	Apigenin 6-*C*-hexoside-7-*O*-hexoside	Flavone glycosides	*G. amplexifolia*<br>*G. angustifolia*<br>*G. angustifolia* biotype *San calixto*<br>*G. angustifolia* var. *bicolor*<br>*G. incana*<br>*G. uncinata*<br>*G. venezuelae*	
				$[M+H]^+$	595.1668	595.1667	-0.2	415, 379, 337, 313, 283, 165					
**49**	4.47	264, 356	$C_{15}H_{10}O_6$	$[M-H]^-$	285.0404	285.0403	-0.4	199, 169, 151, 133	2	Luteolin	Flavone aglycones	*G. aculeata*<br>*G. incana*<br>*G. superba*<br>*G. uncinata*<br>*G. venezuelae*<br>*G. weberbaueri*	[12,19,20]
**50**	4.57	270, 319	$C_{15}H_{12}O_3$	$[M-H]^-$	239.0713	239.0699	-5.9	211, 195, 179, 135	3	6-Hidroxyflavanone	Flavone aglycones	*G. amplexifolia*<br>*G. angustifolia*<br>*G. angustifolia* biotype *San calixto*<br>*G. angustifolia* var.	

												bicolor<br>*G. incana*	
**51**	4.88	264, 329	$C_{23}H_{24}O_{12}$	$[M-H]^-$	491.1195	491.1198	0.6	461, 328, 313, 285, 226	2	Tricin 7-*O*-glucoside	Flavone glycosides	*G. aculeata*<br>*G. amplexifolia*<br>*G. angustifolia*<br>*G. angustifolia* var. *bicolor*<br>*G. incana*<br>*G. superba*<br>*G. uncinata*<br>*G. venezuelae*	[8]
**52**	4.92	264, 335	$C_{27}H_{32}O_{14}$	$[M-H]^-$	579.1719	579.1720	0.2	271, 177, 151, 119	3	Naringenin-7-*O*-disaccharoside	Flavanone glycosides	*G. amplexifolia*<br>*G. angustifolia*<br>*G. angustifolia* biotype *San calixto*<br>*G. angustifolia* var. *bicolor*<br>*G. incana*<br>*G. uncinata*	

**53**	4.95	263, 334	$C_{21}H_{22}O_{10}$	$[M-H]^-$	433.1140	433.1145	1.2	271, 151, 119	3	Naringenin-7-*O*-hexoside	Flavanone glycosides	*G. aculeata*<br>*G. amplexifolia*<br>*G. angustifolia*<br>*G. angustifolia* biotype *San calixto*<br>*G. angustifolia* var. *bicolor*<br>*G. incana*<br>*G. superba*<br>*G. uncinata*<br>*G. venezuelae*<br>*G. weberbaueri*	
**54**	4.97	271, 358	$C_{15}H_{10}O_5$	$[M-H]^-$	269.0455	269.0460	1.9	227, 181, 151, 117	2	Apigenin	Flavone aglycones	*G. incana*<br>*G. superba*<br>*G. uncinata*<br>*G. venezuelae*<br>*G. weberbaueri*	[19]
**55**	5.01	244, 315	$C_{15}H_{10}O_6$	$[M-H]^-$	285.0404	285.0405	0.4	211, 183, 149, 121	3	3,6,2',3'-Tetrahydroxyflavone	Flavone aglycones	*G. aculeata*<br>*G. amplexifolia*<br>*G. angustifolia*<br>*G. angustifolia* biotype *San calixto*<br>*G. angustifolia* var. *bicolor*<br>*G. incana*<br>*G. superba*<br>*G. uncinata*<br>*G. venezuelae*<br>*G. weberbaueri*	

**56**	5.03	245, 319	$C_{15}H_{10}O_5$	$[M-H]^-$	269.0455	269.0461	2.2	239, 211, 187, 143, 117	3	6,7,4'-Trihydroxyisoflavone	Flavone aglycones	*G. aculeata*<br>*G. amplexifolia*<br>*G. angustifolia*<br>*G. angustifolia* biotype *San calixto*<br>*G. angustifolia* var. *bicolor*<br>*G. incana*<br>*G. superba*<br>*G. uncinata*<br>*G. venezuelae*<br>*G. weberbaueri*	
**57**	5.04	269, 354	$C_{15}H_{10}O_6$	$[M-H]^-$	285.0404	285.0420	5.6	255, 227, 187, 159, 143, 117	2	Kaempferol	Flavonol aglycones	*G. aculeata*<br>*G. angustifolia* var. *bicolor*<br>*G. incana*<br>*G. superba*<br>*G. uncinata*<br>*G. venezuelae*<br>*G. weberbaueri*	[18,19]
**58**	5.09	245, 319	$C_{17}H_{14}O_7$	$[M-H]^-$	329.0666	329.0671	1.5	271, 243, 227, 161, 133	3	4',5,7-Trihydroxy-3,6-dimethoxyflavone	Flavone aglycones	*G. aculeata*<br>*G. amplexifolia*<br>*G. angustifolia*<br>*G. angustifolia* biotype *San calixto*<br>*G. angustifolia* var. *bicolor*<br>*G. incana*<br>*G. superba*<br>*G. uncinata*	

												G. venezuelae<br>*G. weberbaueri*	
**59**	5.12	244, 325	$C_{16}H_{12}O_6$	$[M-H]^-$	299.0561	299.0561	0.0	256, 227, 212, 183, 151	3	3,5,7-Trihydroxy-4'-methoxyflavone	Flavone aglycones	*G. aculeata*<br>*G. amplexifolia*<br>*G. angustifolia*<br>*G. angustifolia* biotype *San calixto*<br>*G. angustifolia* var. *bicolor*<br>*G. incana*<br>*G. superba*<br>*G. uncinata*<br>*G. venezuelae*<br>*G. weberbaueri*	
**60**	5.15	244, 354	$C_{17}H_{14}O_7$	$[M-H]^-$	329.0666	329.0669	0.9	229, 271, 227, 215, 161	2	Tricin	Flavone aglycones	*G. incana*<br>*G. superba*<br>*G. uncinata*<br>*G. venezuelae*<br>*G. weberbaueri*	[19]
				$[M+H]^+$	331.0823	331.0820	-0.9	313, 285, 270, 258, 203, 153					

[a] The references described compounds found in the literature for leaves, culms, and shoots of several bamboos' plants and database sources such as PubChem, METLIN, KEGG, GNPS; [st] The identification of compounds has been verified by using authentic standards; [sh] shoulder peak; *m/z* data were obtained by UHPLC-QTOF-MS based on time and peak areas. λ_{max}: UV maximum absorbance wavelength. Phytochemicals were annotated with identification confidence levels as recommended by the Metabolomics Standards Initiative (MSI) according to: Level 0: *Unambiguous 3D Structure: isolated, pure compound, including full stereochemistry*; Level 1: *Confident 2D structure: uses reference standard match or full 2D structure elucidation;* Level 2: *Probable structure: matched to literature data or databases by diagnostic evidence*, Level 3: *Possible structure or class: most likely structure, isomers possible, substance class or substructure match*; Level 4: *Unknown feature of interest*.[21]

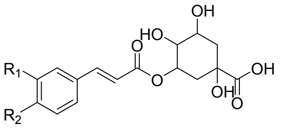

2 R_1 = OH; R_2 = OH
18 R_1 = OCH_3; R_2 = OH
20 R_1 = H; R_2 = OH

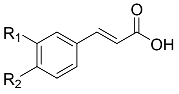

3 R_1 = OH; R_2 = OH
8 R_1 = H; R_2 = OH

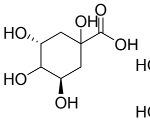

15

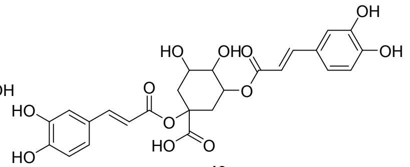

19

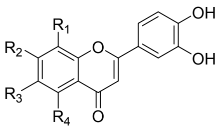

21 R_1 = 8-*C*-pentoside; R_2 = OH; R_3 = 6-*C*-hexoside; R_4 = OH
44 R_1 = H; R_2 = OCH_3; R_3 = 6-*C*-glucoside; R_4 = OH
49 R_1 = H; R_2 = OH; R_3 = H; R_4 = OH

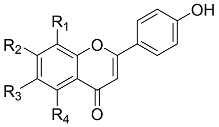

23 R_1 = 8-*C*-glucoside; R_2 = OH; R_3 = 6-*C*-glucoside; R_4 = OH
25 R_1 = 8-*C*-galactoside; R_2 = OH; R_3 = 6-*C*-galactoside; R_4 = OH
29 R_1 = 8-*C*-glucoside-2'-*O*-rhamnoside; R_2 = OH; R_3 = H; R_4 = OH
30 R_1 = H; R_2 = 7-*O*-glucoside; R_3 = 6-*C*-glucoside; R_4 = OH
31 R_1 = 8-*C*-glucoside; R_2 = OH; R_3 = 6-*C*-arabinoside; R_4 = OH
32 R_1 = 8-*C*-xyloside; R_2 = OH; R_3 = 6-*C*-glucoside; R_4 = OH
34 R_1 = H; R_2 = OCH_3; R_3 = 6-*C*-glucoside-2''-*O*-glucoside; R_4 = OH
35 R_1 = 8-*C*-rhamnoside; R_2 = OH; R_3 = 6-*C*-glucoside; R_4 = OH
42 R_1 = H; R_2 = 7-*O*-disaccharoside; R_3 = H; R_4 = OH
43 R_1 = H; R_2 = 7-*O*-disaccharoside; R_3 = H; R_4 = OH
48 R_1 = H; R_2 = 7-*O*-hexoside; R_3 = 6-*C*-hexoside; R_4 = OH
54 R_1 = H; R_2 = OH; R_3 = H; R_4 = OH

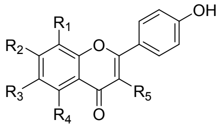

36 R_1 = H; R_2 = 7-*O*-neohesperidoside; R_3 = H; R_4 = OH; R_5 = OH
37 R_1 = H; R_2 = 7-*O*-pentoside; R_3 = H; R_4 = OH; R_5 = 3-*O*-disaccharoside
38 R_1 = H; R_2 = OH; R_3 = H; R_4 = OH; R_5 = 3-*O*-disaccharoside
57 R_1 = H; R_2 = OH; R_3 = H; R_4 = OH; R_5 = OH
58 R_1 = H; R_2 = OH; R_3 = OCH_3; R_4 = OH; R_5 = OCH_3

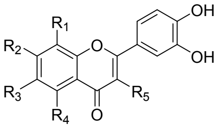

39 R_1 = H; R_2 = OH; R_3 = H; R_4 = OH; R_5 = 3-*O*-hexoside
40 R_1 = H; R_2 = OH; R_3 = H; R_4 = OH; R_5 = 3-*O*-galactoside

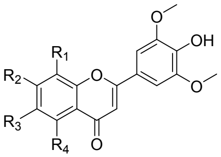

51 R_1 = H; R_2 = 7-*O*-glucoside; R_3 = H; R_4 = OH
60 R_1 = H; R_2 = OH; R_3 = H; R_4 = OH

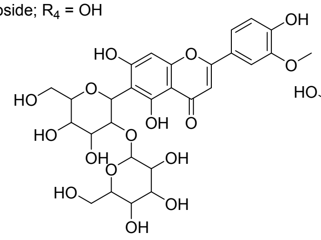

33

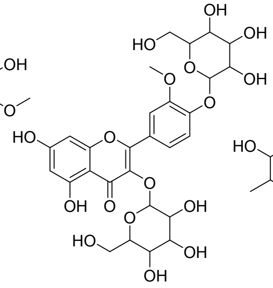

41

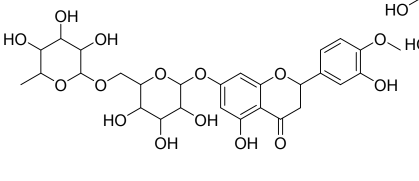

45

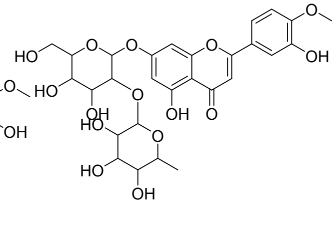

46

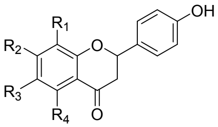

52 R_1 = H; R_2 = 7-*O*-disaccharoside; R_3 = H; R_4 = OH
53 R_1 = H; R_2 = 7-*O*-hexoside; R_3 = H; R_4 = OH

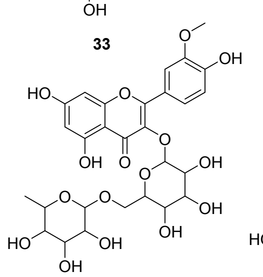

47

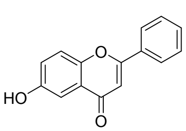

50

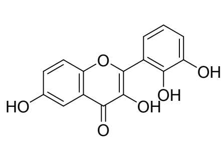

55

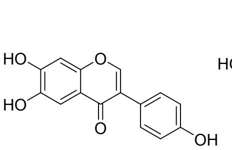

56

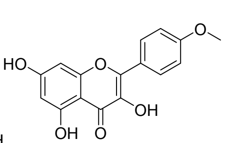

59

Figure S2. Chemical structures of the metabolites were found in BLEs.

Compound 2

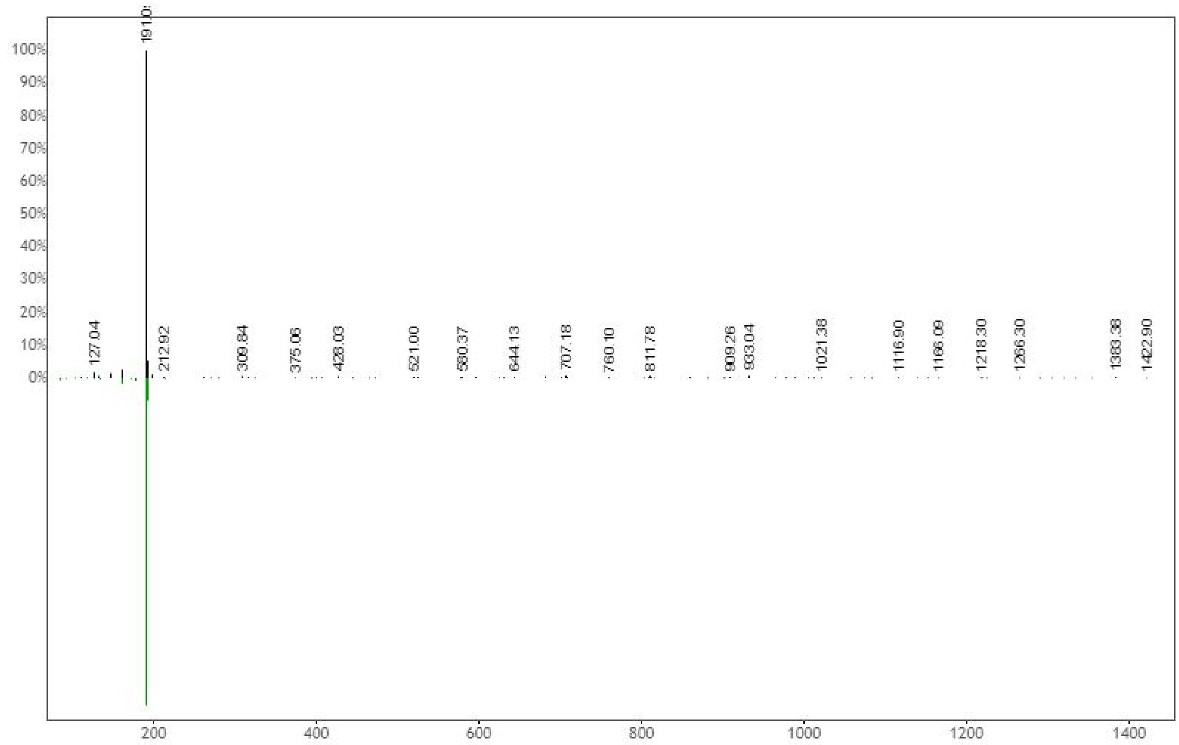

Compound 8

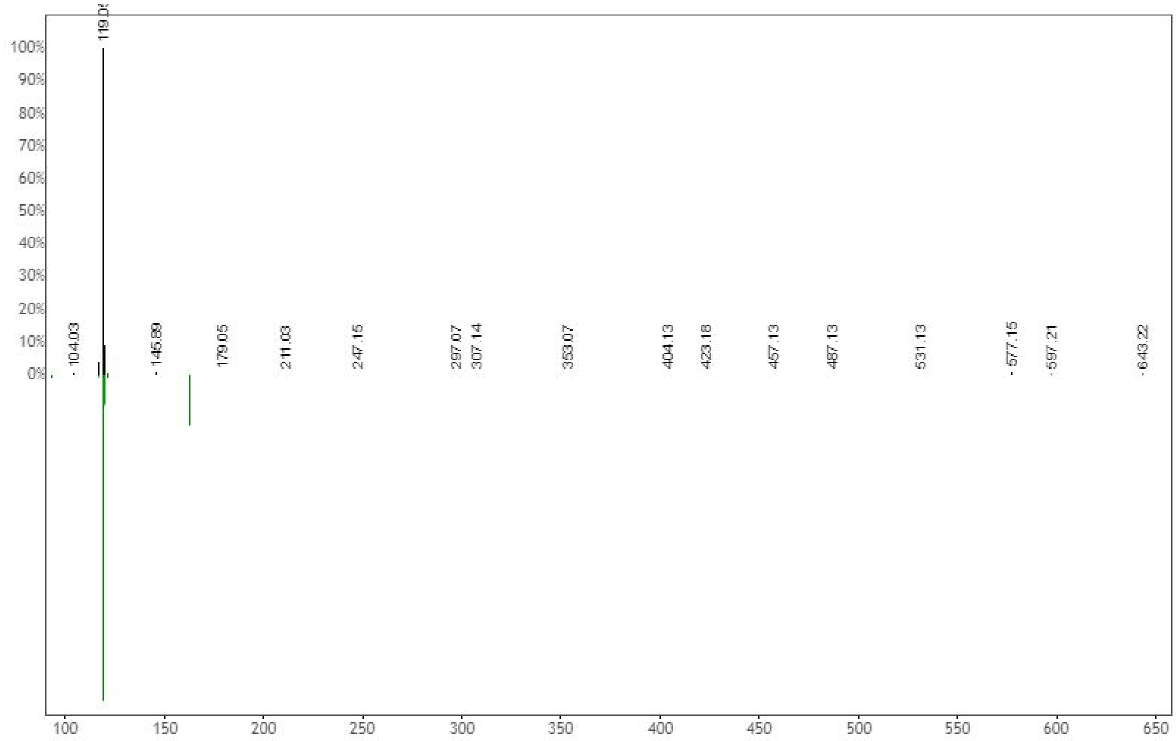

Compound 18

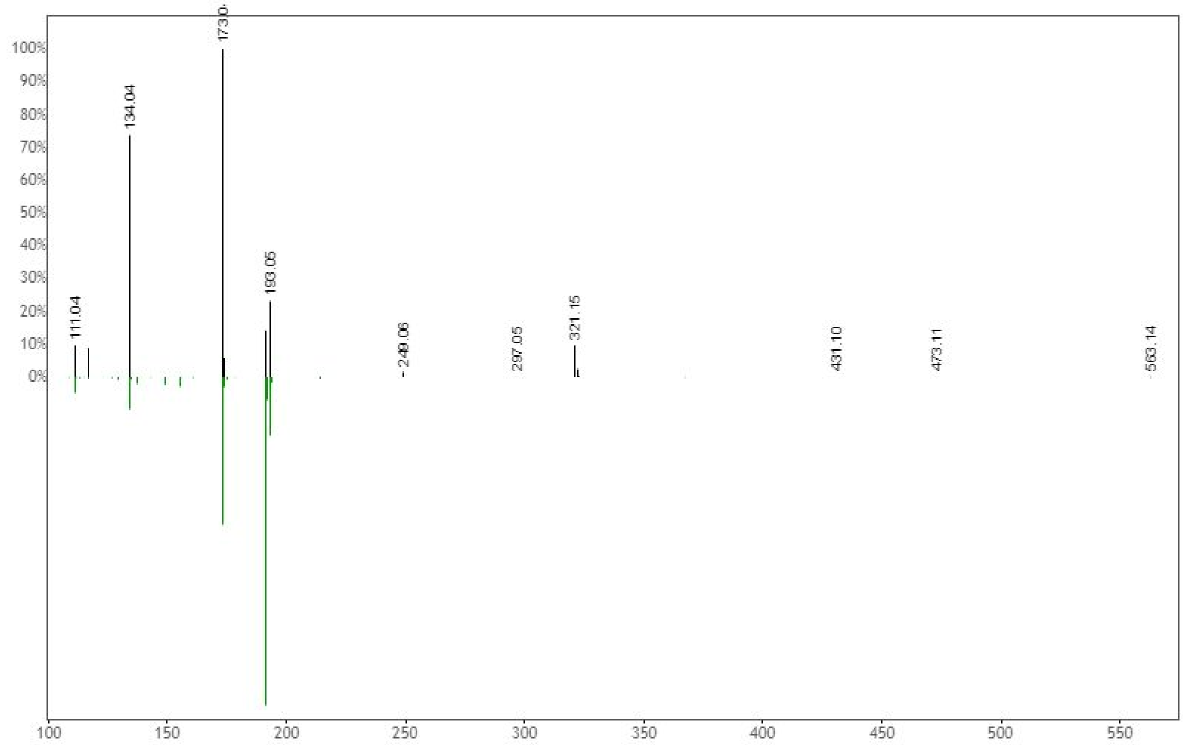

Compound 19

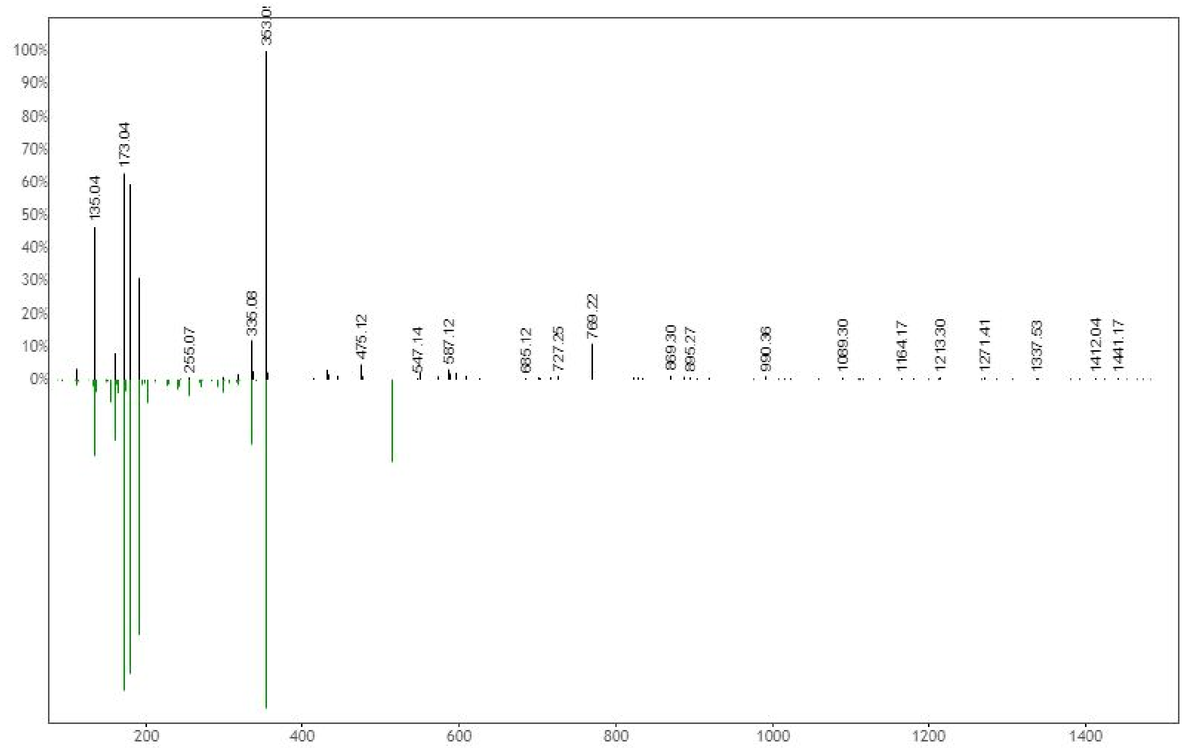

Compound 20

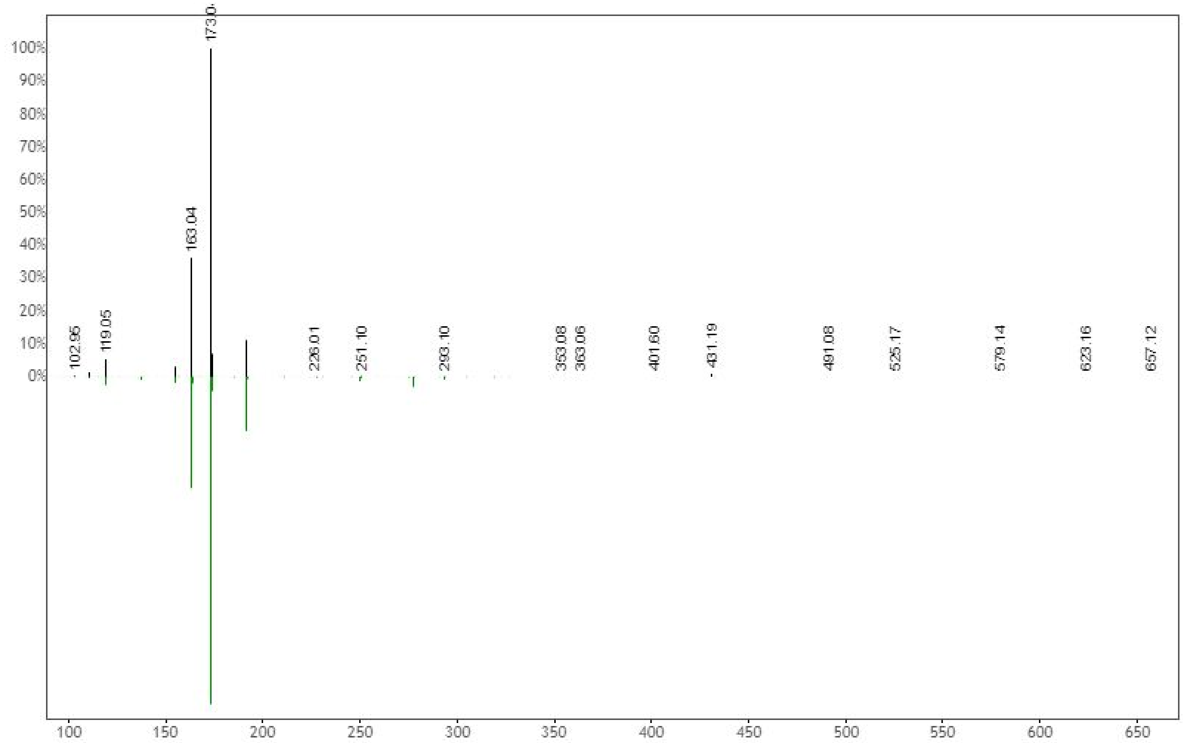

Compound 23

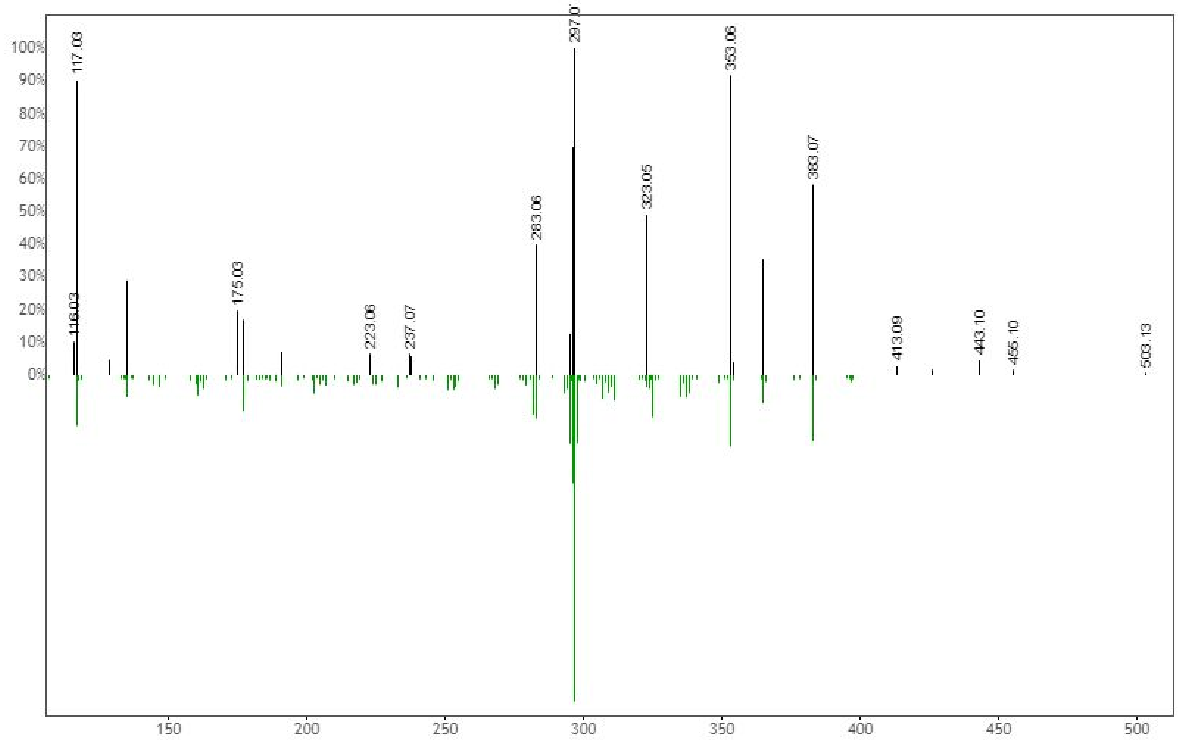

Compound 25

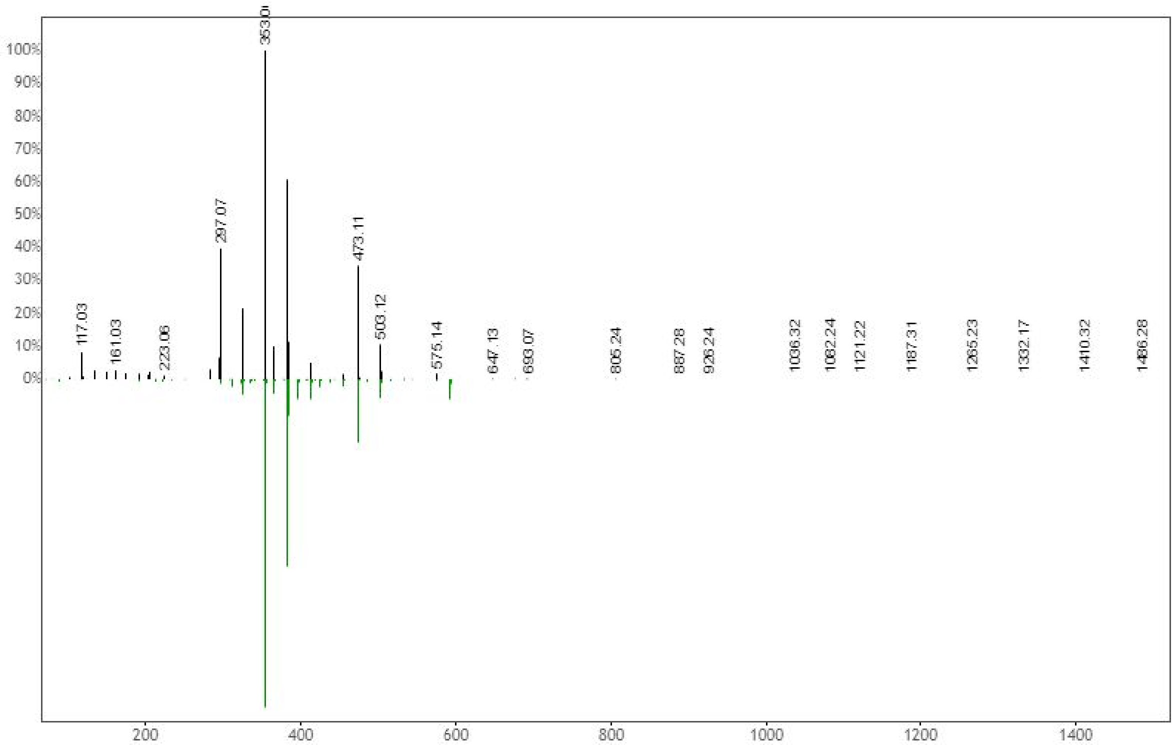

Compound 29

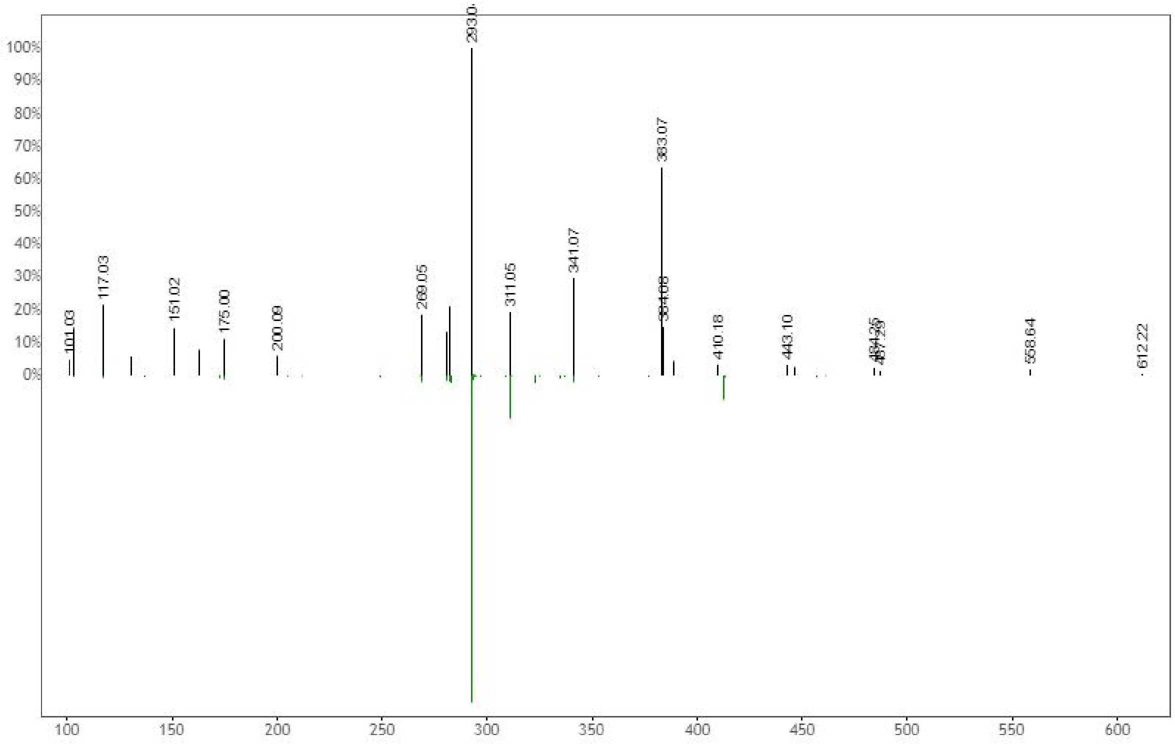

Compound 30

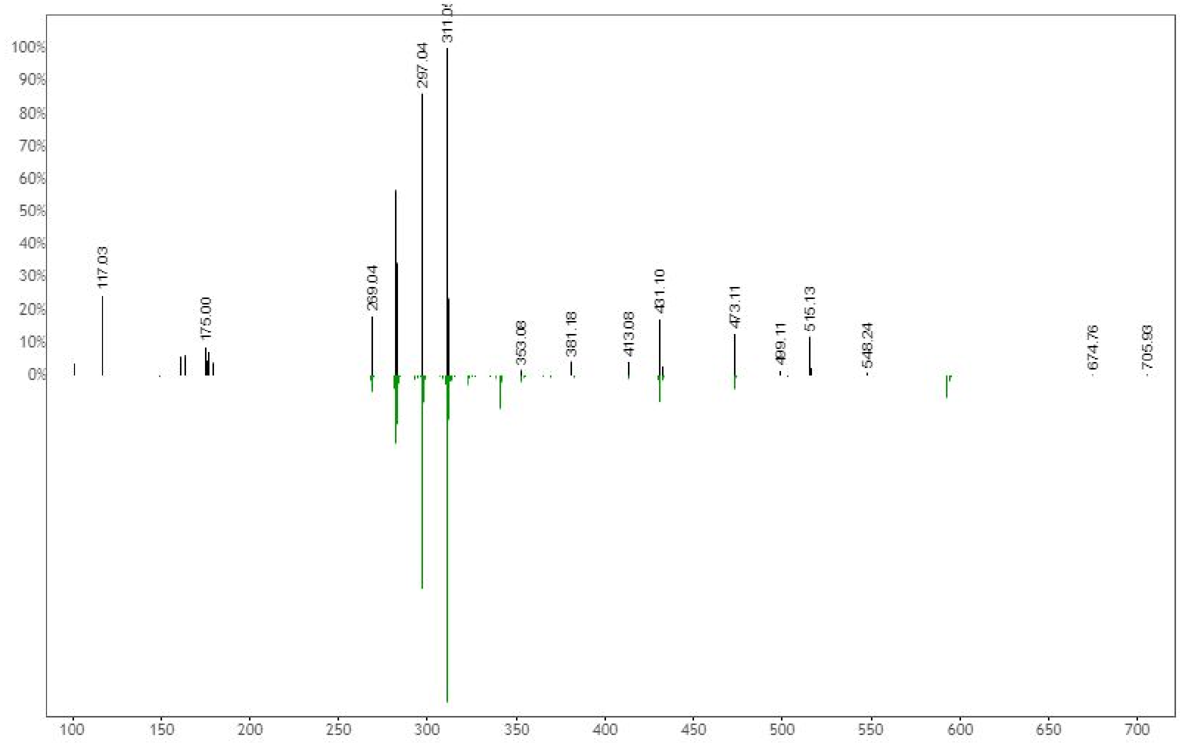

Compound 31

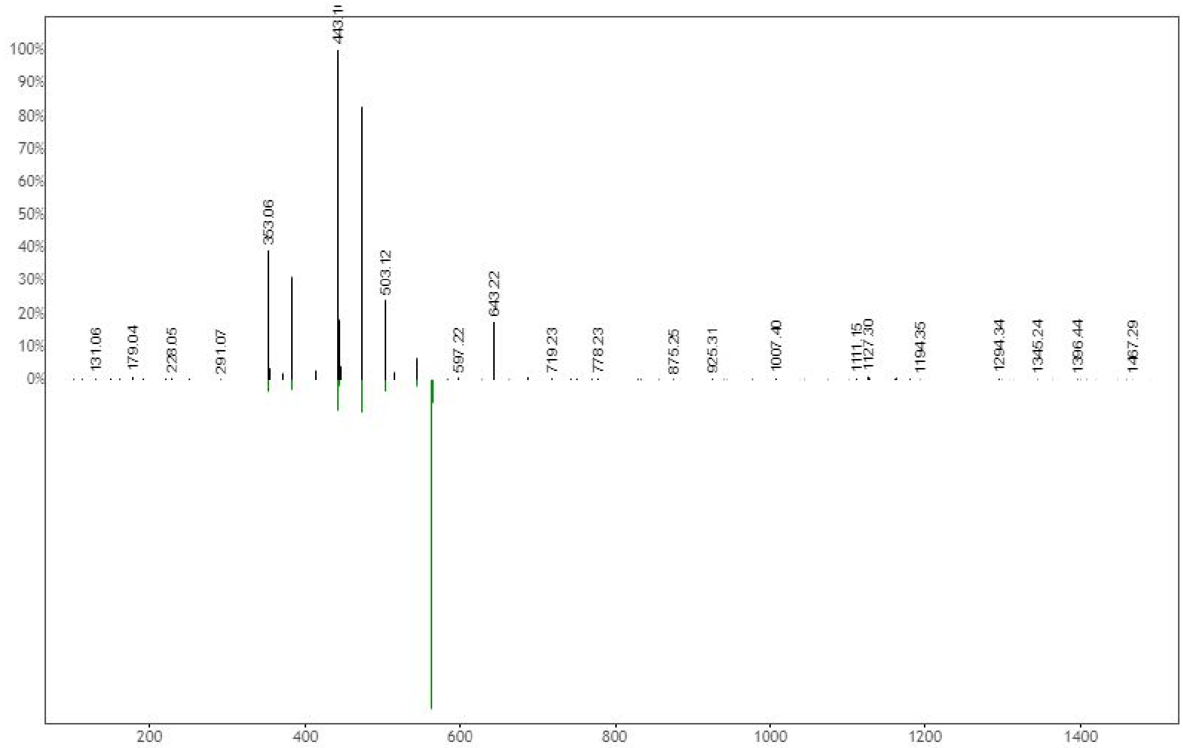

Compound 36

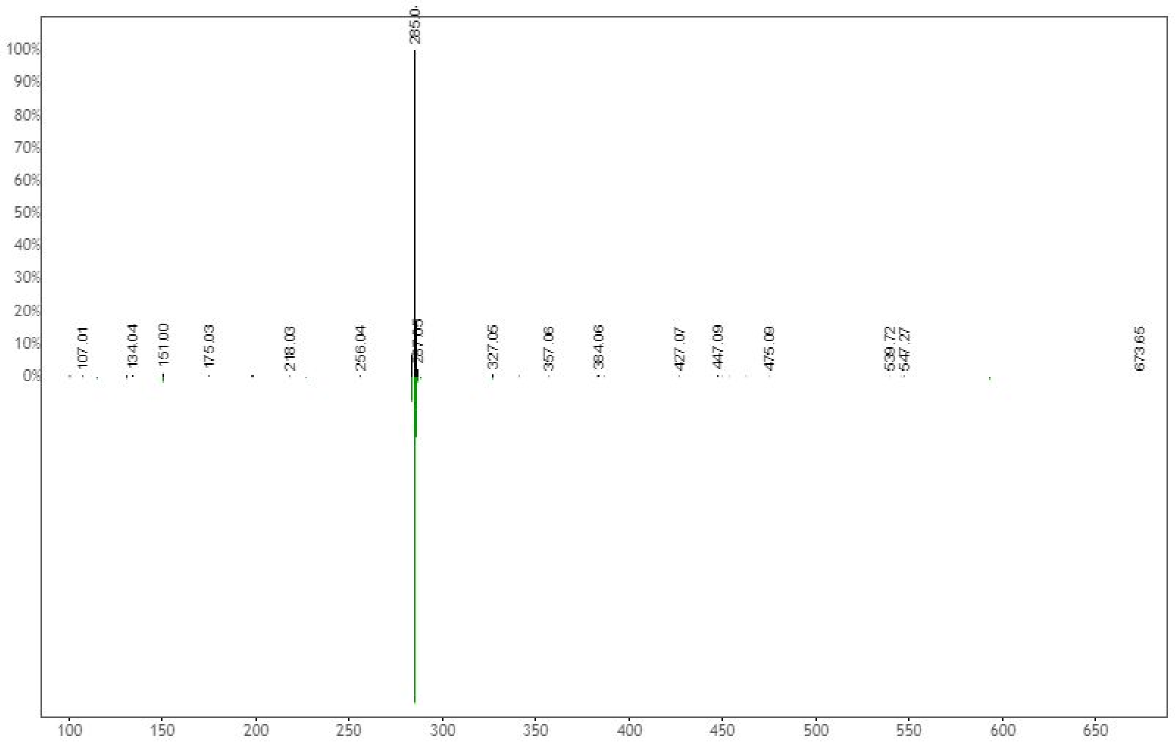

Figure S3. MS^2 spectrum match feature of GNPS showing the similarity of fragments patterns of the experimental and library data.

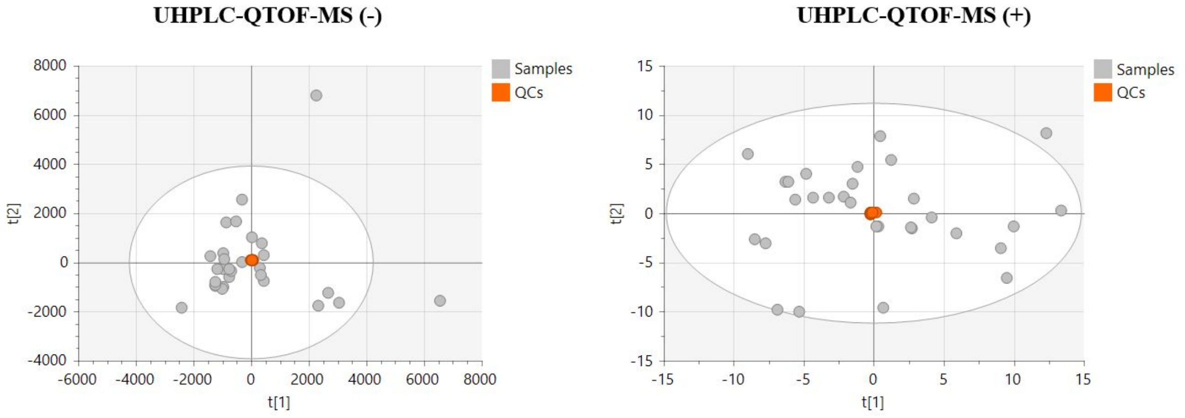

Figure S4. PCA score plot including Quality Controls (QCs) and all samples from UHPLC-QTOF-MS analysis in both negative and positive ion mode.

Table S2. List and information on the environmental variables of collection of the *Guadua* species used for the study.

Species	Sample code	Location	Collection date	Latitude	Longitude	Altitude	AMAT (°C)	AMET (°C)	AMIT (°C)	MP (mm)	RH (%)
G. aculeata Rupr. ex E. Fourn.	*Gac-Q1*	Quindío	12/2021	4.52086	-75.80102	1256	28.0	24.0	21.0	200-300	82.0
G. amplexifolia J.Presl	*Gam-Q1*	Quindío	03/2021	4.52083	-75.80108	1256	28.0	24.0	21.0	200-300	82.0
	Gam-Q2	Quindío	12/2021	4.52083	-75.80108	1256	28.0	24.0	21.0	200-300	82.0
	Gam-Q3	Quindío	12/2021	4.52083	-75.80108	1256	28.0	24.0	21.0	200-300	82.0
G. angustifolia Kunth	*Gan-C1*	Cundinamarca	09/2020	5.18127	-74.19519	1343	31.5	25.5	23.5	100-200	82.5
	Gan-C2	Cundinamarca	09/2020	5.18127	-74.19519	1343	31.5	25.5	23.5	100-200	82.5
	Gan-C3	Cundinamarca	09/2020	5.18127	-74.19519	1343	31.5	25.5	23.5	100-200	82.5
	Gan-C4	Cundinamarca	10/2022	5.18127	-74.19519	1343	31.5	25.5	23.5	100-200	82.5
	Gan-C5	Cundinamarca	09/2020	5.18127	-74.19519	1343	31.5	25.5	23.5	100-200	82.5
	Gan-C6	Cundinamarca	09/2020	5.18127	-74.19519	1343	31.5	25.5	23.5	100-200	82.5
	Gan-C7	Cundinamarca	10/2021	5.18127	-74.19519	1343	31.5	25.5	23.5	100-200	82.5
	Gan-N1	Nariño	06/2021	1.37383	-77.27988	1857	20.6	17.5	12.3	100-200	60.0
	Gan-N2	Nariño	12/2021	1.32755	-77.48088	1720	21.8	16.8	13.6	100-200	65.0
	Gan-N3	Nariño	12/2021	1.32755	-77.48088	1720	21.8	16.8	13.6	100-200	65.0
	Gan-N4	Nariño	01/2022	1.32755	-77.48088	1720	21.8	16.8	13.6	100-200	65.0
	Gan-N5	Nariño	01/2022	1.54900	-78.67738	21	28.0	26.0	24.0	200-300	85.0
	Gan-N6	Nariño	01/2022	1.54900	-78.67738	21	28.0	26.0	24.0	200-300	85.0
	Gan-Q1	Quindío	12/2021	4.52053	-75.79973	1256	28.0	24.0	21.0	200-300	82.0
	Gan-Q2	Quindío	04/2022	4.52053	-75.79973	1256	28.0	24.0	21.0	200-300	82.0
G. angustifolia Kunth biotype San Calixto	*Ganc-Q1*	Quindío	04/2022	4.52063	-75.80041	1256	29.0	23.0	18.0	200-300	80.0

G. angustifolia var. *bicolor* Londoño	*Ganb-Q1*	Quindío	04/2022	4.52018	-75.80056	1256	29.0	23.0	18.0	200-300	80.0
G. incana Londoño	*Gin-P1*	Putumayo	12/2021	1.17730	-76.66477	604	26.0	22.5	20.0	100-200	85.0
	Gin-Q1	Quindío	03/2021	4.52102	-75.80013	1256	28.0	24.0	21.0	200-300	82.0
	Gin-Q2	Quindío	12/2021	4.52102	-75.80013	1256	28.0	24.0	21.0	200-300	82.0
G. superba Huber	*Gsu-Q1*	Quindío	04/2022	4.52100	-75.80036	1256	29.0	23.0	18.0	200-300	80.0
G. uncinata Londoño & L.G.Clark	*Gun-Q1*	Quindío	03/2021	4.51816	-75.80033	1256	28.0	24.0	21.0	200-300	82.0
	Gun-Q2	Quindío	12/2021	4.51816	-75.80033	1256	28.0	24.0	21.0	200-300	82.0
	Gun-Q3	Quindío	12/2021	4.51816	-75.80033	1256	28.0	24.0	21.0	200-300	82.0
G. venezuelae Munro	*Gve-Q1*	Quindío	04/2022	4.52057	-75.80005	1256	29.0	23.0	18.0	200-300	80.0
G. weberbaueri Pilg.	*Gwe-Q1*	Quindío	04/2022	4.52043	-75.80057	1256	29.0	23.0	18.0	200-300	80.0

Annual Maximum Temperature (AMAT), Annual Mean Temperature (AMET), Annual Minimum Temperature (AMIT), Monthly Precipitation (MP), and Relative Humidity (RH).

Table S3. Comparison of retention times of reference standards between HPLC-DAD and UHPLC-QTOF-MS analyses.

No	Chemical standard	Chemical class	UV λ_{max} (nm)	Molecular formula	t_R (min) HPLC[a]	t_R (min) UHPLC[b]	Exact mass	$[M-H]^-$	$[M+H]^+$
**1**	Gallic acid	Organic acid	275	$C_7H_6O_5$	4.36	1.09	170.0215	169.0132	-
**2**	Chlorogenic acid (CGA)	Cinnamic acid and derivatives	275sh, 325	$C_{16}H_{18}O_9$	7.72	2.42	354.3095	353.0863	-
**3**	Caffeic acid	Cinnamic acid and derivatives	275	$C_9H_8O_4$	9.08	2.55	180.0422	179.0344	-
**4**	Isoorientin	Flavone glycosides	274, 355	$C_{21}H_{20}O_{11}$	9.39	2.96	448.3775	447.0954	449.1094
**5**	Ampelopsin	Flavanonol aglycones	300	$C_{15}H_{12}O_8$	9.88	2.88	320.0532	319.0458	-
**6**	Rutin	Flavonol glycosides	274, 355	$C_{27}H_{30}O_{16}$	10.42	3.20	610.1533	609.1531	611.1603
**7**	Vitexin	Flavone glycosides	273, 335	$C_{21}H_{20}O_{10}$	10.56	3.26	432.1056	431.0923	433.1123
**8**	*p*-Coumaric acid	Cinnamic acid and derivatives	295sh, 310	$C_9H_8O_3$	11.15	3.36	164.0473	163.0390	-
**9**	Sinapinic acid	Cinnamic acid and derivatives	325	$C_{11}H_{12}O_5$	11.55	3.78	224.2101	223.0684	-
**10**	Morin	Flavonol aglycones	264, 354	$C_{15}H_{10}O_7$	14.79	4.19	302.0426	301.0351	303.0512
**11**	Coumarin	Coumarins	275, 325	$C_9H_6O_2$	15.19	4.35	146.1432	-	147.0377
**12**	Quercetin	Flavonol aglycones	275, 358	$C_{15}H_{10}O_7$	15.86	4.49	302.0426	301.0344	303.0494
**13**	Cinnamic acid	Cinnamic acid and derivatives	275	$C_9H_8O_2$	16.84	4.63	148.1597	147.0524	-
**14**	Naringenin	Flavanone aglycones	275, 325	$C_{15}H_{12}O_5$	17.75	4.93	272.0684	271.0611	273.0764

[a] Column: Luna C18 column (150 x 4.6 mm, 5 µm, 100 Å)
[b] Column: ACQUITY UPLC HSS T3 C18 column (2.1 × 100 mm, 1.8 µm)

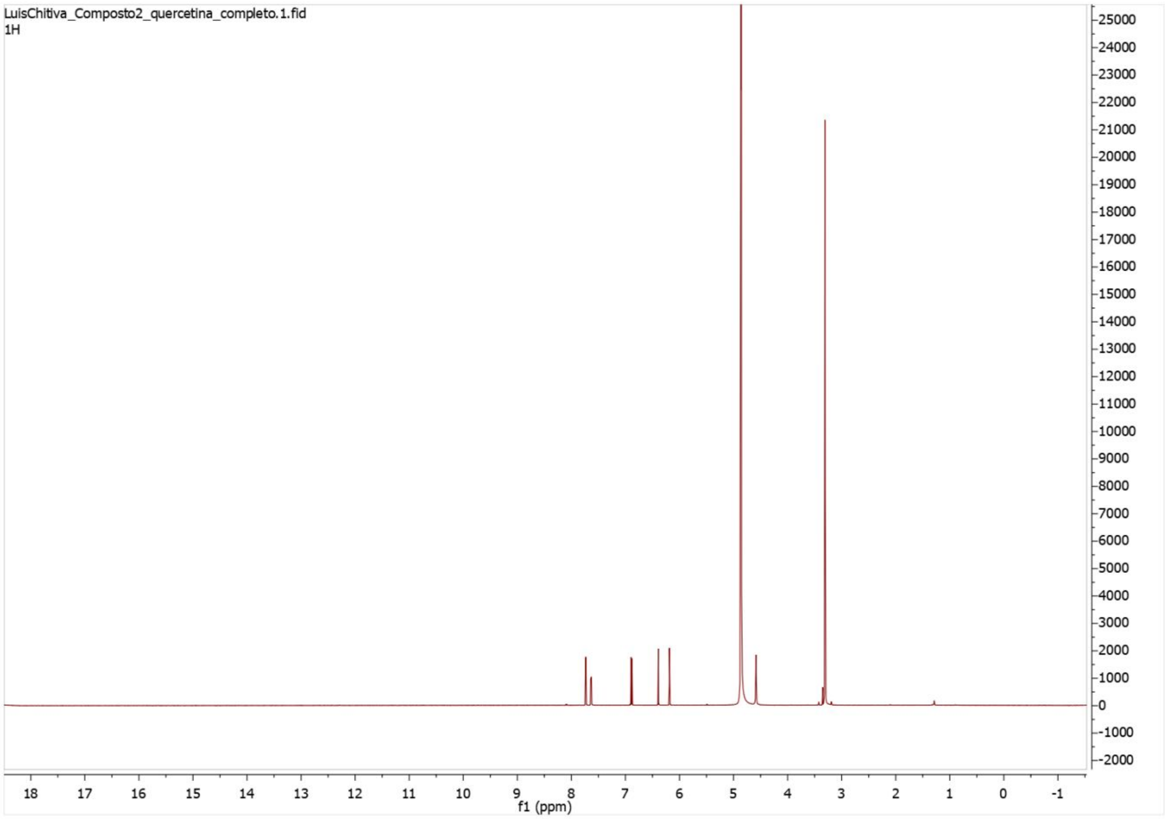

Figure S5. ^{1}H-NMR spectra obtained for quercetin.

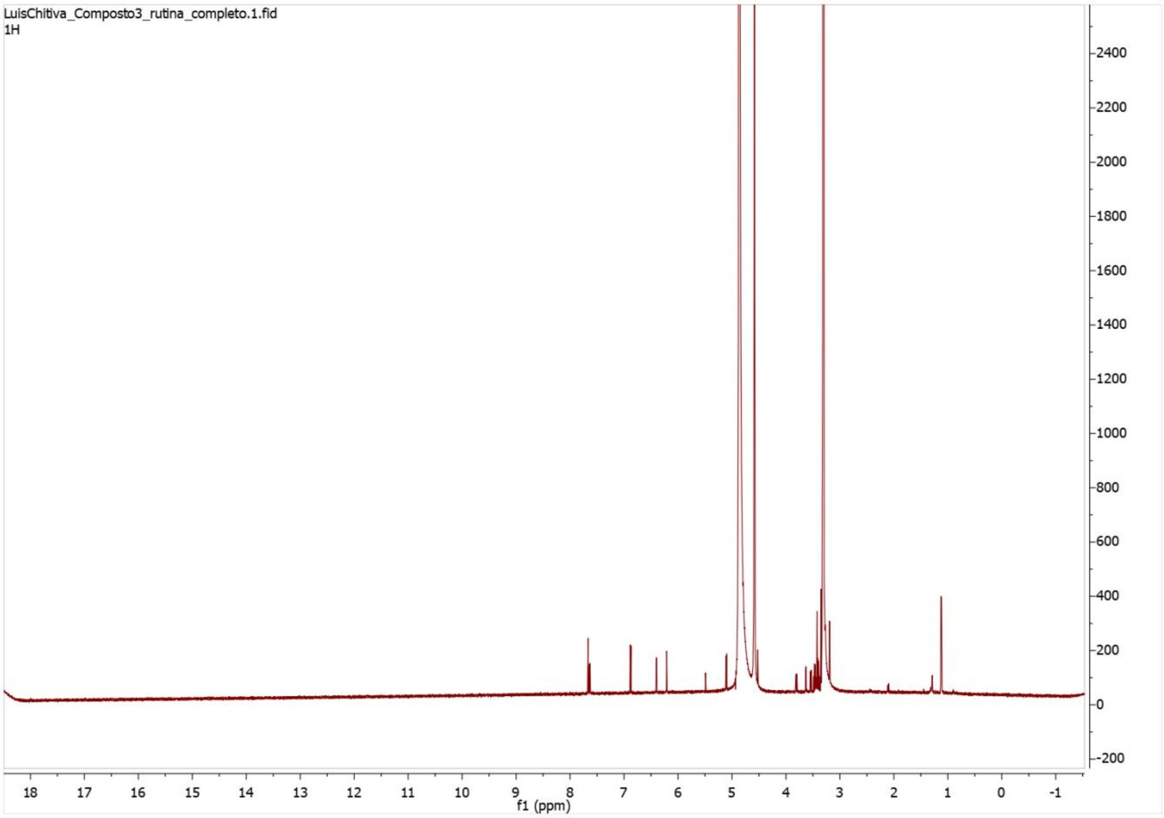

Figure S6. ^{1}H-NMR spectra obtained for rutin.

Table S4. Parameters used in the GNPS Classical Molecular Networking.

GNPS Classical Molecular Networking	
**Processing step**	**Parameters**
Basic options	Classical Molecular Networking<br>Precursor Ion Mass Tolerance: 0.02 Da<br>Fragment Ion Mass Tolerance: 0.02 Da
Advance Network Options	Min Pair Cos: 0.6<br>Network TopK: 10<br>Maximum Connected Component Size: 100<br>Minimun Matched Fragment Ions: 4<br>Minimum Cluster Size: 2<br>Maximum shift: 1999 Da<br>Run MSCluster: yes
Advance Library Search Options	Library Search Min Matched Peaks: 4<br>Search Analogs: Do Search<br>Score Threshold: 0.6<br>Maximum Analog Search Mass Difference: 100 Da

Table S5. Parameters used in the MZmine 2.53 software for processing the data obtained by UHPLC-QTOF-MS.

**MZmine version 2.53 software**	
**Preprocessing step**	**Parameters**
**Feature detection**	*Mass Detection*<br>MS level: 1<br>Set filters<br>Retention time: 0.2 - 10 min<br>Mass detector: Centroid<br>Noise level: 1E3<br>MS level: 2<br>Set filters<br>Retention time: 0.2 - 10 min<br>Mass detector: Centroid<br>Noise level: 1E2
**Chromatogram builder**	*ADAP Chromatogram Builder*<br>MS level: 1<br>Min group size in # of scans: 5<br>Group intensity threshold: 1E3<br>Min highest intensity: 3E3<br>*m/z* tolerance: 0 *m/z* or 20 ppm
**Deconvolution**	*Chromatogram deconvolution*<br>Algorithm: Baseline cut-off<br>Min peak height: 1E3<br>Peak duration range (min): 0 to 3 min<br>Baseline level: 1E3<br>*m/z* range for MS2 scan pairing (Da): 0.01<br>RT range for MS2 scan pairing (min): 0.02
**Isotopes**	*Isotopic peaks grouper*<br>*m/z* tolerance: 0 *m/z* or 20 ppm<br>Retention time tolerance: 0.02 min<br>Max charge: 2<br>Representative isotope: Most intense
**Alignment**	*Join aligner*<br>*m/z* tolerance: 0 *m/z* or 20 ppm<br>Weight for *m/z*: 75<br>Retention time tolerance: 0.25 absolute (min)<br>Weight for RT: 25
**Filtering**	*Feature list rows filter*<br>Minimum peaks in a row (X): 1<br>Minimum peaks in an isotope pattern (X): 1<br>*m/z* range: 75 to 2000<br>Reset peak number ID: True<br>Keep only peaks with MS2 scan (GNPS): (X)
**Gap Filling**	*Peak finder*<br>Intensity tolerance: 10%<br>*m/z* tolerance: 0 *m/z* or 10 ppm<br>RT tolerance: 0.05 min

**Export**	*Export to CSV file*<br>(X) Export row<br>(X) Export row *m/z*<br>(X) Export row retention time<br>(X) Peak area
**Formula prediction**	Charge: 2<br>Ionization type: M-H<br>*m/z* tolerance: 0.01 *m/z* or 10 ppm<br>Max best formulas per peak: 3<br>O 50<br>H 100<br>C 100

Table S6. Parameters used in the NMRProcFlow software for processing the data obtained by ^{1}H-NMR.

**NMRProcFlow**	
**Preprocessing step**	**Parameters**
**PPM Calibration**	*Range of the PPM reference:*<br>Not applicable<br>*PPM value of the center of resonance:*<br>0<br>*noisy PPM range:*<br>10.5 10.2
**Normalization**	*Normalization Method:*<br>Constant Sum Normalization<br>*Reference PPM ranges:*<br>Not applicable
**Baseline correction**	*Type of Correction:*<br>Local Correction<br>*noisy PPM range:*<br>10.5 10.2<br>*Restricted PPM range:*<br>Not applicable<br>*Level of Correction:*<br>1<br>*Order:*<br>1
**Alignment**	*Align. Method:*<br>Least Square<br>*Relative max. shift:*<br>0.05<br>*Reference spectrum:*<br>Auto reference<br>*PPM Ranges to align:*<br>Not applicable
**PPM shift**	*PPM shift value:*<br>0<br>*PPM Ranges to shift:*<br>Not applicable
**Zeroing**	*PPM Ranges to clean:*<br>4.99 4.75
**Bucketing**	

Bucketing Method<br>*Intelligent Bucketing*	*Resolution Factor:*<br>0.5<br>*SNR threshold:*<br>3<br>*noisy PPM range:*<br>10.5 10.2<br>*PPM Ranges:*<br>10.0 0.0
**Data export**	
Data matrix	*Export Format:*<br>Comma Separator Value (CSV)<br>*SNR threshold:*<br>3<br>*noisy PPM range:*<br>10.5 10.2<br>*Normalization Method:*<br>Constant Sum Normalization<br>*PPM range of the Reference:*<br>Not applicable
**Buckets table**	*Export Format:*<br>Comma Separator Value (CSV)

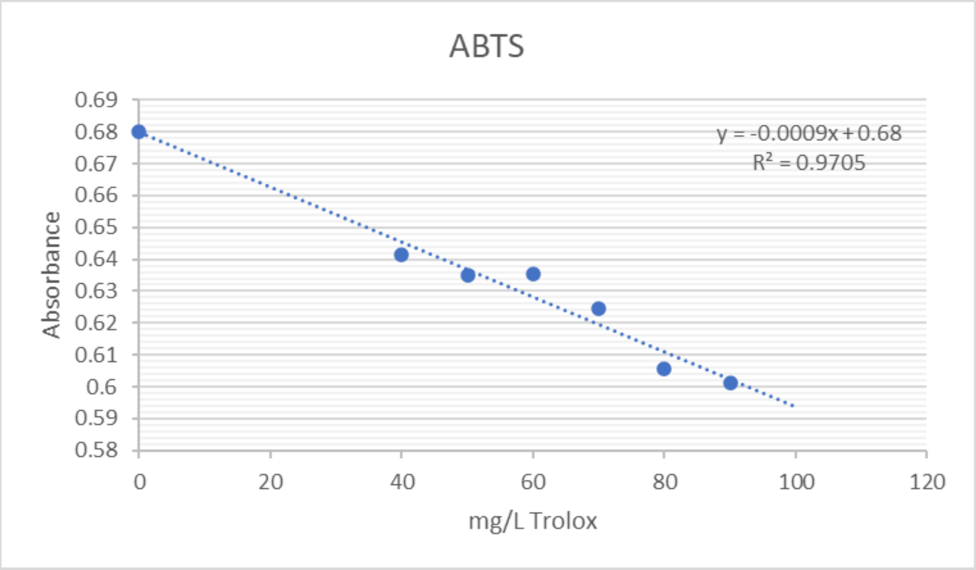

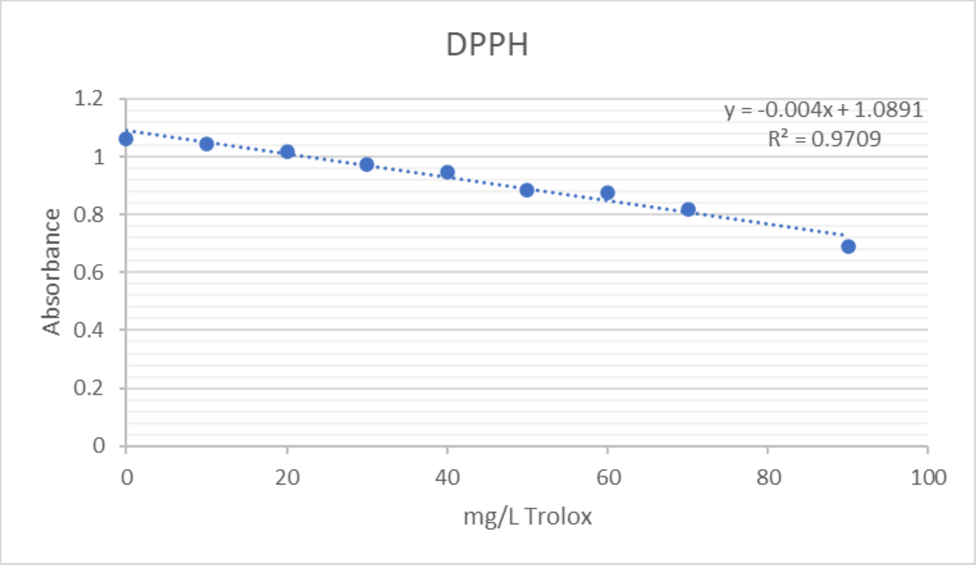

Figure S7. Calibration curves obtained for the determination of antioxidant capacity by ABTS and DPPH.

References

(1) Chitiva, L. C.; Lozano-Puentes, H. S.; Londoño, X.; Leão, T. F.; Cala, M. P.; Ruiz-Sanchez, E.; Díaz-Ariza, L. A.; Prieto-Rodríguez, J. A.; Castro-Gamboa, I.; Costa M, G. Untargeted Metabolomics Approach and Molecular Networking Analysis Reveal Changes in Chemical Composition under the Influence of Altitudinal Variation in Bamboo Species. *Front. Mol. Biosci.* **2023**, *10*, 1192088. https://doi.org/10.3389/fmolb.2023.1192088.

(2) Cao, X.; Zhang, Y.; Xun, H.; Wang, J.; Tang, F. High-Yield Recovery of Antioxidant Compounds from *Bambusa Chungii* Culms Using Pressurized Hot Water Extraction. *Antioxidants* **2022**, *11* (11). https://doi.org/10.3390/antiox11112231.

(3) Jiang, L.; Belwal, T.; Huang, H.; Ge, Z.; Limwachiranon, J.; Zhao, Y.; Li, L.; Ren, G.; Luo, Z. Extraction and Characterization of Phenolic Compounds from Bamboo Shoot Shell under Optimized Ultrasonic-Assisted Conditions: A Potential Source of Nutraceutical Compounds. *Food Bioprocess Technol.* **2019**, *12*, 1741–1755.

(4) Wang, Y.; Chen, J.; Wang, D.; Ye, F.; He, Y.; Hu, Z.; Zhao, G. A Systematic Review on the Composition, Storage, Processing of Bamboo Shoots: Focusing the Nutritional and Functional Benefits. *J. Funct. Foods* **2020**, *71*, 104015.

(5) Van Hoyweghen, L. Van; Beer, T. De; Deforce, D.; Heyerick, A. Phenolic Compounds and Anti-oxidant Capacity of Twelve Morphologically Heterogeneous Bamboo Species. *Phytochem. Anal.* **2012**, *23* (5), 433–443.

(6) Van Hoyweghen, L.; Deforce, D.; Heyerick, A. Antioxidant Capacity and Secondary Metabolites of 12 Bamboo Species. In *I International Symposium on Genetic Resources of Bamboos and Palms and III International Symposium on Ornamental Palms 1003*; 2010; pp 107–112.

(7) Haldipur, A. C.; Srividya, N. A Comparative Evaluation of in Vitro Antihyperglycemic Potential of Bamboo Seed Rice (*Bambusa Arundinacea*) and Garudan Samba (*Oryza Sativa*): An Integrated Metabolomics, Enzymatic and Molecular Docking Approach. *J.*

Cereal Sci. **2021**, *99*, 103200. https://doi.org/10.1016/j.jcs.2021.103200.

(8) Gagliano, J.; Altenhofen, S.; Nabinger, D. D.; Gusso, D.; Kuhl-Silva, J. M.; Anselmo-Moreira, F.; Yamaguchi, L. F.; Kato, M. J.; Bonan, C. D.; Furlan, C. M. Are Brazilian Bamboo Species Helpful for Cognition and Memory? *Phytomedicine Plus* **2022**, *2* (1). https://doi.org/10.1016/j.phyplu.2021.100183.

(9) Akhtar, J.; Patowary, L. *Bambusa Vulgaris*: A Comprehensive Review of Its Traditional Uses, Phytochemicals and Pharmacological Activities. *Sci. Phytochem.* **2022**, *1* (2), 11–21. https://doi.org/10.58920/sciphy01020011.

(10) Hu, C.; Zhang, Y.; Kitts, D. D. Evaluation of Antioxidant and Prooxidant Activities of Bamboo *Phyllostachys Nigra* Var. *Henonis* Leaf Extract in Vitro. *J. Agric. Food Chem.* **2000**, *48* (8), 3170–3176. https://doi.org/10.1021/jf0001637.

(11) Ni, Q.; Xu, G.; Wang, Z.; Gao, Q.; Wang, S.; Zhang, Y. Seasonal Variations of the Antioxidant Composition in Ground Bamboo *Sasa Argenteastriatus* Leaves. *Int. J. Mol. Sci.* **2012**, *13* (2), 2249–2262. https://doi.org/10.3390/ijms13022249.

(12) Ma, N. H.; Guo, J.; Chen, S. H. X.; Yuan, X. R.; Zhang, T.; Ding, Y. Antioxidant and Compositional HPLC Analysis of Three Common Bamboo Leaves. *Molecules* **2020**, *25* (2), 409. https://doi.org/10.3390/molecules25020409.

(13) Tanaka, A.; Zhu, Q.; Tan, H.; Horiba, H.; Ohnuki, K.; Mori, Y.; Yamauchi, R.; Ishikawa, H.; Iwamoto, A.; Kawahara, H.; Shimizu, K. Biological Activities and Phytochemical Profiles of Extracts from Different Parts of Bamboo (*Phyllostachys Pubescens*). *Molecules* **2014**, *19* (6), 8238–8260. https://doi.org/10.3390/molecules19068238.

(14) Gagliano, J.; Anselmo-Moreira, F.; Sala-Carvalho, W. R.; Furlan, C. M. What Is Known about the Medicinal Potential of Bamboo? *Adv. Tradit. Med.* **2022**, *22* (3), 467–495. https://doi.org/10.1007/s13596-020-00536-5.

(15) Ni, Q.; Wang, Z.; Xu, G.; Gao, Q.; Yang, D.; Morimatsu, F.; Zhang, Y. Altitudinal Variation of Antioxidant Components and Capability in *Indocalamus Latifolius* (Keng) McClure Leaf. *J. Nutr. Sci. Vitaminol. (Tokyo).* **2013**, *59* (4), 336–342. https://doi.org/10.3177/jnsv.59.336.

(16) Wang, Y.; Zhang, Y.; Cheng, J.; Zhao, J.; Shi, R.; He, L.; Li, Q.; Chen, Y. Efficient Purification of Flavonoids from Bamboo Shoot Residues of *Phyllostachys Edulis* by Macroporous Resin and Their Hypoglycemic Activity. *Food Chem. X* **2022**, *16*, 100505.

(17) Silva de Oliveira, D. C. Composição Química e Atividades Biológicas de Extratos de *Guadua Angustifolia* Kunth, Universidade de São Paulo, 2019. https://www.teses.usp.br/teses/disponiveis/9/9138/tde-22122021-132604/pt-br.php.

(18) Morales Noreña, N.; Sanchez Vallejo, L. Contribución Al Estudio Fitoquímico de Las Hojas de *Guadua Angustifolia* Kunth, Universidad del Quindío, 2008.

(19) Li, X.; Tao, W.; Xun, H.; Yao, X.; Wang, J.; Sun, J.; Yue, Y.; Tang, F. Simultaneous Determination of Flavonoids from Bamboo Leaf Extracts Using Liquid Chromatography-Tandem Mass Spectrometry. *Rev. Bras. Farmacogn.* **2021**, *31*, 347–352.

(20) Cheng, Y.; Wan, S.; Yao, L.; Lin, D.; Wu, T.; Chen, Y.; Zhang, A.; Lu, C. Bamboo Leaf: A Review of Traditional Medicinal Property, Phytochemistry, Pharmacology, and Purification Technology. *J. Ethnopharmacol.* **2023**, *306*, 116166. https://doi.org/10.1016/j.jep.2023.116166.

(21) Blaženović, I.; Kind, T.; Ji, J.; Fiehn, O. Software Tools and Approaches for Compound Identification of LC-MS/MS Data in Metabolomics. *Metabolites*. 2018. https://doi.org/10.3390/metabo8020031.
